# Long non-coding RNA expression profiles of hepatitis C virus-related dysplasia and hepatocellular carcinoma

**DOI:** 10.18632/oncotarget.6087

**Published:** 2015-10-26

**Authors:** Haohai Zhang, Chengpei Zhu, Yi Zhao, Ming Li, Liangcai Wu, Xiaobo Yang, Xueshuai Wan, Anqiang Wang, Michael Q. Zhang, Xinting Sang, Haitao Zhao

**Affiliations:** ^1^ Department of Liver Surgery, Peking Union Medical College Hospital, Chinese Academy of Medical Sciences and Peking Union Medical College, Beijing, China; ^2^ School of Medicine, MOE Key Laboratory of Bioinformatics and Bioinformatics Division, Center for Synthetic and System Biology, Tsinghua University, Beijing, China; ^3^ Key Laboratory of Intelligent Information Processing, Institute of Computing Technology, Chinese Academy of Sciences, Beijing, China

**Keywords:** long non-coding RNAs, hepatitis C virus-related HCC, hepatocarcinogenic process, biomarkers

## Abstract

Recently, long non-coding RNAs (lncRNAs) were found to be implicated in cancer progression. However, the contributions of lncRNAs to Hepatitis C virus-related hepatocellular carcinoma (HCC) remain largely unknown. Here, we characterized lncRNA expression in 73 tissue samples from several different developmental stages of HCV-related hepatocarcinogenesis by repurposing microarray data sets. We found that the expression of 7 lncRNAs in preneoplastic lesions and HCC was significantly different. Among these significantly differently expressed lncRNAs, the lncRNA LINC01419 transcripts were expressed at higher levels in early stage HCC compared to dysplasia and as compared with early stage HCC, lncRNA AK021443 level increase in advanced stage HCC while lncRNA AF070632 level decrease in advanced stage HCC. Using quantitative real-time reverse-transcription PCR, we validated that LINC01419 was significantly overexpressed in HBV-related and HCV-related HCC when compared with matched non-tumor liver tissues. Moreover, functional predictions suggested that LINC01419 and AK021443 regulate cell cycle genes, whereas AF070632 is associated with cofactor binding, oxidation-reduction and carboxylic acid catabolic process. These findings provide the first large-scale survey of lncRNAs associated with the development of hepatocarcinogenesis and may offer new diagnostic biomarkers and therapeutic targets for HCV-related HCC.

## INTRODUCTION

Hepatocellular carcinoma (HCC) is one of the most frequently diagnosed cancers in the world and is the second leading cause of cancer-related deaths worldwide [1]. Despite improvements in HCC patient survival that have resulted from advances in surgical strategies and improved surveillance of patients with a high risk for HCC development, five-year survival rates remain at approximately 26% [2]. The major risk factors for the development of HCC include chronic liver disease as a result of infection with either the hepatitis B virus (HBV) or the hepatitis C virus (HCV) and exposure to carcinogens, such as aflatoxin B1 [3]. Chronic infection with HBV is related to HCC in developing countries, whereas chronic infection with HCV is the main risk factor in developed countries [4]. Hepatocellular carcinoma pathogenesis is a slow process that may take more than 30 years to develop after the initial diagnosis of chronic infection with HBV or HCV. During the long preneoplastic stage, genetic alteration progressively changes the hepatocellular phenotype, leading to cell death, cellular proliferation, dysplasia and neoplasia [5]. An investigation of the molecular events across this slow process could identify crucial genes involved in HCC pathogenesis [6–8]. Such genes are potential biomarkers for the early detection of HCC [8, 9] or for predicting the prognosis of patients with HCC [10–12]. Unfortunately, most studies to date have explored only protein-coding genes or miRNA sequences. However, in addition to approximately 20,000 protein-coding genes and encoded small RNA molecules, the human transcriptome comprises a large number of long, nonprotein-coding RNAs (long noncoding RNAs; lncRNAs) [13].

lncRNAs are defined as nonprotein-coding RNAs that are greater than 200 nucleotides in length [14]. Multiple studies have suggested that several lncRNAs participate in a variety of biological process [15–18] and are associated with human diseases [19, 20]. Furthermore, lncRNAs have been reported to be involved in HCC through various mechanisms, including epigenetic silencing, mRNA splicing and translation, lncRNA-protein interaction and genetic variation [21, 22]. Several lncRNAs serve as potential biomarkers for the detection or prognosis of HCC. For example, the HULC gene, located on chromosome 6p24.3, is significantly overexpressed in HCC tissues [23]. The expression of HULC can be up-regulated by the transcription factor CREB (cAMP responsive element binding protein) and the Hepatitis B virus X protein (HBX) [24, 25]. In addition, HULC expression in HCC tissue and plasma of HCC patients is associated with Edmondson histological grades and HBV status. Moreover, HULC detection frequencies in HCC patients are higher than those in healthy people. These characteristics suggest that HULC may act as a diagnostic and/or prognostic biomarker for HCC [26]. Another potential lncRNA biomarker for HCC is lncRNA-HEIH, whose expression in HBV-related HCC tissue was reported as an independent risk factor for recurrence-free survival (RFS) [27]. Moreover, lncRNA-HEIH was overexpressed in HCC tissue compared with adjacent normal liver tissue and was higher in cirrhotic liver tissue than in healthy liver tissue [27]. Taken together, these previous findings suggest that lncRNAs may play an important role in the pathogenesis of HCC. However, most previous studies have only examined lncRNA expression in either HBV-induced HCC [26–28] or in mixed etiology (HBV and HCV) HCC [29]. The specific role of lncRNAs in the pathogenesis of HCV-related HCC remains unclear. Thus, the goal of our study was to explore lncRNA expression at the different stages of HCV-induced hepatocarcinogenesis.

In this study, we examined lncRNA expression in 73 tissue samples, including healthy liver tissue, cirrhotic tissue, dysplastic nodules, and HCC samples. The cirrhotic tissue, dysplastic nodules, and HCC samples were obtained from patients with HCV infections. Our data show that several lncRNAs could serve as biomarkers for the detection or prognosis of HCV-related HCC. Furthermore, we predicted the functions of the lncRNAs by investigating coexpression networks of the differentially expressed lncRNAs and protein-coding genes.

## RESULTS

### Transcriptomic analysis of HCV-induced dysplasia and hepatocellular carcinoma

**Figure 1:**
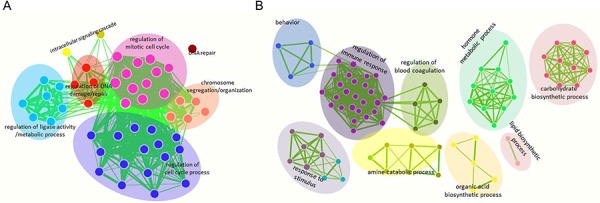
Functional enrichment maps of deregulated protein-coding genes in HCC The gene sets for GO terms were visualized using the Cytoscape Enrichment Map plugin. Each node represents a GO term. Node size is indicative of the number of genes in a set. The thickness of each line is indicative of the number of genes shared between the connected gene sets. The enriched gene sets of up-regulated protein-coding genes in HCC are shown in **A.** and the down-regulated protein-coding genes in HCC are shown in **B.**

Of the publicly available gene expression microarray data sets, we focused on repurposing microarray data sets comprising 75 samples that represent all stages of HCV-induced HCC. For these Affymetrix microarray platforms (HGU133plus2.0), we re-annotated the entire collection of probes using the ncFANs utility. As a result, we identified probes for 17,600 protein-coding genes and 2,863 lncRNAs in these published microarrays. Next, we reanalyzed the expression patterns of the protein-coding genes across 5 groups of patient samples: control, cirrhosis, dysplasia, early HCC and advanced HCC. Because HCC always occurs in cirrhotic livers, we compared the gene profiles associated with the preneoplastic lesion stage (cirrhosis and dysplasia stages) with those of HCC samples to identify the critical molecular events associated with HCC progression. Our results indicated that 585 protein-coding genes (Supplementary File 1) and 7 lncRNA genes (Table 1) were differentially expressed between preneoplastic lesions and HCC samples (*p* value < 0.05, fold change >2). Then, we performed a gene ontology (GO) analysis to enrich for gene sets based on differentially expressed protein-coding genes. We observed that genes that were up-regulated in HCC samples mainly participated in mitosis and the cell cycle (Fig. 1A, Supplementary Table 1), whereas genes that were down-regulated in HCC samples were associated with other functional capacities, such as response to steroid hormone stimulus, immune and inflammatory responses and normal liver function, which includes glucose and organic acid metabolism, organic acid biosynthesis and coagulation (Fig. 1B, Supplementary Table 2).

**Table 1:** Summary of lncRNAsthat are differentially expressed between preneoplasticlesions and HCC

Gene symbol	Chromosomal location	Preneoplastic lesions vs. HCC	
		Regulation	*P*-value
LINC01419	Chr8:84315993-84321132(+)	Up	0.000633277280341197
BC014579	Chr10:5015558-5017273(+)	Up	0.0000959501811649745
AK021443	Chr10:96372044-96373661(+)	Up	6.56187823907914E-06
RP11-401P9.4	Chr16:50679720-50683160(+)	Up	0.00383700431326679
RP11-304 L19.5	Chr16:2204798-2205359(−)	Up	0.0000692618363817383
CTB-167B5.2	Chr7:87900207-87903065(−)	Down	1.43661312617711E-09
AF070632	Chr1:112313293-112315191(−)	Down	2.60357616541779E-06

### Deregulated lncRNAs in HCC

**Figure 2:**
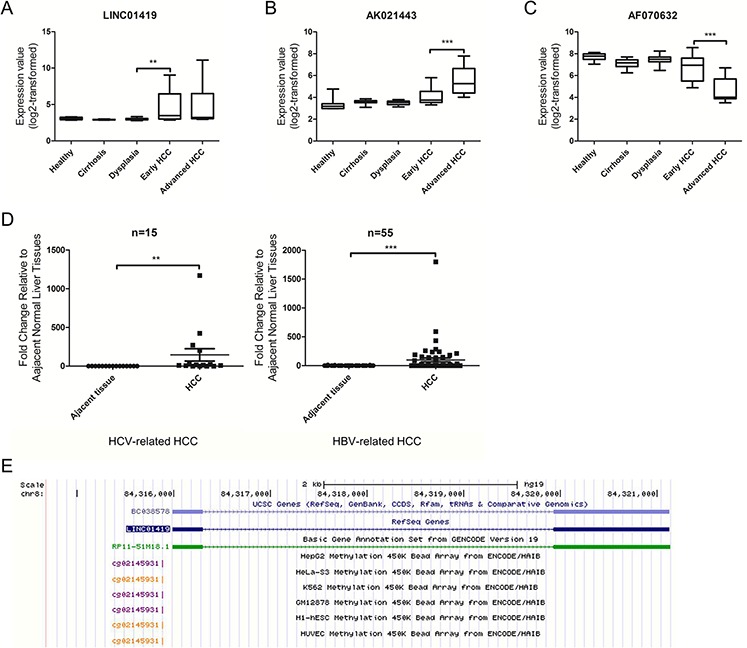
Expression of deregulated lncRNAs in HCC Expression values of LINC01419 (A), AK021443 (B), and AF070632 (C) in the five stages of HCC based on microarray data from73 patient samples (10 healthy liver samples, 12 cirrhotic liver samples, 17 dysplastic liver samples, 16 early HCC samples, and 17 advanced HCC samples). For **A, B,** and **C.** the *p*-values were calculated using the Mann-Whitney test. **D.** LINC01419 expression in 55 pairs of HBV-related HCC and 15 pairs of HCV-related HCC. The expression level of LINC01419 was analyzed by qRT-PCR. The *p*-values were calculated using the Wilcoxon signed-rank test. **E.** Genomic context of LINC01419. A CpG methylation site was found upstream of the LINC01419 coding sequence.

Next, we evaluated the changes in expression of the 7 lncRNAs during the five stages of HCC (healthy liver, cirrhosis, dysplasia, early HCC and advanced HCC) using one-way ANOVA (paired with an F test). We noted that LINC01419 was characterized by a significant increase in transcript expression from dysplasia to early HCC (Fig. 2A). The lncRNA AK021443 was also up-regulated in advanced HCC samples when compared with early HCC (Fig. 2B). Moreover, expression of LINC01419 and AK021443 was up-regulated in HCC tissues when compared with non-tumor liver tissue. AF070632 expression was down-regulated in HCC and was decreased in advanced HCC when compared with early HCC (Fig. 2C). These results suggest that LINC01419 may be related to the initiation of HCC, whereas AK021443 and AF070632 may be associated with the progression of HCC.

Because early detection biomarkers are critical for effective HCC care, we chose to further validate LINC01419, whose expression increased sharply from dysplasia to early HCC. We analyzed LINC01419 expression using quantitative real-time polymerase chain reaction (qRT-PCR) in 15 pairs of HCV-related HCC samples, 55 pairs of HBV-related HCC samples and their corresponding adjacent non-tumor liver tissues (Fig. 2D). These results confirmed that LINC01419 was overexpressed in HCV-related HCC and HBV-related HCC. We noted that LINC01419 was poorly expressed in adjacent normal liver tissue. The Ct values were greater than 30 in all adjacent normal liver tissues (data not shown). In addition, using the ENCODE database, we identified a CpG methylation site 113bp upstream of the transcription start site for LINC01419 in all six cell lines (HepG2, GM12878, H1-hESC, K562, Hela-S3, and HUVEC; Fig. 2E). Because DNA methylation of a promoter can repress the expression of the gene under the control of that promoter [30], this finding may partially explain the low level of LINC01419 expression. However, these findings should be confirmed experimentally.

### Prediction of lncRNA function

To investigate the role of lncRNAs in HCC pathogenesis, we used the gene expression profiles of 75 patient samples to construct a coding-non-coding co-expression network. The resulting network includes 220 lncRNA genes and 3,924 coding genes with 105,508 coding-coding edges, 7,114 noncoding-coding edges and 324 noncoding-noncoding edges (online resource). Next, we predicted the functions of the lncRNAs using two different methods, a module-based analysis and a hub-based analysis. First, the co-expression network was parsed into forty module-based subnetworks using an MCL algorithm. Twenty of these modules had at least one enriched GO term. Next, we obtained 154 lncRNA-centered subnetworks with at least one enriched GO term by parsing the co-expression network into different hub-based subnetworks.

**Figure 3:**
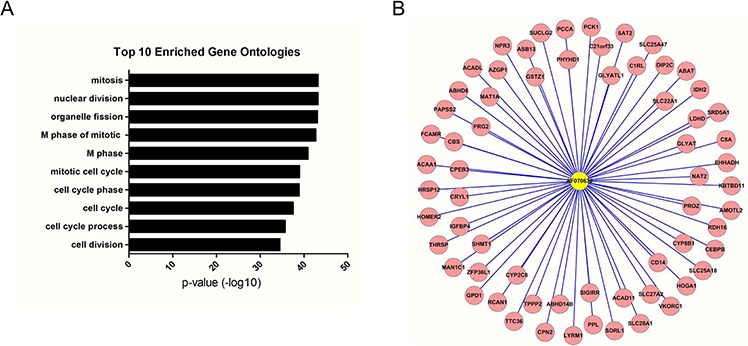
LncRNA functional prediction **A.** Functional enrichment result of the module containing LINC01419 and AK021443. **B.** The AF070632-centered subnetwork. Genes colored in red are protein-coding genes.

The functions of the 3 deregulated lncRNAs associated with the initiation or progression of HCC were predicted using the module-based method or the hub-based method. Our results suggest that LINC01419 and AK021443 were classified into the same module with the other 36 lncRNAs and 637 protein-coding genes. Because the co-expressed module could be regarded as a functional unit, LINC01419 and AK021443 may have the similar function with those 637 protein coding genes. Thus, by performing Go analysis on those protein-coding genes, we conclude that the functions of LINC01419 and AK021443 were mostly involved with cell cycle progression (Fig. 3A, Supplementary Table 3). The function of AF070632 was predicted using hub-based analysis. We found that it was surrounded by 64 differentially expressed protein-coding genes (Fig. 3B). Consistence with enriched function of these 64 protein-coding genes, AF070632 is mainly related to oxidation-reduction, cofactor binding and carboxylic acid catabolic process (Supplementary Table 4).

## DISCUSSION

In the present study, we reanalyzed a large microarray dataset to investigate the pathogenesis of HCV-related HCC. There are three main advantages of using this approach. First, with the exception often healthy liver tissue samples, the samples used in this microarray dataset were derived from patients with hepatitis C virus infection, which is the leading cause of HCC in the United States and Europe [4]. This study focused on lncRNA expression in HCV-induced HCC and identified lncRNAs that could be used as biomarkers for HCV-related HCC. Second, unlike some other studies comparing HCC tissues to adjacent normal tissues [28, 31], the samples in this microarray dataset were comprised of tissues representing each stage of HCV-related HCC [7], which provides better insight into the molecular basis underlying hepatocarcinogenesis. Finally, although RNA sequencing (RNA-seq) could be used to comprehensively identify lncRNA expression, the number of publically available RNA-seq databases and the application of RNA-seq for large-scale studies are limited by its cost. In contrast, there are many publically available microarray data sets for various types of cancer and clinical experiments. Because publically available microarray data contain probes that potentially match lncRNAs, we were able to repurpose these datasets to analyze lncRNA expression after reannotating the microarray probes. This approach has been used in other studies [32]. Moreover, the increased detection sensitivity of microarrays can facilitate the investigation of low-abundance lncRNAs.

Increasing evidence has suggested that lncRNAs serve critical functions [14]. Several studies have reported that lncRNAs are differentially expressed in different stages of HBV-related HCC, which suggests that lncRNAs may participate in the hepatocarcinogenic process. For example, lncRNA-HEIH is overexpressed in HCC tissue when compared with adjacent normal tissue, suggesting that this lncRNA could promote tumor growth. Notably, lncRNA-HEIH expression was also up-regulated in cirrhotic liver tissue when compared with healthy liver tissue [27]. Furthermore, lncRNA-ATB, another lncRNA, is overexpressed in HCC tissues when compared with adjacent normal tissues. lncRNA-ATB expression is negatively correlated with prognosis in HCC patients. Interestingly, the expression of lncRNA-ATB was up-regulated in portal vein tumor thrombi when compared with HCC tissues [33]. In the current study, we describe the lncRNA signatures of the multiple stages of HCV-related HCC and identify a set of lncRNAs that could play a key role in hepatocarcinogenesis. We also identify 7 lncRNAs that were deregulated between preneoplastic lesions and HCC. Further analysis showed that 3 of the 7 lncRNAs were differentially expressed between dysplasia, early HCC, and advanced HCC. Our results suggest that these 3 lncRNAs are associated with the initiation or progression of HCC and may serve as diagnostic or prognostic biomarkers for HCC. Among the 3 deregulated lncRNAs, the up-regulated expression of LINC01419 in HCC was confirmed by quantitative real-time polymerase chain reaction (qRT-PCR). Interestingly, LINC01419 is poorly expressed in adjacent normal liver tissue, suggesting that LINC01419could serve as a specific biomarker for the detection of HCC.

The cell cycle controls cell division, which is critical to cancer. Cyclin-dependent kinases (CDKs), which are key regulators of the cell cycle, have been investigated as potential therapeutic targets for cancer [34]. Cell cycle deregulation is also common in HCC [35]. For example, a recent study showed that cell cycle-related kinase, a member of the latest cyclin-dependent kinase, could facilitate the development of HCC by activating β-catenin/T cell factor signaling [36]. TTK is a critical mitotic checkpoint protein and participates in the p53 signaling pathway. The expression of TTK in HCC tissues is associated with the Edmonson tumor grade, recurrence-free survival and overall survival [37]. Moreover, SKP2 and CKS1 in HCC tissues could promote G1-S transition and active DNA synthesis by degrading cell cycle inhibitors [38, 39]. However, the regulatory role of lncRNAs in the cell cycle is poorly understood. In this study, using gene co-expression network analysis, we found that LINC01419 and AK021443 were clustered into one functional module for cell cycle regulation. In addition, these two lncRNAs were over expressed in HCC tissues. Thus, our findings suggest that LINC01419 and AK021443 could regulate cell cycle activity and thereby promote tumor growth.

Taken together, our results provide a comprehensive understanding of lncRNAs in HCV-related HCC and a basis for identifying specific biomarkers or therapeutic targets for HCC.

## MATERIALS AND METHODS

### Microarray data information and computational process

The microarray data used in this study were generated using the Affymetrix platform HG-U133A Plus 2.0. We downloaded these publicly available data sets from the Gene Expression Omnibus (GEO) database (accession number: GSE6764). The microarray data assessed the gene expression profiles of 75 samples consisting of 10 normal liver tissue samples, 13 cirrhotic liver tissue samples, 17 dysplastic nodules, and 35 HCCs. The preneoplastic lesions (cirrhosis and dysplasia) and HCC samples were obtained from 38 patients infected with HCV. Patients with other etiologies, such as HBV infection, were excluded from our analysis.35 HCC samples were categorized using the four following criteria: (i) according to tumor size, where well-differentiated tumors <2 cm in diameter with no vascular invasion and satellites were categorized as very early stage HCC (8 cases); (ii) according to tumor differentiation, where microscopic tumors measuring <2 cm in diameter, well-to moderately differentiated tumors measuring 2–5 cm in diameter without vascular invasion or satellites and tumors with 2–3 well-differentiated nodules measuring <3 cm in diameter were categorized as early stage HCC (10 cases); (iii) according to tumor number, where poorly differentiated tumors measuring >2 cm in diameter with microvascular invasion and satellites or tumors measuring >5 cm in diameter were categorized as advanced stage HCC (7 cases); (iv) according to vascular invasion and satellites, where tumors with macrovascular invasion or diffuse liver involvement were categorized as very advanced stage HCC (10 cases) [7].

### Patients

Sixty-five frozen HCC tissues and matched adjacent normal liver tissues were randomly obtained from sixty-five patients who underwent hepatectomy at Peking Union Medical College Hospital (Beijing, China). This patient cohort consisted of fifty-five patients who were infected with HBV and fifteen patients who were infected with HCV. We used these samples to perform quantitative real-time polymerase chain reaction (qRT-PCR) analysis.

### Repurposing microarray data to analyze the expression of protein-coding genes and lncRNA genes

Non-coding RNA Function Annotation Server (ncFANs) can be used to quantify the levels of messenger RNAs and long non-coding RNAs depending on the re-annotation of Affymetrix microarray probes [40]. We submitted microarray data (in CEL format) to ncFANs and obtained both protein-coding and lncRNA gene expression values (log2-transformed) for 73 samples using R (2 samples were eliminated for quality control reasons). The Robust Multichip Average (RMA) analysis, Student's *t*-test, one-way ANOVA (paired with an F test) and Benjamini Hochberg (BH) FDR correction were performed using the Limma statistical package. Genes with a fold-change >2 and BH FDR-adjusted *p*-values < 0.05 were considered differentially expressed.

### PCR analysis

Total RNA was isolated using TRIzol reagent (Invitrogen). First-strand cDNA was synthesized using a High Capacity cDNA Reverse Transcription kit (Applied Biosystems) according to instructions suggested by the manufacturer. Real-time PCR was performed using Power SYBR^®^ Green Master mix (Applied Biosystems) and a StepOne™ Real-Time PCR System (Applied Biosystems, Foster City, USA). The relative expression levels of the lncRNAs were calculated using the comparative Ct method. GAPDH gene expression was included as an internal control. The primers for LINC01419were (forward) 5′-GAAACTCCGAACACATCTG-3′ and (reverse) 5′-TTCTCCTGCTGGTTGATT-3′; for GAPDH, (forward) 5′-TCAAGGCTGAGAACGGGAAG-3′ and (reverse) 5′-GTGAAGACGCCAGTGGACT-3′.

### Functional enrichment of protein-coding genes

The DAVID Bioinformatics Tool [41] was used to identify functional enrichment of target protein-coding genes. This tool can be used to perform gene ontology analysis to identify biological processes related to protein-coding genes. For this analysis, we used a *p*-value significance threshold of < 0.01. Finally, we used the Enrichment Map plugin [42] for Cytoscape [43] to visualize the biological process organization.

### Functional analysis of lncRNAs

Gene expression data for 73 tissue samples was submitted to ncFANs to generate a coding-non-coding co-expression network. To build the co-expression network, we chose genes (both protein-coding genes and lncRNA genes) with expression differences that ranked in the top 75^th^ percentile. A Pearson's correlation coefficient or a Spearman Rank correlation coefficient was used to estimate the relationship of each gene pair, including coding-coding, coding-lncRNA and lncRNA-lncRNA gene pairs. The *p*-values of the correlation coefficients for all gene pairs were calculated using Fisher's asymptotic test and then were adjusted using Bonferronimultiple testing correction. We chose co-expression gene pairs with *p*-values < 0.01 to construct the coding-non-coding co-expression network. Based on the coding-non-coding co-expression network, we used module-based and hub-based methods embedded in ncFANs to predict the function of the lncRNAs. In the module based method, modules of co-expressed genes in the coding-non-coding co-expression network were identified using the Markov cluster algorithm (MCL). The protein coding genes in the same module as the lncRNAs were further analyzed using gene ontology analysis to predict the functions of the lncRNAs in this module. In the hub-based method, the co-expression network was parsed to identify subnetworks comprising a central lncRNA and its neighboring protein-coding genes. The functional enrichment of these connected protein-coding genes was considered the function of the corresponding lncRNA. Only function enrichments with *p*-values < 0.01 were retained.

## SUPPLEMENTARY TABLES












