# Supplementary material for: Long non-coding RNA expression profiles of hepatitis C virus-related dysplasia and hepatocellular carcinoma
Source: Oncotarget. 2015 Oct 26;6(41):43770–8. doi: 10.18632/oncotarget.6087 (PMC4791265; doi:10.18632/oncotarget.6087)
Supplement: Supplementary file 1 [file oncotarget-06-43770-s001.pdf]

SUPPLYMENTRY TABLES

Supplementary File S1: The list of differentially expressed genes between preneoplasticlesions and HCC.

Supplementary Table S1: Functional enrichment result of up-regulated protein coding gene in HCC samples

Supplementary Table S2: Functional enrichment result of down-regulated protein coding gene in HCC samples

Supplementary Table S3: Functional enrichment result of the module containing LINC01419 and AK021443

Supplementary Table S4: Functional enrichment result of the AF070632-centered subnetwork
