# Supplementary material for: Long non-coding RNA expression profiles of hepatitis C virus-related dysplasia and hepatocellular carcinoma
Source: Oncotarget. 2015 Oct 26;6(41):43770–8. doi: 10.18632/oncotarget.6087 (PMC4791265; doi:10.18632/oncotarget.6087)
Supplement: Supplementary file 2 [file oncotarget-06-43770-s002.docx]

| Gene ID | logFC | AveExpr | t | P.Value | adj.P.Value | B |
| --- | --- | --- | --- | --- | --- | --- |
| 10 | -3.011788 | 8.1136934 | -9.87922 | 1.89E-14 | 8.60E-12 | 22.60088 |
| 33 | -1.098668 | 6.5421755 | -4.29513 | 6.12E-05 | 0.000393484 | 1.323084 |
| 123 | -1.033363 | 11.031172 | -5.48912 | 7.58E-07 | 1.00E-05 | 5.54417 |
| 124 | -1.128425 | 12.175509 | -3.98958 | 0.00017425 | 0.000958277 | 0.327753 |
| 126 | -1.567662 | 11.441125 | -3.65422 | 0.00052537 | 0.002437242 | -0.71362 |
| 127 | -1.611867 | 8.2206254 | -5.72274 | 3.08E-07 | 4.79E-06 | 6.416698 |
| 173 | -2.219167 | 10.440254 | -5.74493 | 2.83E-07 | 4.46E-06 | 6.500156 |
| 174 | 2.3428526 | 7.2521457 | 3.964312 | 0.00018969 | 0.001030433 | 0.247352 |
| 185 | -1.104124 | 9.2649015 | -7.48376 | 2.77E-10 | 1.64E-08 | 13.24284 |
| 189 | -1.508601 | 11.545542 | -5.61954 | 4.59E-07 | 6.67E-06 | 6.02981 |
| 218 | 2.2209106 | 5.7634834 | 4.391885 | 4.36E-05 | 0.000296559 | 1.646671 |
| 219 | -1.192977 | 8.1736571 | -8.12255 | 2.10E-11 | 1.99E-09 | 15.76104 |
| 229 | -1.355001 | 9.9290663 | -6.28596 | 3.38E-08 | 7.86E-07 | 8.5622 |
| 306 | -1.314717 | 4.4763507 | -6.63894 | 8.29E-09 | 2.50E-07 | 9.929623 |
| 319 | -2.580419 | 8.5829966 | -6.9503 | 2.38E-09 | 9.34E-08 | 11.14583 |
| 355 | -1.34386 | 7.6068535 | -5.6466 | 4.14E-07 | 6.12E-06 | 6.131056 |
| 390 | -1.541305 | 9.8948716 | -8.99576 | 6.27E-13 | 1.41E-10 | 19.18629 |
| 435 | -1.048241 | 10.556582 | -5.64502 | 4.16E-07 | 6.15E-06 | 6.125128 |
| 443 | -1.005851 | 5.5252218 | -5.37993 | 1.15E-06 | 1.41E-05 | 5.140597 |
| 445 | -1.098987 | 12.311816 | -6.83802 | 3.73E-09 | 1.34E-07 | 10.70637 |
| 467 | -1.143415 | 7.419006 | -5.15687 | 2.68E-06 | 2.85E-05 | 4.325537 |
| 563 | -1.561616 | 11.804417 | -6.88175 | 3.13E-09 | 1.17E-07 | 10.87741 |
| 590 | -1.331446 | 8.5785014 | -3.33862 | 0.00141358 | 0.00557021 | -1.63889 |
| 635 | -1.698687 | 10.423112 | -6.92243 | 2.66E-09 | 1.02E-07 | 11.03665 |
| 701 | 1.7286845 | 5.0550502 | 7.055233 | 1.56E-09 | 6.54E-08 | 11.55726 |
| 713 | -1.018721 | 10.266054 | -5.22542 | 2.07E-06 | 2.28E-05 | 4.574598 |
| 729 | -1.596927 | 11.057211 | -5.67262 | 3.74E-07 | 5.64E-06 | 6.228535 |
| 730 | -2.719919 | 8.8296269 | -6.77027 | 4.90E-09 | 1.64E-07 | 10.44166 |
| 731 | -1.448634 | 10.282636 | -6.79355 | 4.46E-09 | 1.52E-07 | 10.53256 |
| 735 | -2.1575 | 10.873645 | -4.90235 | 6.90E-06 | 6.25E-05 | 3.41264 |
| 760 | -1.792618 | 9.1432444 | -7.12374 | 1.18E-09 | 5.20E-08 | 11.8262 |
| 890 | 1.5759855 | 4.7942765 | 6.775648 | 4.80E-09 | 1.61E-07 | 10.46265 |
| 891 | 1.8469509 | 4.8200312 | 7.194006 | 8.92E-10 | 4.10E-08 | 12.10232 |
| 912 | -1.417998 | 5.9053039 | -6.51232 | 1.37E-08 | 3.74E-07 | 9.437492 |
| 922 | -1.447831 | 7.626283 | -6.38655 | 2.27E-08 | 5.62E-07 | 8.950397 |
| 929 | -1.394981 | 10.818394 | -6.77906 | 4.73E-09 | 1.60E-07 | 10.47597 |
| 969 | -1.081288 | 6.0828354 | -2.95332 | 0.00440659 | 0.014220492 | -2.6879 |
| 978 | -1.095001 | 7.0684837 | -5.41562 | 1.00E-06 | 1.26E-05 | 5.272201 |
| 983 | 1.4519584 | 5.1342587 | 6.735661 | 5.63E-09 | 1.84E-07 | 10.30657 |
| 990 | 1.0002676 | 3.9364302 | 4.932783 | 6.17E-06 | 5.70E-05 | 3.520758 |
| 991 | 1.8693562 | 5.2233888 | 6.973572 | 2.17E-09 | 8.61E-08 | 11.23701 |
| 999 | -1.262384 | 8.1126308 | -7.40252 | 3.84E-10 | 2.11E-08 | 12.92283 |
| 1012 | 1.4336543 | 4.9250131 | 6.968584 | 2.21E-09 | 8.71E-08 | 11.21746 |
| 1026 | -1.123496 | 8.9739816 | -6.66952 | 7.34E-09 | 2.28E-07 | 10.0487 |
| 1033 | 2.1733415 | 5.4514027 | 8.810018 | 1.32E-12 | 2.41E-10 | 18.46089 |
| 1047 | 1.3917145 | 4.5613013 | 4.882672 | 7.42E-06 | 6.63E-05 | 3.342886 |
| 1063 | 1.4013641 | 5.058984 | 7.436105 | 3.36E-10 | 1.92E-08 | 13.05511 |
| 1071 | -2.279098 | 6.3854506 | -10.2519 | 4.41E-15 | 3.22E-12 | 24.01992 |
| 1080 | -1.252201 | 5.1833113 | -6.37688 | 2.36E-08 | 5.79E-07 | 8.913043 |
| 1116 | -1.789173 | 10.207656 | -3.88245 | 0.00024927 | 0.001300211 | -0.01103 |
| 1306 | 2.0164062 | 6.0410469 | 6.280786 | 3.45E-08 | 7.98E-07 | 8.542262 |
| 1356 | -1.100612 | 11.393063 | -6.428 | 1.92E-08 | 4.90E-07 | 9.110733 |
| 1373 | -1.176796 | 12.026639 | -3.62481 | 0.00057734 | 0.002632396 | -0.8022 |
| 1393 | -4.269906 | 7.2929074 | -12.2817 | 2.06E-18 | 3.75E-15 | 31.46431 |
| 1401 | -1.967509 | 10.36279 | -3.46666 | 0.00095181 | 0.004003466 | -1.2703 |
| 1409 | -1.554121 | 7.2298552 | -5.90553 | 1.51E-07 | 2.68E-06 | 7.10711 |
| 1491 | -1.763454 | 9.2439602 | -5.4052 | 1.05E-06 | 1.30E-05 | 5.23374 |
| 1544 | -3.193871 | 7.955058 | -9.76559 | 2.96E-14 | 1.20E-11 | 22.16548 |
| 1548 | -2.045504 | 10.230122 | -6.69787 | 6.55E-09 | 2.09E-07 | 10.1592 |
| 1549 | -1.834245 | 6.4454442 | -6.15845 | 5.60E-08 | 1.19E-06 | 8.072017 |
| 1551 | -1.476943 | 10.713442 | -3.51804 | 0.00081023 | 0.003499297 | -1.11976 |
| 1555 | -2.510799 | 8.6483757 | -8.77739 | 1.50E-12 | 2.70E-10 | 18.33326 |
| 1556 | -2.238347 | 7.6086865 | -6.61752 | 9.03E-09 | 2.66E-07 | 9.846252 |
| 1558 | -2.016096 | 9.7790259 | -6.64049 | 8.24E-09 | 2.49E-07 | 9.935635 |
| 1559 | -1.690448 | 11.586776 | -6.15157 | 5.75E-08 | 1.22E-06 | 8.045655 |
| 1562 | -1.622501 | 8.6783932 | -4.9524 | 5.74E-06 | 5.36E-05 | 3.590604 |
| 1571 | -1.16983 | 12.450767 | -3.34074 | 0.00140445 | 0.00553744 | -1.63287 |
| 1576 | -1.262742 | 10.42168 | -2.95836 | 0.00434391 | 0.014064783 | -2.6748 |
| 1579 | -1.326059 | 7.5610757 | -6.07337 | 7.82E-08 | 1.57E-06 | 7.746284 |
| 1581 | 3.0232605 | 5.9727593 | 5.445272 | 8.97E-07 | 1.15E-05 | 5.381758 |
| 1582 | -2.526657 | 10.49467 | -6.77662 | 4.78E-09 | 1.61E-07 | 10.46646 |
| 1592 | -1.273568 | 6.3260511 | -5.00658 | 4.69E-06 | 4.53E-05 | 3.78415 |
| 1610 | -1.30616 | 8.1735991 | -5.95281 | 1.26E-07 | 2.30E-06 | 7.286703 |
| 1634 | -2.585297 | 9.677208 | -7.2905 | 6.04E-10 | 3.04E-08 | 12.48183 |
| 1672 | -2.108182 | 9.67585 | -5.77014 | 2.56E-07 | 4.11E-06 | 6.5951 |
| 1723 | -1.052689 | 7.6994563 | -4.86729 | 7.86E-06 | 6.94E-05 | 3.288451 |
| 1728 | 1.9783986 | 6.8721629 | 4.099919 | 0.0001199 | 0.000701633 | 0.682358 |
| 1733 | -1.309945 | 10.224414 | -4.43813 | 3.70E-05 | 0.000257627 | 1.802653 |
| 1776 | -2.508384 | 9.3462839 | -10.9428 | 3.07E-16 | 2.86E-13 | 26.61037 |
| 1805 | -1.750021 | 6.2990298 | -5.65344 | 4.03E-07 | 6.00E-06 | 6.156654 |
| 1827 | -1.721961 | 7.8938602 | -9.32237 | 1.71E-13 | 5.14E-11 | 20.45604 |
| 1843 | -1.116081 | 8.2880145 | -6.5064 | 1.41E-08 | 3.81E-07 | 9.414516 |
| 1847 | -1.326011 | 7.6784271 | -5.81073 | 2.19E-07 | 3.59E-06 | 6.748264 |
| 1893 | -1.50709 | 7.2636165 | -12.4707 | 1.04E-18 | 2.35E-15 | 32.13045 |
| 1910 | -1.302476 | 7.4056323 | -8.12927 | 2.04E-11 | 1.96E-09 | 15.78752 |
| 1945 | 1.1542203 | 6.1578343 | 9.145662 | 3.45E-13 | 9.04E-11 | 19.77002 |
| 1958 | -1.318198 | 8.5108536 | -5.41756 | 9.97E-07 | 1.26E-05 | 5.279368 |
| 1959 | -1.463688 | 6.3672985 | -4.90036 | 6.96E-06 | 6.29E-05 | 3.405561 |
| 1969 | -1.266543 | 6.9063814 | -7.69885 | 1.16E-10 | 7.87E-09 | 14.09076 |
| 2027 | -1.980361 | 7.2098874 | -6.78561 | 4.61E-09 | 1.56E-07 | 10.50154 |
| 2053 | -1.311299 | 8.9245991 | -7.11633 | 1.22E-09 | 5.33E-08 | 11.7971 |
| 2110 | -1.132705 | 8.7186351 | -6.84931 | 3.57E-09 | 1.29E-07 | 10.75051 |
| 2114 | -1.226128 | 9.1683798 | -8.05336 | 2.77E-11 | 2.46E-09 | 15.48845 |
| 2146 | 1.4103213 | 5.5512491 | 7.667587 | 1.32E-10 | 8.70E-09 | 13.96748 |
| 2152 | -1.090378 | 6.0163254 | -3.97836 | 0.00018095 | 0.000989276 | 0.292008 |
| 2158 | -2.004963 | 11.376371 | -5.10877 | 3.21E-06 | 3.31E-05 | 4.15156 |
| 2160 | -1.086393 | 8.5277166 | -5.71291 | 3.20E-07 | 4.93E-06 | 6.379757 |
| 2180 | -1.231239 | 11.319257 | -7.27262 | 6.49E-10 | 3.22E-08 | 12.41147 |
| 2203 | -2.210278 | 10.224847 | -7.54793 | 2.14E-10 | 1.33E-08 | 13.49575 |
| 2213 | -2.431104 | 6.6704976 | -8.05228 | 2.79E-11 | 2.46E-09 | 15.48417 |
| 2215 | -1.253978 | 6.0259765 | -5.2976 | 1.57E-06 | 1.83E-05 | 4.838246 |
| 2220 | -3.894607 | 6.9921109 | -13.1445 | 9.22E-20 | 5.09E-16 | 34.46592 |
| 2258 | 1.3801455 | 6.0008965 | 4.979335 | 5.19E-06 | 4.92E-05 | 3.686704 |
| 2263 | -1.530658 | 6.8014794 | -6.32931 | 2.85E-08 | 6.79E-07 | 8.729326 |
| 2274 | -1.06085 | 7.2528349 | -4.31413 | 5.72E-05 | 0.000371764 | 1.386327 |
| 2353 | -1.916646 | 8.3362435 | -6.40585 | 2.10E-08 | 5.27E-07 | 9.025031 |
| 2357 | -1.386188 | 6.6662259 | -7.5121 | 2.47E-10 | 1.48E-08 | 13.35452 |
| 2534 | -1.005521 | 6.9897322 | -6.81872 | 4.03E-09 | 1.41E-07 | 10.63093 |
| 2539 | 1.0746751 | 5.8175633 | 4.970666 | 5.36E-06 | 5.05E-05 | 3.655748 |
| 2564 | 1.005573 | 6.5288073 | 3.649649 | 0.00053315 | 0.002466613 | -0.72743 |
| 2643 | -1.30825 | 9.7359565 | -6.32836 | 2.86E-08 | 6.80E-07 | 8.725671 |
| 2669 | -1.144951 | 7.6416285 | -3.35631 | 0.00133908 | 0.005317602 | -1.58853 |
| 2690 | -2.307486 | 9.4502539 | -7.21493 | 8.20E-10 | 3.81E-08 | 12.18457 |
| 2706 | -1.021756 | 8.3467945 | -5.19313 | 2.34E-06 | 2.52E-05 | 4.457139 |
| 2719 | 3.768764 | 8.6573398 | 7.463487 | 3.01E-10 | 1.75E-08 | 13.16298 |
| 2731 | -1.104339 | 9.6600253 | -4.2499 | 7.16E-05 | 0.000449251 | 1.173166 |
| 2752 | 1.4792 | 9.8581341 | 5.359937 | 1.24E-06 | 1.50E-05 | 5.067018 |
| 2805 | -1.259248 | 10.901183 | -5.42199 | 9.80E-07 | 1.24E-05 | 5.295718 |
| 2819 | -1.226026 | 7.2222069 | -8.49368 | 4.70E-12 | 6.54E-10 | 17.221 |
| 2823 | -1.275231 | 5.3112931 | -7.27137 | 6.53E-10 | 3.23E-08 | 12.40656 |
| 2920 | -1.248336 | 9.8538397 | -4.91912 | 6.49E-06 | 5.95E-05 | 3.472187 |
| 2938 | -1.050933 | 12.473465 | -3.82803 | 0.00029841 | 0.001508855 | -0.18098 |
| 2954 | -1.543391 | 8.5434373 | -8.23311 | 1.34E-11 | 1.43E-09 | 16.19642 |
| 2998 | -2.482147 | 8.9638588 | -6.58205 | 1.04E-08 | 2.98E-07 | 9.708295 |
| 3026 | -1.749415 | 9.861354 | -6.58454 | 1.03E-08 | 2.96E-07 | 9.717989 |
| 3034 | -1.800229 | 9.1158216 | -4.36838 | 4.73E-05 | 0.000317476 | 1.567723 |
| 3040 | -1.602818 | 10.631847 | -6.251 | 3.88E-08 | 8.85E-07 | 8.427591 |
| 3043 | -1.755531 | 10.692902 | -6.31185 | 3.05E-08 | 7.20E-07 | 8.661974 |
| 3083 | -1.674555 | 7.3898841 | -5.0467 | 4.04E-06 | 4.02E-05 | 3.92801 |
| 3117 | -1.659438 | 7.6122314 | -2.67426 | 0.00951622 | 0.026789317 | -3.38672 |
| 3119 | -1.199617 | 6.9854284 | -2.70239 | 0.00882471 | 0.025205124 | -3.31875 |
| 3148 | 1.1305223 | 8.2628609 | 5.730904 | 2.98E-07 | 4.68E-06 | 6.447391 |
| 3161 | 1.2544157 | 4.6417082 | 7.155482 | 1.04E-09 | 4.70E-08 | 11.95091 |
| 3162 | -1.311907 | 9.1231767 | -6.84315 | 3.66E-09 | 1.32E-07 | 10.72641 |
| 3242 | -1.427734 | 12.17576 | -3.99535 | 0.0001709 | 0.000943128 | 0.346167 |
| 3248 | -1.512115 | 8.7255756 | -3.35702 | 0.00133617 | 0.005310181 | -1.58651 |
| 3273 | -1.126077 | 12.380549 | -3.72263 | 0.00042121 | 0.00202045 | -0.50584 |
| 3290 | -1.651467 | 11.109687 | -3.42008 | 0.0011002 | 0.004525308 | -1.40551 |
| 3294 | -1.577516 | 10.34153 | -7.63696 | 1.49E-10 | 9.60E-09 | 13.8467 |
| 3397 | -1.273393 | 9.1654429 | -4.64024 | 1.80E-05 | 0.000140175 | 2.494124 |
| 3398 | -1.091876 | 10.622483 | -7.86964 | 5.82E-11 | 4.55E-09 | 14.76421 |
| 3479 | -1.922017 | 8.5511536 | -6.18629 | 5.01E-08 | 1.09E-06 | 8.178864 |
| 3481 | -1.725702 | 7.228899 | -4.26501 | 6.79E-05 | 0.000429981 | 1.223144 |
| 3486 | -1.707276 | 10.167633 | -8.00493 | 3.37E-11 | 2.89E-09 | 15.29757 |
| 3491 | -1.705176 | 8.3128663 | -6.82921 | 3.87E-09 | 1.38E-07 | 10.67194 |
| 3512 | -2.426263 | 8.8599691 | -4.9228 | 6.40E-06 | 5.89E-05 | 3.485271 |
| 3556 | -1.115191 | 7.604599 | -5.36302 | 1.23E-06 | 1.49E-05 | 5.078351 |
| 3598 | -2.152027 | 5.1015989 | -7.567 | 1.98E-10 | 1.24E-08 | 13.57091 |
| 3626 | -1.254291 | 6.531462 | -7.06058 | 1.53E-09 | 6.45E-08 | 11.57823 |
| 3700 | -1.116295 | 8.830805 | -5.38181 | 1.14E-06 | 1.40E-05 | 5.147519 |
| 3726 | -1.301549 | 8.2982598 | -7.61511 | 1.63E-10 | 1.04E-08 | 13.76057 |
| 3818 | -1.324198 | 9.4952523 | -5.77212 | 2.54E-07 | 4.09E-06 | 6.602588 |
| 3832 | 1.1632084 | 3.8741129 | 5.331112 | 1.39E-06 | 1.64E-05 | 4.96111 |
| 3931 | -1.017359 | 6.305824 | -8.13689 | 1.98E-11 | 1.94E-09 | 15.81753 |
| 3934 | 1.8303004 | 8.2824353 | 4.106582 | 0.00011721 | 0.000687796 | 0.70395 |
| 3950 | -1.265249 | 11.190311 | -3.74483 | 0.00039189 | 0.00190163 | -0.43791 |
| 3953 | -1.366759 | 9.1709539 | -5.41647 | 1.00E-06 | 1.26E-05 | 5.275315 |
| 3958 | -1.114773 | 9.9458976 | -4.1637 | 9.64E-05 | 0.000581813 | 0.889855 |
| 3977 | -1.560149 | 6.0188457 | -9.76345 | 2.98E-14 | 1.20E-11 | 22.15726 |
| 4018 | -1.574466 | 6.8004483 | -5.94441 | 1.30E-07 | 2.35E-06 | 7.25476 |
| 4060 | -2.237199 | 8.8847391 | -4.53295 | 2.64E-05 | 0.000194404 | 2.125152 |
| 4061 | -2.22926 | 9.0168234 | -8.78827 | 1.44E-12 | 2.61E-10 | 18.37582 |
| 4072 | -3.223342 | 6.1821032 | -6.79456 | 4.45E-09 | 1.52E-07 | 10.5365 |
| 4085 | 1.7109053 | 5.4625756 | 6.319433 | 2.96E-08 | 7.01E-07 | 8.69123 |
| 4100 | 1.0385291 | 3.9653294 | 4.018878 | 0.00015787 | 0.000882866 | 0.421361 |
| 4133 | 1.7045956 | 6.5459747 | 7.222359 | 7.95E-10 | 3.73E-08 | 12.21379 |
| 4153 | -1.704988 | 10.736945 | -5.09181 | 3.42E-06 | 3.50E-05 | 4.090364 |
| 4163 | -1.258127 | 6.781951 | -6.94627 | 2.42E-09 | 9.44E-08 | 11.13002 |
| 4192 | 1.5211149 | 7.7202467 | 5.653751 | 4.02E-07 | 6.00E-06 | 6.157815 |
| 4224 | 1.2354662 | 4.4573695 | 3.280686 | 0.00168588 | 0.006417081 | -1.80251 |
| 4239 | -1.276868 | 6.2879492 | -5.87907 | 1.68E-07 | 2.91E-06 | 7.006768 |
| 4316 | -1.062035 | 7.1447794 | -2.77291 | 0.00728806 | 0.021532727 | -3.14586 |
| 4321 | 1.6137354 | 4.82049 | 3.109352 | 0.00280845 | 0.009790918 | -2.27438 |
| 4436 | 1.3751888 | 6.0133808 | 8.487779 | 4.81E-12 | 6.61E-10 | 17.19785 |
| 4482 | -1.035722 | 8.0578411 | -7.40017 | 3.88E-10 | 2.12E-08 | 12.91357 |
| 4493 | -1.612307 | 11.373341 | -4.60656 | 2.03E-05 | 0.000154772 | 2.377855 |
| 4494 | -1.599653 | 8.7549559 | -5.78244 | 2.44E-07 | 3.94E-06 | 6.641495 |
| 4495 | -1.695814 | 9.7370243 | -5.67165 | 3.75E-07 | 5.65E-06 | 6.22487 |
| 4499 | -2.480656 | 7.4948504 | -5.70567 | 3.29E-07 | 5.06E-06 | 6.352557 |
| 4501 | -1.654192 | 10.60122 | -5.29335 | 1.60E-06 | 1.85E-05 | 4.822704 |
| 4502 | -1.12575 | 12.629861 | -6.02544 | 9.45E-08 | 1.82E-06 | 7.563275 |
| 4548 | 1.0188294 | 7.3586081 | 8.143138 | 1.93E-11 | 1.91E-09 | 15.84215 |
| 4616 | -1.374929 | 8.4960325 | -7.11548 | 1.22E-09 | 5.34E-08 | 11.79378 |
| 4837 | -1.757984 | 11.721006 | -5.39057 | 1.11E-06 | 1.36E-05 | 5.179783 |
| 4869 | 1.2684003 | 9.6824536 | 9.071114 | 4.64E-13 | 1.13E-10 | 19.47991 |
| 4886 | -2.305153 | 6.1676112 | -8.03125 | 3.03E-11 | 2.64E-09 | 15.40132 |
| 4929 | -1.476155 | 6.574156 | -4.814 | 9.55E-06 | 8.17E-05 | 3.100448 |
| 5009 | -1.214338 | 10.06414 | -4.56487 | 2.36E-05 | 0.000176316 | 2.234472 |
| 5042 | 1.0702611 | 10.081817 | 8.444213 | 5.74E-12 | 7.29E-10 | 17.02671 |
| 5054 | -1.351072 | 8.4116108 | -4.78344 | 1.07E-05 | 9.02E-05 | 2.993058 |
| 5067 | -2.710182 | 5.4436259 | -9.13995 | 3.53E-13 | 9.04E-11 | 19.74781 |
| 5068 | 1.3316063 | 4.3333621 | 3.718987 | 0.00042623 | 0.002040258 | -0.51698 |
| 5105 | -1.796306 | 11.95199 | -5.39652 | 1.08E-06 | 1.34E-05 | 5.201716 |
| 5142 | -1.112937 | 6.4007126 | -5.55603 | 5.86E-07 | 8.10E-06 | 5.79285 |
| 5156 | -2.746377 | 8.2532337 | -7.30033 | 5.81E-10 | 2.95E-08 | 12.5205 |
| 5168 | -1.565552 | 8.6858561 | -5.48345 | 7.75E-07 | 1.02E-05 | 5.523153 |
| 5199 | -1.378263 | 6.70441 | -12.4412 | 1.15E-18 | 2.36E-15 | 32.02664 |
| 5209 | -1.05116 | 7.825198 | -4.5759 | 2.26E-05 | 0.000170379 | 2.272343 |
| 5225 | 1.2504449 | 5.1097115 | 3.276826 | 0.00170568 | 0.006485184 | -1.81334 |
| 5244 | -1.280008 | 10.576209 | -5.00319 | 4.75E-06 | 4.57E-05 | 3.771999 |
| 5267 | -1.375598 | 10.012306 | -6.05318 | 8.47E-08 | 1.67E-06 | 7.669148 |
| 5274 | 1.0872145 | 5.896197 | 5.177851 | 2.47E-06 | 2.65E-05 | 4.401635 |
| 5320 | -1.787197 | 11.223429 | -3.40281 | 0.00116056 | 0.004732652 | -1.4553 |
| 5324 | 1.3022276 | 4.3675262 | 6.122271 | 6.45E-08 | 1.34E-06 | 7.933386 |
| 5348 | -2.231438 | 8.8087769 | -6.43245 | 1.89E-08 | 4.84E-07 | 9.127973 |
| 5359 | -1.065275 | 9.402005 | -6.54709 | 1.20E-08 | 3.34E-07 | 9.572464 |
| 5420 | 1.2208722 | 7.3497965 | 6.34156 | 2.71E-08 | 6.50E-07 | 8.776611 |
| 5444 | -1.515384 | 11.261238 | -4.11424 | 0.00011419 | 0.00067317 | 0.728782 |
| 5446 | -1.35463 | 11.208793 | -5.5672 | 5.62E-07 | 7.82E-06 | 5.834437 |
| 5498 | 1.0272247 | 6.6672863 | 10.57745 | 1.25E-15 | 1.06E-12 | 25.2473 |
| 5552 | -1.062664 | 9.8271266 | -4.8457 | 8.50E-06 | 7.42E-05 | 3.212166 |
| 5557 | 1.7073476 | 5.8532693 | 8.028269 | 3.07E-11 | 2.66E-09 | 15.38956 |
| 5577 | -1.175748 | 5.349745 | -5.95759 | 1.23E-07 | 2.26E-06 | 7.304843 |
| 5591 | 1.3905764 | 7.622648 | 7.679456 | 1.26E-10 | 8.37E-09 | 14.01427 |
| 5648 | -1.272489 | 6.8649609 | -11.3426 | 6.74E-17 | 8.12E-14 | 28.08379 |
| 5710 | 1.176163 | 9.7595311 | 10.14937 | 6.57E-15 | 4.34E-12 | 23.63089 |
| 5723 | 1.0792613 | 5.3127441 | 5.130589 | 2.95E-06 | 3.09E-05 | 4.230395 |
| 5743 | -1.139268 | 4.2275213 | -6.41555 | 2.02E-08 | 5.10E-07 | 9.062553 |
| 5858 | -1.069227 | 6.2628125 | -7.7072 | 1.12E-10 | 7.66E-09 | 14.12369 |
| 5929 | 1.0099069 | 6.1109818 | 8.655614 | 2.45E-12 | 3.89E-10 | 17.85635 |
| 5947 | -1.720422 | 8.376305 | -4.84826 | 8.43E-06 | 7.37E-05 | 3.221204 |
| 5959 | -1.122915 | 7.6106926 | -6.25898 | 3.76E-08 | 8.62E-07 | 8.458277 |
| 5984 | 1.1617199 | 6.7873859 | 6.692698 | 6.69E-09 | 2.11E-07 | 10.13902 |
| 5999 | -1.180135 | 5.4722098 | -6.94667 | 2.41E-09 | 9.44E-08 | 11.13161 |
| 6091 | 2.282202 | 8.447152 | 7.842695 | 6.49E-11 | 4.96E-09 | 14.65797 |
| 6241 | 2.2876938 | 7.0256514 | 8.447284 | 5.66E-12 | 7.29E-10 | 17.03877 |
| 6279 | -1.574572 | 6.1695409 | -5.31908 | 1.45E-06 | 1.70E-05 | 4.916955 |
| 6283 | -1.216494 | 5.1668013 | -5.81814 | 2.13E-07 | 3.51E-06 | 6.776228 |
| 6286 | 2.027613 | 6.3407998 | 3.583362 | 0.00065895 | 0.002945399 | -0.92621 |
| 6296 | -1.285581 | 7.1179395 | -5.37687 | 1.16E-06 | 1.43E-05 | 5.129348 |
| 6347 | -1.709688 | 8.377796 | -5.73979 | 2.88E-07 | 4.54E-06 | 6.480814 |
| 6358 | -1.443064 | 9.1246153 | -8.12995 | 2.04E-11 | 1.96E-09 | 15.79022 |
| 6363 | -2.167207 | 7.2417478 | -5.04423 | 4.08E-06 | 4.04E-05 | 3.919127 |
| 6366 | -1.060885 | 7.3508605 | -3.8248 | 0.00030159 | 0.001521954 | -0.191 |
| 6372 | -2.362526 | 5.5472214 | -6.19535 | 4.84E-08 | 1.06E-06 | 8.213646 |
| 6387 | -1.857241 | 8.8559349 | -7.37487 | 4.30E-10 | 2.31E-08 | 12.81392 |
| 6446 | -1.02341 | 10.345202 | -5.13012 | 2.96E-06 | 3.10E-05 | 4.228712 |
| 6462 | -1.984331 | 7.111606 | -10.2149 | 5.09E-15 | 3.58E-12 | 23.87953 |
| 6491 | 1.0220733 | 5.2311316 | 6.863932 | 3.37E-09 | 1.24E-07 | 10.8077 |
| 6505 | -1.494536 | 7.4217513 | -4.54102 | 2.57E-05 | 0.000189626 | 2.152766 |
| 6515 | -1.332228 | 6.9331761 | -5.94961 | 1.27E-07 | 2.31E-06 | 7.274501 |
| 6519 | -1.578315 | 6.2767942 | -5.1375 | 2.88E-06 | 3.03E-05 | 4.25541 |
| 6539 | -1.168642 | 7.6038782 | -5.82804 | 2.05E-07 | 3.40E-06 | 6.813661 |
| 6542 | -1.370171 | 6.3977404 | -8.33904 | 8.76E-12 | 1.02E-09 | 16.61327 |
| 6554 | -2.302533 | 10.261197 | -5.46388 | 8.35E-07 | 1.09E-05 | 5.450621 |
| 6580 | -3.028216 | 9.726483 | -6.17386 | 5.27E-08 | 1.14E-06 | 8.131147 |
| 6590 | -1.428409 | 9.1299835 | -3.94486 | 0.00020247 | 0.00108656 | 0.18565 |
| 6690 | 4.6227239 | 8.6579624 | 8.663824 | 2.37E-12 | 3.79E-10 | 17.88852 |
| 6694 | -1.920204 | 9.8478034 | -5.44985 | 8.81E-07 | 1.13E-05 | 5.398693 |
| 6696 | 1.0365574 | 6.6329733 | 3.162755 | 0.00239957 | 0.008609764 | -2.12926 |
| 6713 | 1.6473257 | 7.3477125 | 6.231943 | 4.19E-08 | 9.37E-07 | 8.35427 |
| 6715 | -1.060374 | 8.1615771 | -5.65739 | 3.97E-07 | 5.92E-06 | 6.17143 |
| 6716 | -1.14699 | 6.3764066 | -4.18995 | 8.81E-05 | 0.000537894 | 0.975803 |
| 6718 | -2.005411 | 8.7709802 | -4.12918 | 0.0001085 | 0.000644654 | 0.777329 |
| 6790 | 1.5925855 | 5.9152529 | 7.222609 | 7.95E-10 | 3.73E-08 | 12.21477 |
| 6817 | -1.121505 | 10.964434 | -5.17697 | 2.48E-06 | 2.66E-05 | 4.39845 |
| 6819 | 1.2959643 | 5.5516194 | 3.221551 | 0.00201424 | 0.00744937 | -1.96742 |
| 6894 | 1.1125173 | 8.099942 | 9.73777 | 3.30E-14 | 1.28E-11 | 22.0587 |
| 6898 | -1.763862 | 7.9681019 | -5.04695 | 4.04E-06 | 4.02E-05 | 3.928912 |
| 6905 | 1.1396458 | 7.6490704 | 9.041752 | 5.22E-13 | 1.21E-10 | 19.36554 |
| 6913 | -1.856725 | 6.1674468 | -7.77005 | 8.71E-11 | 6.23E-09 | 14.3715 |
| 6999 | -1.839891 | 11.011831 | -5.61542 | 4.66E-07 | 6.76E-06 | 6.014412 |
| 7013 | 1.0199778 | 7.6979376 | 7.087046 | 1.37E-09 | 5.89E-08 | 11.68212 |
| 7057 | -1.567193 | 7.627125 | -6.85422 | 3.50E-09 | 1.28E-07 | 10.76973 |
| 7069 | -3.153042 | 7.9906369 | -8.41984 | 6.33E-12 | 7.89E-10 | 16.93092 |
| 7076 | -1.228588 | 11.159613 | -4.33308 | 5.36E-05 | 0.000352059 | 1.449542 |
| 7086 | 1.091645 | 8.0045322 | 6.502824 | 1.43E-08 | 3.86E-07 | 9.40066 |
| 7098 | -1.280608 | 6.6906254 | -5.59294 | 5.09E-07 | 7.23E-06 | 5.930459 |
| 7099 | -1.049812 | 5.7319427 | -8.82305 | 1.25E-12 | 2.31E-10 | 18.51183 |
| 7113 | -1.012616 | 7.0929694 | -6.83212 | 3.82E-09 | 1.37E-07 | 10.68328 |
| 7153 | 2.5135333 | 5.1596763 | 7.847417 | 6.37E-11 | 4.90E-09 | 14.67659 |
| 7164 | -1.052383 | 8.2521421 | -6.63829 | 8.31E-09 | 2.50E-07 | 9.927076 |
| 7188 | 1.0531737 | 5.9754377 | 5.247163 | 1.90E-06 | 2.13E-05 | 4.653884 |
| 7203 | 1.2111715 | 10.284407 | 9.736782 | 3.31E-14 | 1.28E-11 | 22.05491 |
| 7272 | 1.7460372 | 4.3349816 | 6.237025 | 4.10E-08 | 9.21E-07 | 8.373814 |
| 7292 | 1.2320221 | 5.2300014 | 5.04946 | 4.00E-06 | 3.98E-05 | 3.937931 |
| 7296 | 1.3043529 | 9.7560878 | 6.375062 | 2.37E-08 | 5.82E-07 | 8.905998 |
| 7358 | 1.0692588 | 9.5355134 | 6.124373 | 6.40E-08 | 1.33E-06 | 7.941438 |
| 7360 | -1.394964 | 8.5083651 | -8.85065 | 1.12E-12 | 2.12E-10 | 18.61977 |
| 7429 | 1.0800898 | 5.1974172 | 3.178143 | 0.00229254 | 0.008298663 | -2.08711 |
| 7498 | -1.914147 | 8.2062402 | -6.79014 | 4.52E-09 | 1.54E-07 | 10.51925 |
| 7538 | -1.261724 | 9.3887952 | -8.86316 | 1.07E-12 | 2.04E-10 | 18.66864 |
| 7546 | 1.8231202 | 4.4203277 | 7.837964 | 6.62E-11 | 5.03E-09 | 14.63931 |
| 7802 | -1.122665 | 6.1377782 | -8.38016 | 7.42E-12 | 8.88E-10 | 16.77497 |
| 7980 | -1.625782 | 5.0662247 | -9.55209 | 6.87E-14 | 2.30E-11 | 21.34417 |
| 8379 | -1.008029 | 6.5560277 | -5.08515 | 3.50E-06 | 3.58E-05 | 4.066364 |
| 8404 | 1.074706 | 9.1197354 | 3.180102 | 0.00227924 | 0.008260733 | -2.08174 |
| 8406 | -2.459955 | 6.4678338 | -7.47746 | 2.84E-10 | 1.68E-08 | 13.21805 |
| 8424 | -1.673932 | 6.6375132 | -4.50366 | 2.93E-05 | 0.000211483 | 2.025151 |
| 8443 | 1.1793273 | 9.2063456 | 10.00145 | 1.17E-14 | 6.37E-12 | 23.06783 |
| 8490 | 1.0296503 | 8.3295039 | 4.813001 | 9.59E-06 | 8.20E-05 | 3.096931 |
| 8519 | -1.227228 | 10.229996 | -4.50455 | 2.92E-05 | 0.000210883 | 2.028209 |
| 8529 | -1.469647 | 9.4219406 | -5.83345 | 2.00E-07 | 3.36E-06 | 6.834117 |
| 8547 | -3.99867 | 8.2356829 | -12.6849 | 4.77E-19 | 1.22E-15 | 32.87965 |
| 8549 | 1.6370024 | 4.6138828 | 4.62381 | 1.91E-05 | 0.000147657 | 2.437355 |
| 8553 | -1.37921 | 8.3954795 | -7.03554 | 1.69E-09 | 6.98E-08 | 11.48001 |
| 8608 | -2.077708 | 9.80445 | -7.33936 | 4.96E-10 | 2.59E-08 | 12.67415 |
| 8613 | -1.194635 | 10.048215 | -10.2072 | 5.24E-15 | 3.58E-12 | 23.85024 |
| 8630 | -1.163892 | 11.584216 | -4.08206 | 0.00012743 | 0.000738257 | 0.624586 |
| 8638 | -1.058846 | 7.9859866 | -3.27944 | 0.00169225 | 0.006437709 | -1.80601 |
| 8644 | 1.249536 | 11.989462 | 8.683718 | 2.19E-12 | 3.56E-10 | 17.96648 |
| 8671 | -1.02289 | 6.2529857 | -5.05766 | 3.88E-06 | 3.88E-05 | 3.967397 |
| 8685 | -2.494682 | 7.4964759 | -11.7411 | 1.51E-17 | 2.07E-14 | 29.53224 |
| 8835 | -2.179541 | 7.9334402 | -6.66196 | 7.56E-09 | 2.35E-07 | 10.01926 |
| 8836 | 1.3724016 | 10.382634 | 7.338573 | 4.98E-10 | 2.59E-08 | 12.67103 |
| 8842 | -1.8191 | 4.623592 | -5.82473 | 2.07E-07 | 3.44E-06 | 6.801152 |
| 8853 | 1.1605603 | 6.2057567 | 6.236888 | 4.10E-08 | 9.21E-07 | 8.373291 |
| 8858 | -1.335633 | 7.1133699 | -6.84278 | 3.66E-09 | 1.32E-07 | 10.72499 |
| 8876 | -2.13161 | 8.8387145 | -5.42133 | 9.83E-07 | 1.24E-05 | 5.293275 |
| 9023 | -1.717482 | 5.4958548 | -6.94708 | 2.41E-09 | 9.44E-08 | 11.13319 |
| 9027 | -1.266997 | 9.3481334 | -5.37779 | 1.16E-06 | 1.42E-05 | 5.132704 |
| 9055 | 1.973807 | 5.2845919 | 8.769701 | 1.55E-12 | 2.74E-10 | 18.30316 |
| 9071 | -1.953082 | 5.7911107 | -8.28795 | 1.08E-11 | 1.20E-09 | 16.41227 |
| 9077 | -1.699193 | 5.1529377 | -11.1123 | 1.61E-16 | 1.65E-13 | 27.23735 |
| 9133 | 1.2709253 | 4.8949468 | 6.166651 | 5.42E-08 | 1.16E-06 | 8.103491 |
| 9134 | 1.174142 | 4.1094917 | 6.537826 | 1.24E-08 | 3.43E-07 | 9.536497 |
| 9154 | -1.039072 | 6.449655 | -6.18262 | 5.09E-08 | 1.10E-06 | 8.164769 |
| 9232 | 1.3305613 | 8.1321033 | 6.842187 | 3.67E-09 | 1.32E-07 | 10.72266 |
| 9319 | 1.151689 | 5.0236459 | 5.349423 | 1.29E-06 | 1.55E-05 | 5.028364 |
| 9332 | -1.491925 | 9.3142992 | -7.30593 | 5.68E-10 | 2.91E-08 | 12.54254 |
| 9388 | -1.146837 | 7.7081701 | -4.34815 | 5.08E-05 | 0.000336547 | 1.499922 |
| 9480 | 1.0446055 | 7.0905933 | 7.743458 | 9.70E-11 | 6.80E-09 | 14.26665 |
| 9506 | 1.3952179 | 5.6265991 | 3.911028 | 0.00022667 | 0.001198234 | 0.078806 |
| 9510 | -1.750527 | 6.9680793 | -7.02484 | 1.76E-09 | 7.17E-08 | 11.43802 |
| 9547 | -2.633957 | 6.0105539 | -11.069 | 1.90E-16 | 1.85E-13 | 27.07774 |
| 9615 | -1.57995 | 9.236812 | -4.68051 | 1.55E-05 | 0.000123876 | 2.633725 |
| 9630 | -1.136906 | 5.99089 | -9.15657 | 3.30E-13 | 8.98E-11 | 19.81244 |
| 9768 | 1.2765727 | 6.8970592 | 7.076714 | 1.43E-09 | 6.10E-08 | 11.64157 |
| 9787 | 1.8208523 | 4.5741697 | 8.189437 | 1.60E-11 | 1.65E-09 | 16.02449 |
| 9833 | 1.5057106 | 5.9269654 | 6.797651 | 4.39E-09 | 1.51E-07 | 10.54859 |
| 9837 | 1.8464967 | 5.0931803 | 7.073431 | 1.45E-09 | 6.17E-08 | 11.62868 |
| 9886 | 1.3767428 | 6.1937322 | 4.724561 | 1.32E-05 | 0.000108239 | 2.787052 |
| 9891 | 1.0719898 | 7.1594242 | 8.070557 | 2.59E-11 | 2.35E-09 | 15.55621 |
| 9918 | 1.1133705 | 5.2139733 | 7.109488 | 1.25E-09 | 5.43E-08 | 11.77024 |
| 9920 | -1.046797 | 4.9615384 | -7.90906 | 4.97E-11 | 4.03E-09 | 14.91963 |
| 9928 | 1.1518338 | 4.3598301 | 6.625457 | 8.75E-09 | 2.61E-07 | 9.877139 |
| 10112 | 1.7951575 | 4.6283707 | 7.309604 | 5.59E-10 | 2.88E-08 | 12.55701 |
| 10164 | -1.807203 | 5.8108091 | -7.61871 | 1.61E-10 | 1.03E-08 | 13.77476 |
| 10202 | 1.2191565 | 6.0000007 | 3.403048 | 0.00115971 | 0.004731097 | -1.45462 |
| 10216 | -1.570949 | 6.3747629 | -5.04144 | 4.12E-06 | 4.07E-05 | 3.90911 |
| 10249 | -1.659557 | 7.7919178 | -5.25667 | 1.84E-06 | 2.07E-05 | 4.688593 |
| 10267 | 1.314324 | 7.3900799 | 5.975306 | 1.15E-07 | 2.14E-06 | 7.372263 |
| 10332 | -2.354233 | 6.0992262 | -13.1731 | 8.33E-20 | 5.09E-16 | 34.56361 |
| 10346 | -1.425237 | 9.4730926 | -5.49735 | 7.35E-07 | 9.74E-06 | 5.574691 |
| 10351 | -1.891558 | 8.8409051 | -5.60389 | 4.88E-07 | 7.00E-06 | 5.971339 |
| 10400 | -1.208033 | 9.0111503 | -7.8091 | 7.44E-11 | 5.51E-09 | 14.52551 |
| 10403 | 1.7698197 | 5.0339408 | 8.041987 | 2.90E-11 | 2.54E-09 | 15.44362 |
| 10486 | 1.8340837 | 6.7810182 | 12.24193 | 2.38E-18 | 3.75E-15 | 31.32337 |
| 10516 | -1.058178 | 7.6449071 | -3.83907 | 0.00028774 | 0.001462129 | -0.14661 |
| 10535 | 1.0834997 | 6.7491426 | 7.772172 | 8.64E-11 | 6.20E-09 | 14.37987 |
| 10551 | 1.0659588 | 4.2526936 | 2.541285 | 0.01350424 | 0.035564646 | -3.70038 |
| 10584 | -1.504592 | 5.5250743 | -12.801 | 3.14E-19 | 1.07E-15 | 33.28284 |
| 10635 | 1.3239831 | 4.7012036 | 6.13136 | 6.23E-08 | 1.30E-06 | 7.968199 |
| 10643 | 1.9870663 | 4.5112071 | 5.27662 | 1.70E-06 | 1.95E-05 | 4.761476 |
| 10653 | -1.276648 | 6.6096952 | -7.21116 | 8.32E-10 | 3.86E-08 | 12.16975 |
| 10732 | 1.3555961 | 6.1135962 | 11.2743 | 8.72E-17 | 9.67E-14 | 27.83347 |
| 10786 | -1.749841 | 8.2731455 | -6.77021 | 4.90E-09 | 1.64E-07 | 10.44142 |
| 10864 | -1.61321 | 8.909715 | -5.51161 | 6.96E-07 | 9.32E-06 | 5.627635 |
| 10891 | -1.257576 | 7.632719 | -3.86907 | 0.00026057 | 0.001348193 | -0.05295 |
| 10894 | -2.626065 | 6.53689 | -12.9128 | 2.10E-19 | 8.61E-16 | 33.6699 |
| 10974 | -1.26439 | 7.5237798 | -3.38817 | 0.00121419 | 0.004915843 | -1.49739 |
| 10990 | -1.090024 | 5.8286741 | -10.2955 | 3.72E-15 | 2.82E-12 | 24.18477 |
| 10993 | -2.486802 | 10.036979 | -5.3368 | 1.36E-06 | 1.62E-05 | 4.981985 |
| 11065 | 1.4968072 | 6.8271588 | 6.118398 | 6.55E-08 | 1.35E-06 | 7.918556 |
| 11082 | 2.1351663 | 4.6469657 | 8.011842 | 3.28E-11 | 2.82E-09 | 15.32482 |
| 11130 | 1.7605602 | 7.0788618 | 7.431967 | 3.41E-10 | 1.93E-08 | 13.03881 |
| 11185 | -1.711244 | 6.8497482 | -7.39536 | 3.96E-10 | 2.15E-08 | 12.89461 |
| 11199 | -1.585268 | 8.50515 | -4.43381 | 3.76E-05 | 0.000260884 | 1.788049 |
| 11234 | -1.014139 | 8.2662102 | -5.68894 | 3.51E-07 | 5.33E-06 | 6.289752 |
| 11326 | -1.483939 | 7.8122276 | -8.40867 | 6.62E-12 | 8.11E-10 | 16.88702 |
| 11343 | -1.26603 | 8.9139444 | -6.55466 | 1.16E-08 | 3.25E-07 | 9.601866 |
| 22809 | -1.054989 | 7.2069178 | -4.68005 | 1.56E-05 | 0.000124036 | 2.632114 |
| 22822 | -1.096048 | 7.2829513 | -5.496 | 7.39E-07 | 9.79E-06 | 5.569691 |
| 22824 | -1.034535 | 6.7910293 | -4.51416 | 2.82E-05 | 0.000205161 | 2.060959 |
| 22849 | -1.379015 | 6.4928077 | -6.69505 | 6.62E-09 | 2.10E-07 | 10.14821 |
| 22915 | -1.092737 | 4.3300242 | -5.5786 | 5.38E-07 | 7.53E-06 | 5.876951 |
| 22943 | 1.7369324 | 4.9124803 | 3.554394 | 0.00072238 | 0.003174157 | -1.01233 |
| 22974 | 1.142879 | 6.4434502 | 4.526598 | 2.70E-05 | 0.000198082 | 2.103435 |
| 22977 | -1.359493 | 9.338033 | -5.33765 | 1.35E-06 | 1.61E-05 | 4.985123 |
| 23089 | 2.2578916 | 5.9161181 | 4.232231 | 7.61E-05 | 0.00047356 | 1.114833 |
| 23151 | -1.291949 | 7.6336031 | -4.46083 | 3.41E-05 | 0.000240664 | 1.879537 |
| 23676 | 1.3733013 | 4.7936237 | 5.097411 | 3.35E-06 | 3.44E-05 | 4.110559 |
| 23708 | -1.51348 | 6.0157459 | -6.69276 | 6.68E-09 | 2.11E-07 | 10.13927 |
| 23710 | -1.042453 | 8.3072437 | -7.30107 | 5.79E-10 | 2.95E-08 | 12.52342 |
| 23764 | -1.0948 | 7.2223718 | -4.49389 | 3.04E-05 | 0.000217892 | 1.991884 |
| 23767 | -1.183133 | 7.1376752 | -5.40734 | 1.04E-06 | 1.30E-05 | 5.241618 |
| 24137 | 1.7802091 | 4.7650002 | 6.603231 | 9.56E-09 | 2.78E-07 | 9.790667 |
| 25854 | -1.185115 | 6.5313039 | -6.21953 | 4.40E-08 | 9.81E-07 | 8.30653 |
| 25879 | 1.3033638 | 7.5376078 | 7.546069 | 2.15E-10 | 1.34E-08 | 13.48842 |
| 25909 | 1.1023667 | 7.0408859 | 9.786127 | 2.73E-14 | 1.14E-11 | 22.24427 |
| 25984 | -1.50126 | 8.1136742 | -4.09194 | 0.00012321 | 0.000718519 | 0.65652 |
| 26227 | -1.097545 | 6.9587488 | -4.48254 | 3.16E-05 | 0.000225601 | 1.953276 |
| 26291 | 1.0964647 | 6.1270879 | 4.122866 | 0.00011087 | 0.000657691 | 0.756805 |
| 26499 | -1.004353 | 7.4243904 | -3.85683 | 0.00027135 | 0.00139302 | -0.09122 |
| 26577 | 1.6498381 | 5.8471971 | 6.266741 | 3.65E-08 | 8.38E-07 | 8.488168 |
| 26751 | -1.86093 | 7.7894956 | -8.12744 | 2.06E-11 | 1.96E-09 | 15.78032 |
| 27111 | -1.229476 | 7.0114609 | -9.6082 | 5.50E-14 | 2.01E-11 | 21.56042 |
| 27165 | -3.24081 | 7.5128582 | -9.13955 | 3.53E-13 | 9.04E-11 | 19.74624 |
| 27232 | -1.119947 | 8.095743 | -2.82907 | 0.00624448 | 0.018903163 | -3.00568 |
| 27289 | -1.42329 | 8.0467744 | -4.83132 | 8.97E-06 | 7.75E-05 | 3.161456 |
| 27347 | 1.1199205 | 5.3087692 | 4.73016 | 1.30E-05 | 0.000106526 | 2.806592 |
| 28234 | -2.82493 | 8.0850127 | -5.44755 | 8.89E-07 | 1.14E-05 | 5.390171 |
| 28982 | 1.5992676 | 6.20584 | 8.935459 | 7.98E-13 | 1.68E-10 | 18.95101 |
| 29028 | 1.7633315 | 6.6450645 | 8.453203 | 5.53E-12 | 7.26E-10 | 17.06203 |
| 29089 | 1.9688484 | 5.7237343 | 8.178695 | 1.67E-11 | 1.69E-09 | 15.98219 |
| 29097 | 1.0451879 | 7.2542385 | 8.059739 | 2.70E-11 | 2.41E-09 | 15.51358 |
| 29127 | 2.1813843 | 7.1079243 | 10.0478 | 9.77E-15 | 5.71E-12 | 23.24448 |
| 29901 | 1.1895054 | 6.1423772 | 8.760068 | 1.61E-12 | 2.80E-10 | 18.26546 |
| 29940 | -1.262646 | 7.3477759 | -6.03745 | 9.01E-08 | 1.75E-06 | 7.609115 |
| 50486 | -1.539288 | 9.418124 | -5.31734 | 1.46E-06 | 1.71E-05 | 4.910569 |
| 51166 | -1.784783 | 6.417074 | -9.55189 | 6.87E-14 | 2.30E-11 | 21.34341 |
| 51175 | -1.567789 | 7.5331307 | -10.7029 | 7.70E-16 | 6.85E-13 | 25.71718 |
| 51179 | -1.856646 | 8.5090585 | -5.31027 | 1.50E-06 | 1.75E-05 | 4.884671 |
| 51203 | 1.6219226 | 6.2197515 | 7.524546 | 2.35E-10 | 1.43E-08 | 13.40359 |
| 51266 | -2.73686 | 7.2213845 | -12.7043 | 4.45E-19 | 1.22E-15 | 32.94699 |
| 51302 | -2.762427 | 7.3137939 | -9.99899 | 1.18E-14 | 6.37E-12 | 23.05844 |
| 51316 | -2.712625 | 7.2798127 | -8.76416 | 1.59E-12 | 2.77E-10 | 18.28149 |
| 51514 | 1.5750557 | 5.3702095 | 7.056474 | 1.55E-09 | 6.52E-08 | 11.56213 |
| 51633 | 1.1333899 | 6.0680541 | 7.227095 | 7.80E-10 | 3.68E-08 | 12.23241 |
| 51655 | -1.568828 | 8.3157683 | -6.28261 | 3.42E-08 | 7.94E-07 | 8.549298 |
| 53345 | -1.003635 | 6.5622755 | -8.97795 | 6.74E-13 | 1.47E-10 | 19.11682 |
| 53829 | -1.352759 | 5.6891523 | -6.53222 | 1.27E-08 | 3.50E-07 | 9.514715 |
| 54112 | 1.4826012 | 5.8646167 | 3.47905 | 0.00091567 | 0.003882566 | -1.23413 |
| 54363 | -1.021806 | 10.557493 | -4.19897 | 8.54E-05 | 0.00052408 | 1.005399 |
| 54443 | 1.8108524 | 4.2303102 | 6.709236 | 6.26E-09 | 2.01E-07 | 10.2035 |
| 54762 | -1.963883 | 7.2289643 | -7.13988 | 1.11E-09 | 4.93E-08 | 11.8896 |
| 54810 | -1.233807 | 5.5357089 | -5.60732 | 4.81E-07 | 6.92E-06 | 5.984154 |
| 54825 | -1.2698 | 7.3634102 | -7.87194 | 5.77E-11 | 4.54E-09 | 14.77327 |
| 54829 | -1.368958 | 8.4051057 | -5.02917 | 4.32E-06 | 4.23E-05 | 3.865077 |
| 54886 | 2.0741728 | 7.9194343 | 8.301986 | 1.02E-11 | 1.16E-09 | 16.46749 |
| 54959 | 1.5388503 | 4.5136614 | 3.897949 | 0.00023676 | 0.001244474 | 0.037642 |
| 54988 | -1.05143 | 9.6684163 | -3.3832 | 0.00123291 | 0.004979099 | -1.51164 |
| 55076 | -3.343623 | 9.1795716 | -9.50496 | 8.27E-14 | 2.69E-11 | 21.16232 |
| 55089 | -1.253331 | 9.8066405 | -5.40971 | 1.03E-06 | 1.29E-05 | 5.250391 |
| 55093 | 1.2287417 | 6.5572888 | 7.206905 | 8.46E-10 | 3.92E-08 | 12.15303 |
| 55156 | 1.066396 | 8.7330831 | 8.380253 | 7.42E-12 | 8.88E-10 | 16.77532 |
| 55165 | 1.061139 | 3.7824409 | 4.987729 | 5.03E-06 | 4.80E-05 | 3.716697 |
| 55240 | -1.741619 | 7.5510891 | -9.11452 | 3.90E-13 | 9.86E-11 | 19.64886 |
| 55329 | 1.1981544 | 4.6879387 | 3.69051 | 0.00046739 | 0.002207815 | -0.60372 |
| 55355 | 1.0070147 | 5.1105315 | 5.604459 | 4.87E-07 | 6.99E-06 | 5.973458 |
| 55356 | -1.434342 | 5.5585073 | -6.6571 | 7.71E-09 | 2.38E-07 | 10.00034 |
| 55532 | 1.0837894 | 8.5512428 | 4.914089 | 6.61E-06 | 6.03E-05 | 3.454308 |
| 55576 | -1.294148 | 6.2201647 | -12.2453 | 2.36E-18 | 3.75E-15 | 31.3352 |
| 55656 | 1.409785 | 7.7727423 | 9.490001 | 8.78E-14 | 2.81E-11 | 21.10458 |
| 55664 | -1.283186 | 8.4624044 | -6.97059 | 2.19E-09 | 8.66E-08 | 11.22533 |
| 55732 | 1.4063477 | 4.7467784 | 7.46486 | 2.99E-10 | 1.75E-08 | 13.16839 |
| 55753 | -1.208356 | 8.0849969 | -3.8081 | 0.00031862 | 0.001597247 | -0.24282 |
| 55796 | 1.1279274 | 7.4583382 | 6.161763 | 5.52E-08 | 1.18E-06 | 8.08474 |
| 55872 | 2.0294812 | 4.6906835 | 6.849098 | 3.57E-09 | 1.29E-07 | 10.74968 |
| 55959 | -1.288439 | 6.7271761 | -6.17921 | 5.15E-08 | 1.12E-06 | 8.1517 |
| 56606 | -1.239197 | 7.1948206 | -6.16452 | 5.46E-08 | 1.17E-06 | 8.095328 |
| 56892 | -1.567953 | 8.199349 | -7.56644 | 1.98E-10 | 1.24E-08 | 13.5687 |
| 56944 | -1.546063 | 6.6142456 | -6.87856 | 3.17E-09 | 1.18E-07 | 10.86493 |
| 57007 | -1.666664 | 7.5140512 | -5.09923 | 3.32E-06 | 3.42E-05 | 4.117128 |
| 57016 | 1.4822906 | 11.545176 | 3.129079 | 0.00265036 | 0.009350757 | -2.22098 |
| 57088 | -1.263411 | 8.4984336 | -8.41529 | 6.44E-12 | 7.94E-10 | 16.91305 |
| 57111 | -1.075845 | 5.06722 | -7.205 | 8.53E-10 | 3.94E-08 | 12.14554 |
| 57127 | 1.2987759 | 7.0843317 | 4.152402 | 0.0001002 | 0.000601274 | 0.852975 |
| 57134 | -1.393183 | 6.3744149 | -8.90535 | 9.01E-13 | 1.82E-10 | 18.83344 |
| 57181 | 1.0022508 | 6.4884266 | 5.986874 | 1.10E-07 | 2.06E-06 | 7.416306 |
| 57393 | -3.414561 | 6.6887259 | -10.0337 | 1.03E-14 | 5.87E-12 | 23.19091 |
| 57447 | -1.308096 | 8.3580926 | -10.0711 | 8.92E-15 | 5.53E-12 | 23.33326 |
| 57512 | 1.4845565 | 4.4443316 | 6.985212 | 2.07E-09 | 8.29E-08 | 11.28263 |
| 57733 | -2.368247 | 8.1893692 | -6.69602 | 6.60E-09 | 2.10E-07 | 10.15197 |
| 57817 | -1.911404 | 9.1346916 | -3.43932 | 0.00103644 | 0.004302824 | -1.34982 |
| 63979 | 1.015333 | 5.7019721 | 7.391452 | 4.02E-10 | 2.18E-08 | 12.87923 |
| 64081 | -1.197114 | 9.1593807 | -5.7997 | 2.28E-07 | 3.72E-06 | 6.706607 |
| 64092 | -1.100676 | 6.6021958 | -5.01853 | 4.49E-06 | 4.38E-05 | 3.826927 |
| 64105 | 1.2476227 | 4.0942643 | 5.753114 | 2.74E-07 | 4.36E-06 | 6.530975 |
| 64151 | 1.8008031 | 4.5134579 | 7.41689 | 3.63E-10 | 2.02E-08 | 12.97942 |
| 64231 | -1.246691 | 9.1162084 | -6.91354 | 2.76E-09 | 1.05E-07 | 11.00186 |
| 64388 | -1.469739 | 5.6116495 | -6.19952 | 4.76E-08 | 1.05E-06 | 8.229672 |
| 64577 | -1.797148 | 10.637267 | -5.89642 | 1.57E-07 | 2.75E-06 | 7.072568 |
| 64651 | -1.31158 | 6.7720824 | -7.75054 | 9.42E-11 | 6.65E-09 | 14.29457 |
| 64754 | 1.3672336 | 7.264655 | 8.739968 | 1.75E-12 | 2.98E-10 | 18.18678 |
| 64838 | -1.044821 | 7.7122532 | -7.69747 | 1.17E-10 | 7.89E-09 | 14.08532 |
| 64850 | -1.584121 | 9.7865019 | -5.00896 | 4.65E-06 | 4.49E-05 | 3.792642 |
| 64902 | -1.1905 | 8.4196549 | -5.39022 | 1.11E-06 | 1.37E-05 | 5.178489 |
| 65265 | 1.0656512 | 6.9684117 | 5.812225 | 2.18E-07 | 3.58E-06 | 6.753896 |
| 78989 | -1.765319 | 8.8606882 | -6.63947 | 8.27E-09 | 2.50E-07 | 9.931696 |
| 79022 | 1.1496749 | 8.773354 | 8.63859 | 2.62E-12 | 4.07E-10 | 17.78962 |
| 79054 | -1.18463 | 6.2876339 | -4.07802 | 0.00012919 | 0.000746361 | 0.611537 |
| 79191 | 1.189441 | 4.379522 | 5.071482 | 3.69E-06 | 3.71E-05 | 4.01713 |
| 79573 | 1.0009042 | 7.4110261 | 8.984717 | 6.56E-13 | 1.44E-10 | 19.1432 |
| 79660 | -1.386001 | 7.9657969 | -4.87631 | 7.60E-06 | 6.75E-05 | 3.320353 |
| 79689 | -1.534329 | 6.2499884 | -7.44423 | 3.25E-10 | 1.87E-08 | 13.08711 |
| 79733 | 1.1323838 | 4.1018387 | 5.495517 | 7.40E-07 | 9.80E-06 | 5.567894 |
| 79804 | -1.08293 | 3.9423046 | -5.71614 | 3.16E-07 | 4.89E-06 | 6.391898 |
| 79974 | -1.042343 | 7.2659651 | -6.4382 | 1.85E-08 | 4.75E-07 | 9.150215 |
| 80168 | -1.215313 | 5.9138079 | -8.63594 | 2.65E-12 | 4.08E-10 | 17.77923 |
| 80704 | -1.416486 | 6.1679212 | -9.01856 | 5.73E-13 | 1.31E-10 | 19.27514 |
| 81578 | 1.0975919 | 5.7565548 | 3.283488 | 0.00167165 | 0.006373563 | -1.79464 |
| 81610 | 2.2110894 | 6.1008197 | 8.455281 | 5.49E-12 | 7.24E-10 | 17.07019 |
| 83483 | 1.1550433 | 7.4415467 | 8.066392 | 2.63E-11 | 2.38E-09 | 15.5398 |
| 83539 | -1.541842 | 5.4697071 | -6.05739 | 8.33E-08 | 1.64E-06 | 7.685237 |
| 83540 | 1.5871628 | 4.0059287 | 5.642817 | 4.20E-07 | 6.19E-06 | 6.116877 |
| 83597 | -1.025333 | 8.0150804 | -3.03548 | 0.00348215 | 0.011692018 | -2.47214 |
| 83716 | -1.415605 | 6.6955725 | -4.724 | 1.33E-05 | 0.000108417 | 2.785089 |
| 83729 | -1.957499 | 8.1186451 | -6.03882 | 8.96E-08 | 1.75E-06 | 7.614344 |
| 83875 | -2.432972 | 7.0492983 | -9.10474 | 4.06E-13 | 1.01E-10 | 19.61083 |
| 83953 | -1.294469 | 6.0114599 | -4.46272 | 3.39E-05 | 0.000239303 | 1.88595 |
| 84079 | 1.3587632 | 6.8204771 | 9.438131 | 1.08E-13 | 3.39E-11 | 20.90416 |
| 84102 | -1.106103 | 7.2390601 | -5.58175 | 5.31E-07 | 7.46E-06 | 5.888718 |
| 84134 | 1.1386118 | 6.5202552 | 8.955208 | 7.38E-13 | 1.57E-10 | 19.02809 |
| 84171 | -1.546898 | 8.2523251 | -5.13285 | 2.93E-06 | 3.08E-05 | 4.238575 |
| 84320 | 1.010506 | 6.7606462 | 11.97413 | 6.38E-18 | 9.33E-15 | 30.36976 |
| 84457 | 1.1418987 | 5.8402825 | 5.323643 | 1.43E-06 | 1.68E-05 | 4.933702 |
| 84619 | -1.466593 | 8.447853 | -6.62752 | 8.68E-09 | 2.60E-07 | 9.885172 |
| 84675 | 1.1705304 | 6.6004013 | 3.395175 | 0.00118825 | 0.00482729 | -1.47727 |
| 84735 | -1.947055 | 6.4817319 | -5.08108 | 3.56E-06 | 3.62E-05 | 4.051673 |
| 84870 | -1.501891 | 5.174934 | -11.5344 | 3.28E-17 | 4.19E-14 | 28.78351 |
| 84873 | -2.222899 | 5.5823108 | -9.18339 | 2.97E-13 | 8.32E-11 | 19.9167 |
| 84879 | -2.883578 | 7.8114483 | -10.426 | 2.24E-15 | 1.83E-12 | 24.67779 |
| 84918 | 1.0836196 | 6.9709776 | 5.551531 | 5.97E-07 | 8.20E-06 | 5.776088 |
| 84955 | 1.0372732 | 6.3646998 | 6.764918 | 5.01E-09 | 1.67E-07 | 10.42075 |
| 84988 | 1.3036695 | 8.4774731 | 7.803198 | 7.62E-11 | 5.59E-09 | 14.50222 |
| 85480 | -1.453334 | 5.2137242 | -6.43426 | 1.88E-08 | 4.81E-07 | 9.134945 |
| 90865 | -1.795958 | 6.8211454 | -9.25313 | 2.25E-13 | 6.48E-11 | 20.18752 |
| 91316 | -1.478652 | 9.8852632 | -3.21507 | 0.00205369 | 0.007563842 | -1.98538 |
| 91464 | 1.0315345 | 4.3856517 | 4.193636 | 8.69E-05 | 0.000532377 | 0.987887 |
| 91614 | -1.133931 | 8.7030483 | -5.27809 | 1.69E-06 | 1.95E-05 | 4.766837 |
| 91937 | -2.07549 | 5.6352728 | -9.88907 | 1.82E-14 | 8.46E-12 | 22.63857 |
| 91947 | -1.226058 | 7.2160998 | -3.87102 | 0.0002589 | 0.001342237 | -0.04685 |
| 92292 | -2.032247 | 8.6993511 | -6.10131 | 7.01E-08 | 1.43E-06 | 7.853149 |
| 92840 | -1.021183 | 9.7659004 | -4.60876 | 2.01E-05 | 0.00015413 | 2.385422 |
| 93594 | 1.1706535 | 5.1184719 | 8.154593 | 1.84E-11 | 1.84E-09 | 15.88727 |
| 114770 | -2.498735 | 9.3109456 | -7.90728 | 5.00E-11 | 4.05E-09 | 14.91262 |
| 114899 | 1.1504707 | 6.6735694 | 4.386438 | 4.44E-05 | 0.0003013 | 1.628354 |
| 115908 | 1.6263617 | 5.5814995 | 4.092619 | 0.00012293 | 0.000717263 | 0.658722 |
| 116519 | -1.306258 | 9.1547567 | -6.00093 | 1.04E-07 | 1.98E-06 | 7.469835 |
| 116844 | -1.160349 | 10.256607 | -5.27307 | 1.73E-06 | 1.98E-05 | 4.748492 |
| 119467 | -3.227656 | 7.6382659 | -8.43163 | 6.03E-12 | 7.57E-10 | 16.97725 |
| 120224 | -1.056521 | 8.6369238 | -3.21965 | 0.00202575 | 0.007477066 | -1.9727 |
| 122622 | -1.707825 | 6.8465361 | -7.43448 | 3.38E-10 | 1.93E-08 | 13.04872 |
| 122786 | -1.428094 | 7.5135758 | -7.22971 | 7.72E-10 | 3.66E-08 | 12.24271 |
| 128346 | -1.056708 | 7.5430384 | -6.3998 | 2.15E-08 | 5.38E-07 | 9.001617 |
| 131669 | -1.128826 | 5.7397211 | -7.52476 | 2.35E-10 | 1.43E-08 | 13.40444 |
| 134548 | 1.1645701 | 5.859656 | 5.441745 | 9.09E-07 | 1.16E-05 | 5.368717 |
| 135112 | -1.106905 | 10.044966 | -5.30103 | 1.55E-06 | 1.81E-05 | 4.850796 |
| 137695 | 1.2657994 | 5.9104966 | 8.627817 | 2.74E-12 | 4.19E-10 | 17.74738 |
| 139221 | -1.312386 | 4.0336146 | -9.0174 | 5.75E-13 | 1.31E-10 | 19.27064 |
| 140809 | 1.5168707 | 8.5524224 | 7.234694 | 7.57E-10 | 3.64E-08 | 12.2623 |
| 143941 | -2.298136 | 7.8672269 | -8.70195 | 2.04E-12 | 3.41E-10 | 18.03792 |
| 144193 | -1.152159 | 9.8235649 | -5.25485 | 1.85E-06 | 2.08E-05 | 4.681922 |
| 145389 | 1.2862669 | 6.6817835 | 8.648551 | 2.52E-12 | 3.94E-10 | 17.82866 |
| 147372 | -1.22075 | 5.0422924 | -8.99237 | 6.36E-13 | 1.41E-10 | 19.17304 |
| 150094 | -1.017324 | 8.0203659 | -5.33036 | 1.39E-06 | 1.65E-05 | 4.958344 |
| 150381 | 1.1374784 | 6.3224776 | 5.033102 | 4.25E-06 | 4.18E-05 | 3.879193 |
| 151246 | 1.3334376 | 4.350957 | 6.89113 | 3.02E-09 | 1.13E-07 | 10.91412 |
| 163786 | 1.0727413 | 5.1344865 | 6.601537 | 9.63E-09 | 2.79E-07 | 9.784081 |
| 169355 | -1.765129 | 5.1648477 | -5.22267 | 2.09E-06 | 2.29E-05 | 4.564587 |
| 170392 | -3.740668 | 6.8787581 | -13.1228 | 9.96E-20 | 5.09E-16 | 34.39156 |
| 170712 | 1.6362701 | 4.9886065 | 4.560168 | 2.40E-05 | 0.000178555 | 2.218349 |
| 196410 | -1.147652 | 10.208603 | -5.68664 | 3.54E-07 | 5.36E-06 | 6.281113 |
| 200931 | -2.218274 | 9.8496705 | -5.56333 | 5.70E-07 | 7.92E-06 | 5.82002 |
| 201799 | -1.060227 | 5.2854613 | -6.44355 | 1.81E-08 | 4.69E-07 | 9.17092 |
| 221662 | 1.9102416 | 4.7653753 | 7.059492 | 1.53E-09 | 6.45E-08 | 11.57397 |
| 259266 | 1.8044984 | 4.4126522 | 8.669838 | 2.32E-12 | 3.73E-10 | 17.91209 |
| 283377 | -1.09014 | 8.1828331 | -8.25099 | 1.25E-11 | 1.35E-09 | 16.2668 |
| 283537 | -1.518309 | 8.939359 | -4.87953 | 7.51E-06 | 6.69E-05 | 3.331759 |
| 283600 | -2.700046 | 7.832878 | -9.72779 | 3.43E-14 | 1.30E-11 | 22.02038 |
| 284013 | -1.207032 | 5.6082855 | -8.13622 | 1.98E-11 | 1.94E-09 | 15.81489 |
| 284422 | -1.34234 | 6.1043312 | -5.26046 | 1.81E-06 | 2.05E-05 | 4.702411 |
| 284618 | 1.0147548 | 8.7742729 | 5.867646 | 1.75E-07 | 3.01E-06 | 6.963503 |
| 285016 | -1.451207 | 4.5744833 | -7.7416 | 9.77E-11 | 6.82E-09 | 14.25934 |
| 285440 | -1.457254 | 9.0149179 | -6.77437 | 4.82E-09 | 1.62E-07 | 10.45764 |
| 286101 | 1.0394981 | 7.1340047 | 8.464848 | 5.28E-12 | 7.01E-10 | 17.10777 |
| 339390 | -3.286159 | 6.4137649 | -15.7438 | 1.41E-23 | 2.88E-19 | 42.88992 |
| 341640 | -1.333099 | 3.630572 | -7.10049 | 1.30E-09 | 5.60E-08 | 11.73491 |
| 344887 | 2.024 | 5.1784925 | 7.232857 | 7.62E-10 | 3.65E-08 | 12.25507 |
| 360023 | 1.1615202 | 7.274053 | 7.783472 | 8.25E-11 | 5.99E-09 | 14.42443 |
| 387103 | 1.4026718 | 6.0720679 | 6.590003 | 1.01E-08 | 2.91E-07 | 9.739228 |
| 387763 | -1.266391 | 7.5019585 | -7.8518 | 6.26E-11 | 4.83E-09 | 14.69386 |
| 388503 | -1.483582 | 9.5706252 | -4.64673 | 1.76E-05 | 0.000137555 | 2.516605 |
| 388753 | 1.0133285 | 8.1752254 | 8.316829 | 9.58E-12 | 1.10E-09 | 16.52589 |
| 401466 | 1.2205125 | 8.6970375 | 8.187371 | 1.61E-11 | 1.65E-09 | 16.01635 |
| 619208 | -1.161824 | 6.7054388 | -7.53544 | 2.25E-10 | 1.39E-08 | 13.44652 |
| 642273 | -2.195781 | 7.9365807 | -6.32227 | 2.93E-08 | 6.94E-07 | 8.702168 |
| 646962 | 1.0110089 | 6.2551283 | 4.819422 | 9.37E-06 | 8.03E-05 | 3.119531 |
| 653808 | -1.349453 | 7.1833208 | -5.62478 | 4.50E-07 | 6.56E-06 | 6.049385 |
| 1E+08 | 1.5623076 | 5.4412217 | 7.540538 | 2.20E-10 | 1.37E-08 | 13.46662 |
| 1.01E+08 | 1.0255917 | 6.8078567 | 7.278173 | 6.35E-10 | 3.17E-08 | 12.43333 |
| 1.01E+08 | -1.516582 | 11.423715 | -5.39626 | 1.08E-06 | 1.34E-05 | 5.200787 |
| 1.01E+08 | 1.4497979 | 4.8612226 | 5.807951 | 2.21E-07 | 3.62E-06 | 6.737756 |
| 1.02E+08 | -1.086426 | 5.514182 | -5.49257 | 7.48E-07 | 9.89E-06 | 5.55696 |
