# Supplementary material for: Long non-coding RNA expression profiles of hepatitis C virus-related dysplasia and hepatocellular carcinoma
Source: Oncotarget. 2015 Oct 26;6(41):43770–8. doi: 10.18632/oncotarget.6087 (PMC4791265; doi:10.18632/oncotarget.6087)
Supplement: Supplementary file 3 [file oncotarget-06-43770-s003.docx]

| Annotation Cluster 1 | Enrichment Score: 17.114396329538 | | | |  |  |  |  |
| --- | --- | --- | --- | --- | --- | --- | --- | --- |
| Category | Term | Count | % | PValue | Fold Enrichment | Bonferroni | Benjamini | FDR |
| GOTERM_BP_FAT | GO:0000279~M phase | 33 | 17.74194 | 1.62E-21 | 8.986171 | 1.86E-18 | 1.86E-18 | 2.61E-18 |
| GOTERM_BP_FAT | GO:0000280~nuclear division | 28 | 15.05376 | 7.14E-21 | 11.40229 | 8.20E-18 | 4.10E-18 | 1.15E-17 |
| GOTERM_BP_FAT | GO:0007067~mitosis | 28 | 15.05376 | 7.14E-21 | 11.40229 | 8.20E-18 | 4.10E-18 | 1.15E-17 |
| GOTERM_BP_FAT | GO:0000087~M phase of mitotic cell cycle | 28 | 15.05376 | 1.15E-20 | 11.19868 | 1.32E-17 | 4.42E-18 | 1.86E-17 |
| GOTERM_BP_FAT | GO:0048285~organelle fission | 28 | 15.05376 | 2.08E-20 | 10.95416 | 2.38E-17 | 5.96E-18 | 3.34E-17 |
| GOTERM_BP_FAT | GO:0022402~cell cycle process | 39 | 20.96774 | 7.20E-20 | 6.184047 | 8.26E-17 | 1.65E-17 | 1.16E-16 |
| GOTERM_BP_FAT | GO:0022403~cell cycle phase | 34 | 18.27957 | 1.72E-19 | 7.357584 | 1.98E-16 | 3.30E-17 | 2.77E-16 |
| GOTERM_BP_FAT | GO:0000278~mitotic cell cycle | 32 | 17.2043 | 5.95E-19 | 7.748273 | 6.83E-16 | 9.76E-17 | 9.57E-16 |
| GOTERM_BP_FAT | GO:0051301~cell division | 29 | 15.5914 | 1.36E-18 | 8.807094 | 1.56E-15 | 1.95E-16 | 2.18E-15 |
| GOTERM_BP_FAT | GO:0007049~cell cycle | 42 | 22.58065 | 1.18E-17 | 4.848911 | 1.36E-14 | 1.51E-15 | 1.90E-14 |
| GOTERM_BP_FAT | GO:0007346~regulation of mitotic cell cycle | 13 | 6.989247 | 1.19E-07 | 7.662252 | 1.37E-04 | 9.11E-06 | 1.91E-04 |
| GOTERM_BP_FAT | GO:0051726~regulation of cell cycle | 18 | 9.677419 | 1.52E-07 | 4.871931 | 1.74E-04 | 1.09E-05 | 2.44E-04 |
|  |  |  |  |  |  |  |  |  |
| Annotation Cluster 2 | Enrichment Score: 7.113489827104099 | | | |  |  |  |  |
| Category | Term | Count | % | PValue | Fold Enrichment | Bonferroni | Benjamini | FDR |
| GOTERM_BP_FAT | GO:0000226~microtubule cytoskeleton organization | 14 | 7.526882 | 8.83E-09 | 8.532324 | 1.01E-05 | 9.22E-07 | 1.42E-05 |
| GOTERM_BP_FAT | GO:0007017~microtubule-based process | 17 | 9.139785 | 2.05E-08 | 6.019841 | 2.35E-05 | 1.96E-06 | 3.29E-05 |
| GOTERM_BP_FAT | GO:0007051~spindle organization | 9 | 4.83871 | 2.89E-08 | 17.91788 | 3.31E-05 | 2.55E-06 | 4.64E-05 |
| GOTERM_BP_FAT | GO:0007010~cytoskeleton organization | 18 | 9.677419 | 6.73E-06 | 3.698645 | 0.0077 | 3.86E-04 | 0.010826 |
|  |  |  |  |  |  |  |  |  |
| Annotation Cluster 3 | Enrichment Score: 5.170932193629179 | | | |  |  |  |  |
| Category | Term | Count | % | PValue | Fold Enrichment | Bonferroni | Benjamini | FDR |
| GOTERM_BP_FAT | GO:0007059~chromosome segregation | 13 | 6.989247 | 8.13E-11 | 14.37855 | 9.33E-08 | 9.33E-09 | 1.31E-07 |
| GOTERM_BP_FAT | GO:0000070~mitotic sister chromatid segregation | 7 | 3.763441 | 2.49E-06 | 17.42016 | 0.002853 | 1.59E-04 | 0.004001 |
| GOTERM_BP_FAT | GO:0000819~sister chromatid segregation | 7 | 3.763441 | 2.94E-06 | 16.94935 | 0.003373 | 1.78E-04 | 0.004732 |
| GOTERM_BP_FAT | GO:0051276~chromosome organization | 19 | 10.21505 | 7.01E-06 | 3.509688 | 0.008015 | 3.83E-04 | 0.011269 |
| GOTERM_BP_FAT | GO:0030261~chromosome condensation | 4 | 2.150538 | 0.00257 | 14.3343 | 0.947855 | 0.066385 | 4.052245 |
| GOTERM_BP_FAT | GO:0007076~mitotic chromosome condensation | 3 | 1.612903 | 0.008793 | 20.67448 | 0.99996 | 0.165601 | 13.23678 |
|  |  |  |  |  |  |  |  |  |
| Annotation Cluster 4 | Enrichment Score: 3.4965069374147366 | | | |  |  |  |  |
| Category | Term | Count | % | PValue | Fold Enrichment | Bonferroni | Benjamini | FDR |
| GOTERM_BP_FAT | GO:0007051~spindle organization | 9 | 4.83871 | 2.89E-08 | 17.91788 | 3.31E-05 | 2.55E-06 | 4.64E-05 |
| GOTERM_BP_FAT | GO:0048015~phosphoinositide-mediated signaling | 8 | 4.301075 | 5.37E-05 | 8.144491 | 0.05976 | 0.002564 | 0.086261 |
| GOTERM_BP_FAT | GO:0019932~second-messenger-mediated signaling | 8 | 4.301075 | 0.015828 | 3.049852 | 1 | 0.259368 | 22.62479 |
| GOTERM_BP_FAT | GO:0007242~intracellular signaling cascade | 16 | 8.602151 | 0.421215 | 1.141266 | 1 | 0.978747 | 99.9848 |
|  |  |  |  |  |  |  |  |  |
| Annotation Cluster 5 | Enrichment Score: 3.0582624110089967 | | | |  |  |  |  |
| Category | Term | Count | % | PValue | Fold Enrichment | Bonferroni | Benjamini | FDR |
| GOTERM_BP_FAT | GO:0000075~cell cycle checkpoint | 11 | 5.913978 | 6.20E-08 | 10.82949 | 7.12E-05 | 5.08E-06 | 9.97E-05 |
| GOTERM_BP_FAT | GO:0007093~mitotic cell cycle checkpoint | 8 | 4.301075 | 4.13E-07 | 16.6678 | 4.74E-04 | 2.79E-05 | 6.64E-04 |
| GOTERM_BP_FAT | GO:0010564~regulation of cell cycle process | 9 | 4.83871 | 3.87E-05 | 7.072848 | 0.043433 | 0.001929 | 0.062169 |
| GOTERM_BP_FAT | GO:0030071~regulation of mitotic metaphase/anaphase transition | 5 | 2.688172 | 9.09E-05 | 20.36123 | 0.099126 | 0.004007 | 0.14609 |
| GOTERM_BP_FAT | GO:0007094~mitotic cell cycle spindle assembly checkpoint | 4 | 2.150538 | 2.07E-04 | 32.57797 | 0.211124 | 0.008144 | 0.331569 |
| GOTERM_BP_FAT | GO:0045841~negative regulation of mitotic metaphase/anaphase transition | 4 | 2.150538 | 2.07E-04 | 32.57797 | 0.211124 | 0.008144 | 0.331569 |
| GOTERM_BP_FAT | GO:0051784~negative regulation of nuclear division | 4 | 2.150538 | 2.73E-04 | 29.86313 | 0.269217 | 0.010066 | 0.438282 |
| GOTERM_BP_FAT | GO:0031577~spindle checkpoint | 4 | 2.150538 | 2.73E-04 | 29.86313 | 0.269217 | 0.010066 | 0.438282 |
| GOTERM_BP_FAT | GO:0045839~negative regulation of mitosis | 4 | 2.150538 | 2.73E-04 | 29.86313 | 0.269217 | 0.010066 | 0.438282 |
| GOTERM_BP_FAT | GO:0010639~negative regulation of organelle organization | 7 | 3.763441 | 2.94E-04 | 7.647876 | 0.286757 | 0.010188 | 0.472151 |
| GOTERM_BP_FAT | GO:0007088~regulation of mitosis | 6 | 3.225806 | 3.80E-04 | 9.598865 | 0.353485 | 0.012384 | 0.608972 |
| GOTERM_BP_FAT | GO:0051783~regulation of nuclear division | 6 | 3.225806 | 3.80E-04 | 9.598865 | 0.353485 | 0.012384 | 0.608972 |
| GOTERM_BP_FAT | GO:0051129~negative regulation of cellular component organization | 8 | 4.301075 | 0.001024 | 5.04729 | 0.691547 | 0.029708 | 1.633734 |
| GOTERM_BP_FAT | GO:0010948~negative regulation of cell cycle process | 4 | 2.150538 | 0.00257 | 14.3343 | 0.947855 | 0.066385 | 4.052245 |
| GOTERM_BP_FAT | GO:0033043~regulation of organelle organization | 9 | 4.83871 | 0.002893 | 3.71569 | 0.964073 | 0.07281 | 4.551542 |
| GOTERM_BP_FAT | GO:0051640~organelle localization | 6 | 3.225806 | 0.003557 | 5.842787 | 0.983272 | 0.086894 | 5.56787 |
| GOTERM_BP_FAT | GO:0051656~establishment of organelle localization | 5 | 2.688172 | 0.007187 | 6.491986 | 0.999746 | 0.14986 | 10.94897 |
| GOTERM_BP_FAT | GO:0051785~positive regulation of nuclear division | 3 | 1.612903 | 0.031013 | 10.75073 | 1 | 0.403491 | 39.74002 |
| GOTERM_BP_FAT | GO:0045840~positive regulation of mitosis | 3 | 1.612903 | 0.031013 | 10.75073 | 1 | 0.403491 | 39.74002 |
| GOTERM_BP_FAT | GO:0045786~negative regulation of cell cycle | 4 | 2.150538 | 0.060992 | 4.424168 | 1 | 0.603953 | 63.64305 |
| GOTERM_BP_FAT | GO:0010638~positive regulation of organelle organization | 4 | 2.150538 | 0.064661 | 4.317562 | 1 | 0.616815 | 65.86052 |
| GOTERM_BP_FAT | GO:0045787~positive regulation of cell cycle | 3 | 1.612903 | 0.131588 | 4.715232 | 1 | 0.795654 | 89.65181 |
| GOTERM_BP_FAT | GO:0051130~positive regulation of cellular component organization | 5 | 2.688172 | 0.141468 | 2.474845 | 1 | 0.814317 | 91.39073 |
|  |  |  |  |  |  |  |  |  |
| Annotation Cluster 6 | Enrichment Score: 2.4654568358041664 | | | |  |  |  |  |
| Category | Term | Count | % | PValue | Fold Enrichment | Bonferroni | Benjamini | FDR |
| GOTERM_BP_FAT | GO:0007098~centrosome cycle | 4 | 2.150538 | 0.001137 | 18.86093 | 0.728969 | 0.03134 | 1.811748 |
| GOTERM_BP_FAT | GO:0051297~centrosome organization | 4 | 2.150538 | 0.005237 | 11.19868 | 0.997588 | 0.12281 | 8.094912 |
| GOTERM_BP_FAT | GO:0031023~microtubule organizing center organization | 4 | 2.150538 | 0.006745 | 10.23879 | 0.999578 | 0.146629 | 10.30992 |
|  |  |  |  |  |  |  |  |  |
| Annotation Cluster 7 | Enrichment Score: 1.6358540402297657 | | | |  |  |  |  |
| Category | Term | Count | % | PValue | Fold Enrichment | Bonferroni | Benjamini | FDR |
| GOTERM_BP_FAT | GO:0031145~anaphase-promoting complex-dependent proteasomal ubiquitin-dependent protein catabolic process | 7 | 3.763441 | 8.10E-05 | 9.64809 | 0.08882 | 0.003714 | 0.13018 |
| GOTERM_BP_FAT | GO:0051439~regulation of ubiquitin-protein ligase activity during mitotic cell cycle | 7 | 3.763441 | 1.33E-04 | 8.832758 | 0.141631 | 0.00564 | 0.213655 |
| GOTERM_BP_FAT | GO:0051438~regulation of ubiquitin-protein ligase activity | 7 | 3.763441 | 2.24E-04 | 8.040075 | 0.226659 | 0.008531 | 0.359326 |
| GOTERM_BP_FAT | GO:0051340~regulation of ligase activity | 7 | 3.763441 | 2.75E-04 | 7.742294 | 0.270982 | 0.009828 | 0.441655 |
| GOTERM_BP_FAT | GO:0031396~regulation of protein ubiquitination | 7 | 3.763441 | 8.51E-04 | 6.271258 | 0.623797 | 0.026076 | 1.359818 |
| GOTERM_BP_FAT | GO:0043161~proteasomal ubiquitin-dependent protein catabolic process | 7 | 3.763441 | 9.44E-04 | 6.148292 | 0.661959 | 0.028138 | 1.507472 |
| GOTERM_BP_FAT | GO:0010498~proteasomal protein catabolic process | 7 | 3.763441 | 9.44E-04 | 6.148292 | 0.661959 | 0.028138 | 1.507472 |
| GOTERM_BP_FAT | GO:0051436~negative regulation of ubiquitin-protein ligase activity during mitotic cell cycle | 5 | 2.688172 | 0.005823 | 6.891493 | 0.998774 | 0.132935 | 8.961747 |
| GOTERM_BP_FAT | GO:0051444~negative regulation of ubiquitin-protein ligase activity | 5 | 2.688172 | 0.006481 | 6.685776 | 0.999427 | 0.144019 | 9.925827 |
| GOTERM_BP_FAT | GO:0051352~negative regulation of ligase activity | 5 | 2.688172 | 0.006481 | 6.685776 | 0.999427 | 0.144019 | 9.925827 |
| GOTERM_BP_FAT | GO:0051437~positive regulation of ubiquitin-protein ligase activity during mitotic cell cycle | 5 | 2.688172 | 0.006828 | 6.587456 | 0.999616 | 0.145555 | 10.43002 |
| GOTERM_BP_FAT | GO:0051443~positive regulation of ubiquitin-protein ligase activity | 5 | 2.688172 | 0.007558 | 6.399243 | 0.999835 | 0.151534 | 11.48266 |
| GOTERM_BP_FAT | GO:0051351~positive regulation of ligase activity | 5 | 2.688172 | 0.008746 | 6.136261 | 0.999958 | 0.167533 | 13.17164 |
| GOTERM_BP_FAT | GO:0031397~negative regulation of protein ubiquitination | 5 | 2.688172 | 0.009168 | 6.053338 | 0.999974 | 0.16931 | 13.76368 |
| GOTERM_BP_FAT | GO:0031400~negative regulation of protein modification process | 6 | 3.225806 | 0.010423 | 4.517113 | 0.999994 | 0.187302 | 15.50378 |
| GOTERM_BP_FAT | GO:0031398~positive regulation of protein ubiquitination | 5 | 2.688172 | 0.014127 | 5.332703 | 1 | 0.241822 | 20.44697 |
| GOTERM_BP_FAT | GO:0032269~negative regulation of cellular protein metabolic process | 7 | 3.763441 | 0.015121 | 3.484032 | 1 | 0.252873 | 21.72651 |
| GOTERM_BP_FAT | GO:0031399~regulation of protein modification process | 9 | 4.83871 | 0.017067 | 2.733236 | 1 | 0.272943 | 24.17722 |
| GOTERM_BP_FAT | GO:0051248~negative regulation of protein metabolic process | 7 | 3.763441 | 0.017918 | 3.353614 | 1 | 0.280698 | 25.22572 |
| GOTERM_BP_FAT | GO:0006511~ubiquitin-dependent protein catabolic process | 7 | 3.763441 | 0.052733 | 2.591429 | 1 | 0.56848 | 58.14663 |
| GOTERM_BP_FAT | GO:0031401~positive regulation of protein modification process | 6 | 3.225806 | 0.057191 | 2.874526 | 1 | 0.589171 | 61.20339 |
| GOTERM_BP_FAT | GO:0010605~negative regulation of macromolecule metabolic process | 14 | 7.526882 | 0.064115 | 1.70879 | 1 | 0.618217 | 65.53888 |
| GOTERM_BP_FAT | GO:0044092~negative regulation of molecular function | 8 | 4.301075 | 0.078446 | 2.145854 | 1 | 0.663957 | 73.11052 |
| GOTERM_BP_FAT | GO:0032268~regulation of cellular protein metabolic process | 10 | 5.376344 | 0.081239 | 1.890072 | 1 | 0.66476 | 74.39123 |
| GOTERM_BP_FAT | GO:0043086~negative regulation of catalytic activity | 7 | 3.763441 | 0.088037 | 2.263992 | 1 | 0.687321 | 77.27347 |
| GOTERM_BP_FAT | GO:0032270~positive regulation of cellular protein metabolic process | 6 | 3.225806 | 0.117404 | 2.307023 | 1 | 0.765007 | 86.5729 |
| GOTERM_BP_FAT | GO:0051247~positive regulation of protein metabolic process | 6 | 3.225806 | 0.133398 | 2.212084 | 1 | 0.797249 | 89.99321 |
| GOTERM_BP_FAT | GO:0043085~positive regulation of catalytic activity | 9 | 4.83871 | 0.220279 | 1.550586 | 1 | 0.911141 | 98.16915 |
| GOTERM_BP_FAT | GO:0019941~modification-dependent protein catabolic process | 9 | 4.83871 | 0.304956 | 1.404712 | 1 | 0.950434 | 99.71163 |
| GOTERM_BP_FAT | GO:0043632~modification-dependent macromolecule catabolic process | 9 | 4.83871 | 0.304956 | 1.404712 | 1 | 0.950434 | 99.71163 |
| GOTERM_BP_FAT | GO:0044093~positive regulation of molecular function | 9 | 4.83871 | 0.32477 | 1.375946 | 1 | 0.95536 | 99.81887 |
| GOTERM_BP_FAT | GO:0051603~proteolysis involved in cellular protein catabolic process | 9 | 4.83871 | 0.34818 | 1.343841 | 1 | 0.96384 | 99.89729 |
| GOTERM_BP_FAT | GO:0010604~positive regulation of macromolecule metabolic process | 12 | 6.451613 | 0.352492 | 1.254461 | 1 | 0.964868 | 99.90768 |
| GOTERM_BP_FAT | GO:0044257~cellular protein catabolic process | 9 | 4.83871 | 0.353229 | 1.337155 | 1 | 0.964387 | 99.90936 |
| GOTERM_BP_FAT | GO:0030163~protein catabolic process | 9 | 4.83871 | 0.385392 | 1.29631 | 1 | 0.971542 | 99.96008 |
| GOTERM_BP_FAT | GO:0044265~cellular macromolecule catabolic process | 10 | 5.376344 | 0.412983 | 1.235716 | 1 | 0.977593 | 99.98092 |
| GOTERM_BP_FAT | GO:0009057~macromolecule catabolic process | 10 | 5.376344 | 0.501897 | 1.147111 | 1 | 0.989931 | 99.99864 |
| GOTERM_BP_FAT | GO:0006508~proteolysis | 13 | 6.989247 | 0.50584 | 1.104993 | 1 | 0.989661 | 99.9988 |
|  |  |  |  |  |  |  |  |  |
| Annotation Cluster 8 | Enrichment Score: 1.42127412419609 | | | |  |  |  |  |
| Category | Term | Count | % | PValue | Fold Enrichment | Bonferroni | Benjamini | FDR |
| GOTERM_BP_FAT | GO:0010639~negative regulation of organelle organization | 7 | 3.763441 | 2.94E-04 | 7.647876 | 0.286757 | 0.010188 | 0.472151 |
| GOTERM_BP_FAT | GO:0051129~negative regulation of cellular component organization | 8 | 4.301075 | 0.001024 | 5.04729 | 0.691547 | 0.029708 | 1.633734 |
| GOTERM_BP_FAT | GO:0070507~regulation of microtubule cytoskeleton organization | 3 | 1.612903 | 0.07886 | 6.399243 | 1 | 0.661732 | 73.30429 |
| GOTERM_BP_FAT | GO:0032886~regulation of microtubule-based process | 3 | 1.612903 | 0.102533 | 5.485066 | 1 | 0.725731 | 82.43494 |
| GOTERM_BP_FAT | GO:0051493~regulation of cytoskeleton organization | 4 | 2.150538 | 0.191818 | 2.634982 | 1 | 0.887289 | 96.74185 |
| GOTERM_BP_FAT | GO:0006461~protein complex assembly | 7 | 3.763441 | 0.49053 | 1.241833 | 1 | 0.989476 | 99.99804 |
| GOTERM_BP_FAT | GO:0070271~protein complex biogenesis | 7 | 3.763441 | 0.49053 | 1.241833 | 1 | 0.989476 | 99.99804 |
|  |  |  |  |  |  |  |  |  |
| Annotation Cluster 9 | Enrichment Score: 1.404342282705653 | | | |  |  |  |  |
| Category | Term | Count | % | PValue | Fold Enrichment | Bonferroni | Benjamini | FDR |
| GOTERM_BP_FAT | GO:0008202~steroid metabolic process | 8 | 4.301075 | 0.007293 | 3.548095 | 0.999776 | 0.149207 | 11.10156 |
| GOTERM_BP_FAT | GO:0008203~cholesterol metabolic process | 4 | 2.150538 | 0.082374 | 3.895191 | 1 | 0.665975 | 74.89531 |
| GOTERM_BP_FAT | GO:0016125~sterol metabolic process | 4 | 2.150538 | 0.10193 | 3.548095 | 1 | 0.727235 | 82.24425 |
|  |  |  |  |  |  |  |  |  |
| Annotation Cluster 10 | Enrichment Score: 1.2394177864715028 | | | |  |  |  |  |
| Category | Term | Count | % | PValue | Fold Enrichment | Bonferroni | Benjamini | FDR |
| GOTERM_BP_FAT | GO:0006259~DNA metabolic process | 17 | 9.139785 | 1.56E-04 | 3.009921 | 0.16389 | 0.006372 | 0.250366 |
| GOTERM_BP_FAT | GO:0006974~response to DNA damage stimulus | 11 | 5.913978 | 0.008671 | 2.642047 | 0.999955 | 0.169017 | 13.0658 |
| GOTERM_BP_FAT | GO:0033554~cellular response to stress | 13 | 6.989247 | 0.023598 | 2.057707 | 1 | 0.344113 | 31.88283 |
| GOTERM_BP_FAT | GO:0006302~double-strand break repair | 4 | 2.150538 | 0.031409 | 5.779962 | 1 | 0.403093 | 40.1348 |
| GOTERM_BP_FAT | GO:0006281~DNA repair | 8 | 4.301075 | 0.038937 | 2.523645 | 1 | 0.469134 | 47.19326 |
| GOTERM_BP_FAT | GO:0019953~sexual reproduction | 10 | 5.376344 | 0.068794 | 1.956101 | 1 | 0.631329 | 68.20713 |
| GOTERM_BP_FAT | GO:0003006~reproductive developmental process | 7 | 3.763441 | 0.071617 | 2.39361 | 1 | 0.63781 | 69.72182 |
| GOTERM_BP_FAT | GO:0007276~gamete generation | 9 | 4.83871 | 0.07292 | 2.041278 | 1 | 0.640341 | 70.39758 |
| GOTERM_BP_FAT | GO:0006310~DNA recombination | 4 | 2.150538 | 0.111164 | 3.41293 | 1 | 0.748532 | 84.96242 |
| GOTERM_BP_FAT | GO:0048609~reproductive process in a multicellular organism | 9 | 4.83871 | 0.173672 | 1.655656 | 1 | 0.865896 | 95.34397 |
| GOTERM_BP_FAT | GO:0032504~multicellular organism reproduction | 9 | 4.83871 | 0.173672 | 1.655656 | 1 | 0.865896 | 95.34397 |
| GOTERM_BP_FAT | GO:0048232~male gamete generation | 6 | 3.225806 | 0.256724 | 1.745248 | 1 | 0.933006 | 99.15197 |
| GOTERM_BP_FAT | GO:0007283~spermatogenesis | 6 | 3.225806 | 0.256724 | 1.745248 | 1 | 0.933006 | 99.15197 |
| GOTERM_BP_FAT | GO:0048610~reproductive cellular process | 4 | 2.150538 | 0.268091 | 2.212084 | 1 | 0.93646 | 99.33809 |
| GOTERM_BP_FAT | GO:0007281~germ cell development | 3 | 1.612903 | 0.30864 | 2.661071 | 1 | 0.950467 | 99.73525 |
|  |  |  |  |  |  |  |  |  |
| Annotation Cluster 11 | Enrichment Score: 1.119584546735314 | | | |  |  |  |  |
| Category | Term | Count | % | PValue | Fold Enrichment | Bonferroni | Benjamini | FDR |
| GOTERM_BP_FAT | GO:0031570~DNA integrity checkpoint | 4 | 2.150538 | 0.019874 | 6.891493 | 1 | 0.302374 | 27.58347 |
| GOTERM_BP_FAT | GO:0000077~DNA damage checkpoint | 3 | 1.612903 | 0.099041 | 5.599338 | 1 | 0.720216 | 81.30316 |
| GOTERM_BP_FAT | GO:0042770~DNA damage response, signal transduction | 3 | 1.612903 | 0.222411 | 3.359603 | 1 | 0.91168 | 98.24801 |
|  |  |  |  |  |  |  |  |  |
| Annotation Cluster 12 | Enrichment Score: 0.9362395936342894 | | | |  |  |  |  |
| Category | Term | Count | % | PValue | Fold Enrichment | Bonferroni | Benjamini | FDR |
| GOTERM_BP_FAT | GO:0008202~steroid metabolic process | 8 | 4.301075 | 0.007293 | 3.548095 | 0.999776 | 0.149207 | 11.10156 |
| GOTERM_BP_FAT | GO:0006694~steroid biosynthetic process | 3 | 1.612903 | 0.242893 | 3.161979 | 1 | 0.925504 | 98.85937 |
| GOTERM_BP_FAT | GO:0008610~lipid biosynthetic process | 3 | 1.612903 | 0.876977 | 0.8321 | 1 | 0.999961 | 100 |
|  |  |  |  |  |  |  |  |  |
| Annotation Cluster 13 | Enrichment Score: 0.8952616364557502 | | | |  |  |  |  |
| Category | Term | Count | % | PValue | Fold Enrichment | Bonferroni | Benjamini | FDR |
| GOTERM_BP_FAT | GO:0016053~organic acid biosynthetic process | 5 | 2.688172 | 0.093784 | 2.889981 | 1 | 0.703474 | 79.47002 |
| GOTERM_BP_FAT | GO:0046394~carboxylic acid biosynthetic process | 5 | 2.688172 | 0.093784 | 2.889981 | 1 | 0.703474 | 79.47002 |
| GOTERM_BP_FAT | GO:0008652~cellular amino acid biosynthetic process | 3 | 1.612903 | 0.109618 | 5.269965 | 1 | 0.746933 | 84.53632 |
| GOTERM_BP_FAT | GO:0044271~nitrogen compound biosynthetic process | 7 | 3.763441 | 0.152928 | 1.929618 | 1 | 0.837097 | 93.06356 |
| GOTERM_BP_FAT | GO:0009309~amine biosynthetic process | 3 | 1.612903 | 0.226497 | 3.318126 | 1 | 0.914306 | 98.39032 |
|  |  |  |  |  |  |  |  |  |
| Annotation Cluster 14 | Enrichment Score: 0.7762699703426227 | | | |  |  |  |  |
| Category | Term | Count | % | PValue | Fold Enrichment | Bonferroni | Benjamini | FDR |
| GOTERM_BP_FAT | GO:0043542~endothelial cell migration | 3 | 1.612903 | 0.0244 | 12.21674 | 1 | 0.349281 | 32.77727 |
| GOTERM_BP_FAT | GO:0016477~cell migration | 6 | 3.225806 | 0.192373 | 1.947596 | 1 | 0.885889 | 96.77763 |
| GOTERM_BP_FAT | GO:0051674~localization of cell | 6 | 3.225806 | 0.254632 | 1.750933 | 1 | 0.932725 | 99.11278 |
| GOTERM_BP_FAT | GO:0048870~cell motility | 6 | 3.225806 | 0.254632 | 1.750933 | 1 | 0.932725 | 99.11278 |
| GOTERM_BP_FAT | GO:0006928~cell motion | 7 | 3.763441 | 0.431805 | 1.320265 | 1 | 0.980416 | 99.9887 |
|  |  |  |  |  |  |  |  |  |
| Annotation Cluster 15 | Enrichment Score: 0.6219382999282226 | | | |  |  |  |  |
| Category | Term | Count | % | PValue | Fold Enrichment | Bonferroni | Benjamini | FDR |
| GOTERM_BP_FAT | GO:0045664~regulation of neuron differentiation | 4 | 2.150538 | 0.183435 | 2.694418 | 1 | 0.877036 | 96.15385 |
| GOTERM_BP_FAT | GO:0060284~regulation of cell development | 5 | 2.688172 | 0.19323 | 2.185107 | 1 | 0.884932 | 96.83214 |
| GOTERM_BP_FAT | GO:0045596~negative regulation of cell differentiation | 5 | 2.688172 | 0.218483 | 2.073829 | 1 | 0.910974 | 98.10018 |
| GOTERM_BP_FAT | GO:0050767~regulation of neurogenesis | 4 | 2.150538 | 0.27973 | 2.158781 | 1 | 0.938598 | 99.48846 |
| GOTERM_BP_FAT | GO:0051960~regulation of nervous system development | 4 | 2.150538 | 0.358588 | 1.866446 | 1 | 0.965055 | 99.92071 |
|  |  |  |  |  |  |  |  |  |
| Annotation Cluster 16 | Enrichment Score: 0.6068691940887023 | | | |  |  |  |  |
| Category | Term | Count | % | PValue | Fold Enrichment | Bonferroni | Benjamini | FDR |
| GOTERM_BP_FAT | GO:0006323~DNA packaging | 7 | 3.763441 | 0.001916 | 5.36005 | 0.889322 | 0.051058 | 3.035613 |
| GOTERM_BP_FAT | GO:0065004~protein-DNA complex assembly | 4 | 2.150538 | 0.080312 | 3.937996 | 1 | 0.664513 | 73.97256 |
| GOTERM_BP_FAT | GO:0006334~nucleosome assembly | 3 | 1.612903 | 0.238788 | 3.199622 | 1 | 0.923266 | 98.75577 |
| GOTERM_BP_FAT | GO:0031497~chromatin assembly | 3 | 1.612903 | 0.251112 | 3.08929 | 1 | 0.931239 | 99.04295 |
| GOTERM_BP_FAT | GO:0034728~nucleosome organization | 3 | 1.612903 | 0.2758 | 2.889981 | 1 | 0.940859 | 99.4417 |
| GOTERM_BP_FAT | GO:0034622~cellular macromolecular complex assembly | 6 | 3.225806 | 0.277858 | 1.690366 | 1 | 0.939787 | 99.46667 |
| GOTERM_BP_FAT | GO:0034621~cellular macromolecular complex subunit organization | 6 | 3.225806 | 0.362849 | 1.505704 | 1 | 0.965269 | 99.92876 |
| GOTERM_BP_FAT | GO:0006325~chromatin organization | 6 | 3.225806 | 0.409179 | 1.422054 | 1 | 0.977619 | 99.97884 |
| GOTERM_BP_FAT | GO:0006333~chromatin assembly or disassembly | 3 | 1.612903 | 0.412423 | 2.116285 | 1 | 0.977969 | 99.98063 |
| GOTERM_BP_FAT | GO:0065003~macromolecular complex assembly | 9 | 4.83871 | 0.458424 | 1.212488 | 1 | 0.984864 | 99.99478 |
| GOTERM_BP_FAT | GO:0006461~protein complex assembly | 7 | 3.763441 | 0.49053 | 1.241833 | 1 | 0.989476 | 99.99804 |
| GOTERM_BP_FAT | GO:0070271~protein complex biogenesis | 7 | 3.763441 | 0.49053 | 1.241833 | 1 | 0.989476 | 99.99804 |
| GOTERM_BP_FAT | GO:0043933~macromolecular complex subunit organization | 9 | 4.83871 | 0.533121 | 1.13564 | 1 | 0.992022 | 99.99952 |
| GOTERM_BP_FAT | GO:0043623~cellular protein complex assembly | 3 | 1.612903 | 0.539732 | 1.659063 | 1 | 0.992309 | 99.99962 |
| GOTERM_BP_FAT | GO:0051259~protein oligomerization | 3 | 1.612903 | 0.574135 | 1.544645 | 1 | 0.994245 | 99.99989 |
|  |  |  |  |  |  |  |  |  |
| Annotation Cluster 17 | Enrichment Score: 0.5630782512002441 | | | |  |  |  |  |
| Category | Term | Count | % | PValue | Fold Enrichment | Bonferroni | Benjamini | FDR |
| GOTERM_BP_FAT | GO:0051329~interphase of mitotic cell cycle | 5 | 2.688172 | 0.027589 | 4.349 | 1 | 0.376441 | 36.22422 |
| GOTERM_BP_FAT | GO:0051325~interphase | 5 | 2.688172 | 0.030229 | 4.225915 | 1 | 0.399928 | 38.95213 |
| GOTERM_BP_FAT | GO:0010605~negative regulation of macromolecule metabolic process | 14 | 7.526882 | 0.064115 | 1.70879 | 1 | 0.618217 | 65.53888 |
| GOTERM_BP_FAT | GO:0051053~negative regulation of DNA metabolic process | 3 | 1.612903 | 0.069325 | 6.891493 | 1 | 0.629799 | 68.49704 |
| GOTERM_BP_FAT | GO:0007568~aging | 3 | 1.612903 | 0.345225 | 2.443347 | 1 | 0.963376 | 99.88954 |
| GOTERM_BP_FAT | GO:0051052~regulation of DNA metabolic process | 3 | 1.612903 | 0.361295 | 2.357616 | 1 | 0.965397 | 99.92592 |
| GOTERM_BP_FAT | GO:0045934~negative regulation of nucleobase, nucleoside, nucleotide and nucleic acid metabolic process | 7 | 3.763441 | 0.50398 | 1.224855 | 1 | 0.989943 | 99.99873 |
| GOTERM_BP_FAT | GO:0051172~negative regulation of nitrogen compound metabolic process | 7 | 3.763441 | 0.517307 | 1.208335 | 1 | 0.99064 | 99.99918 |
| GOTERM_BP_FAT | GO:0010558~negative regulation of macromolecule biosynthetic process | 6 | 3.225806 | 0.730652 | 0.982699 | 1 | 0.999231 | 100 |
| GOTERM_BP_FAT | GO:0031327~negative regulation of cellular biosynthetic process | 6 | 3.225806 | 0.750716 | 0.958175 | 1 | 0.99944 | 100 |
| GOTERM_BP_FAT | GO:0009890~negative regulation of biosynthetic process | 6 | 3.225806 | 0.767026 | 0.938109 | 1 | 0.99955 | 100 |
| GOTERM_BP_FAT | GO:0016481~negative regulation of transcription | 3 | 1.612903 | 0.96533 | 0.585552 | 1 | 1 | 100 |
| GOTERM_BP_FAT | GO:0010629~negative regulation of gene expression | 3 | 1.612903 | 0.977628 | 0.53327 | 1 | 1 | 100 |
|  |  |  |  |  |  |  |  |  |
| Annotation Cluster 18 | Enrichment Score: 0.5340343355719743 | | | |  |  |  |  |
| Category | Term | Count | % | PValue | Fold Enrichment | Bonferroni | Benjamini | FDR |
| GOTERM_BP_FAT | GO:0003006~reproductive developmental process | 7 | 3.763441 | 0.071617 | 2.39361 | 1 | 0.63781 | 69.72182 |
| GOTERM_BP_FAT | GO:0008406~gonad development | 3 | 1.612903 | 0.353277 | 2.399716 | 1 | 0.963612 | 99.90947 |
| GOTERM_BP_FAT | GO:0048608~reproductive structure development | 3 | 1.612903 | 0.408558 | 2.133081 | 1 | 0.977983 | 99.97848 |
| GOTERM_BP_FAT | GO:0045137~development of primary sexual characteristics | 3 | 1.612903 | 0.412423 | 2.116285 | 1 | 0.977969 | 99.98063 |
| GOTERM_BP_FAT | GO:0007548~sex differentiation | 3 | 1.612903 | 0.5013 | 1.779922 | 1 | 0.990117 | 99.99861 |
|  |  |  |  |  |  |  |  |  |
| Annotation Cluster 19 | Enrichment Score: 0.5242469640787654 | | | |  |  |  |  |
| Category | Term | Count | % | PValue | Fold Enrichment | Bonferroni | Benjamini | FDR |
| GOTERM_BP_FAT | GO:0007126~meiosis | 3 | 1.612903 | 0.296348 | 2.742533 | 1 | 0.947407 | 99.64853 |
| GOTERM_BP_FAT | GO:0051327~M phase of meiotic cell cycle | 3 | 1.612903 | 0.296348 | 2.742533 | 1 | 0.947407 | 99.64853 |
| GOTERM_BP_FAT | GO:0051321~meiotic cell cycle | 3 | 1.612903 | 0.304547 | 2.687682 | 1 | 0.951264 | 99.70889 |
|  |  |  |  |  |  |  |  |  |
| Annotation Cluster 20 | Enrichment Score: 0.49849669493596843 | | | | |  |  |  |
| Category | Term | Count | % | PValue | Fold Enrichment | Bonferroni | Benjamini | FDR |
| GOTERM_BP_FAT | GO:0009719~response to endogenous stimulus | 8 | 4.301075 | 0.1637 | 1.769667 | 1 | 0.850467 | 94.35359 |
| GOTERM_BP_FAT | GO:0010033~response to organic substance | 11 | 5.913978 | 0.278455 | 1.366829 | 1 | 0.938945 | 99.47371 |
| GOTERM_BP_FAT | GO:0048545~response to steroid hormone stimulus | 4 | 2.150538 | 0.358588 | 1.866446 | 1 | 0.965055 | 99.92071 |
| GOTERM_BP_FAT | GO:0009725~response to hormone stimulus | 6 | 3.225806 | 0.384927 | 1.464677 | 1 | 0.972028 | 99.95959 |
| GOTERM_BP_FAT | GO:0043434~response to peptide hormone stimulus | 3 | 1.612903 | 0.511374 | 1.745248 | 1 | 0.990136 | 99.999 |
|  |  |  |  |  |  |  |  |  |
| Annotation Cluster 21 | Enrichment Score: 0.41586347027170345 | | | | |  |  |  |
| Category | Term | Count | % | PValue | Fold Enrichment | Bonferroni | Benjamini | FDR |
| GOTERM_BP_FAT | GO:0048534~hemopoietic or lymphoid organ development | 6 | 3.225806 | 0.162675 | 2.067448 | 1 | 0.851156 | 94.24132 |
| GOTERM_BP_FAT | GO:0002520~immune system development | 6 | 3.225806 | 0.192373 | 1.947596 | 1 | 0.885889 | 96.77763 |
| GOTERM_BP_FAT | GO:0030097~hemopoiesis | 5 | 2.688172 | 0.266417 | 1.898081 | 1 | 0.937878 | 99.31333 |
| GOTERM_BP_FAT | GO:0030099~myeloid cell differentiation | 3 | 1.612903 | 0.2758 | 2.889981 | 1 | 0.940859 | 99.4417 |
| GOTERM_BP_FAT | GO:0048872~homeostasis of number of cells | 3 | 1.612903 | 0.304547 | 2.687682 | 1 | 0.951264 | 99.70889 |
| GOTERM_BP_FAT | GO:0043009~chordate embryonic development | 6 | 3.225806 | 0.305838 | 1.623977 | 1 | 0.949884 | 99.71746 |
| GOTERM_BP_FAT | GO:0001701~in utero embryonic development | 4 | 2.150538 | 0.310153 | 2.036123 | 1 | 0.950296 | 99.74441 |
| GOTERM_BP_FAT | GO:0009792~embryonic development ending in birth or egg hatching | 6 | 3.225806 | 0.312359 | 1.60939 | 1 | 0.949486 | 99.75724 |
| GOTERM_BP_FAT | GO:0002521~leukocyte differentiation | 3 | 1.612903 | 0.427758 | 2.051666 | 1 | 0.979906 | 99.98734 |
| GOTERM_BP_FAT | GO:0045321~leukocyte activation | 4 | 2.150538 | 0.504721 | 1.480817 | 1 | 0.989777 | 99.99876 |
| GOTERM_BP_FAT | GO:0001775~cell activation | 4 | 2.150538 | 0.620426 | 1.248633 | 1 | 0.996565 | 99.99998 |
| GOTERM_BP_FAT | GO:0046649~lymphocyte activation | 3 | 1.612903 | 0.650815 | 1.350594 | 1 | 0.997394 | 100 |
| GOTERM_BP_FAT | GO:0042592~homeostatic process | 7 | 3.763441 | 0.846005 | 0.835054 | 1 | 0.999912 | 100 |
| GOTERM_BP_FAT | GO:0006955~immune response | 4 | 2.150538 | 0.984335 | 0.519359 | 1 | 1 | 100 |
|  |  |  |  |  |  |  |  |  |
| Annotation Cluster 22 | Enrichment Score: 0.31675180508920076 | | | | |  |  |  |
| Category | Term | Count | % | PValue | Fold Enrichment | Bonferroni | Benjamini | FDR |
| GOTERM_BP_FAT | GO:0048232~male gamete generation | 6 | 3.225806 | 0.256724 | 1.745248 | 1 | 0.933006 | 99.15197 |
| GOTERM_BP_FAT | GO:0007283~spermatogenesis | 6 | 3.225806 | 0.256724 | 1.745248 | 1 | 0.933006 | 99.15197 |
| GOTERM_BP_FAT | GO:0032774~RNA biosynthetic process | 4 | 2.150538 | 0.641267 | 1.210668 | 1 | 0.997218 | 99.99999 |
| GOTERM_BP_FAT | GO:0006366~transcription from RNA polymerase II promoter | 3 | 1.612903 | 0.735977 | 1.148582 | 1 | 0.999287 | 100 |
| GOTERM_BP_FAT | GO:0006351~transcription, DNA-dependent | 3 | 1.612903 | 0.838314 | 0.920439 | 1 | 0.999896 | 100 |
|  |  |  |  |  |  |  |  |  |
| Annotation Cluster 23 | Enrichment Score: 0.2680275605932241 | | | |  |  |  |  |
| Category | Term | Count | % | PValue | Fold Enrichment | Bonferroni | Benjamini | FDR |
| GOTERM_BP_FAT | GO:0000079~regulation of cyclin-dependent protein kinase activity | 3 | 1.612903 | 0.120477 | 4.977189 | 1 | 0.770934 | 87.30494 |
| GOTERM_BP_FAT | GO:0008285~negative regulation of cell proliferation | 6 | 3.225806 | 0.371679 | 1.489021 | 1 | 0.967993 | 99.94308 |
| GOTERM_BP_FAT | GO:0051338~regulation of transferase activity | 5 | 2.688172 | 0.594662 | 1.204159 | 1 | 0.995222 | 99.99995 |
| GOTERM_BP_FAT | GO:0045859~regulation of protein kinase activity | 4 | 2.150538 | 0.740551 | 1.038718 | 1 | 0.999329 | 100 |
| GOTERM_BP_FAT | GO:0043549~regulation of kinase activity | 4 | 2.150538 | 0.761276 | 1.003803 | 1 | 0.999523 | 100 |
| GOTERM_BP_FAT | GO:0042325~regulation of phosphorylation | 5 | 2.688172 | 0.764187 | 0.96126 | 1 | 0.999537 | 100 |
| GOTERM_BP_FAT | GO:0051174~regulation of phosphorus metabolic process | 5 | 2.688172 | 0.790808 | 0.923602 | 1 | 0.999694 | 100 |
| GOTERM_BP_FAT | GO:0019220~regulation of phosphate metabolic process | 5 | 2.688172 | 0.790808 | 0.923602 | 1 | 0.999694 | 100 |
|  |  |  |  |  |  |  |  |  |
| Annotation Cluster 24 | Enrichment Score: 0.24782841124637203 | | | | |  |  |  |
| Category | Term | Count | % | PValue | Fold Enrichment | Bonferroni | Benjamini | FDR |
| GOTERM_BP_FAT | GO:0006468~protein amino acid phosphorylation | 9 | 4.83871 | 0.461799 | 1.208853 | 1 | 0.985129 | 99.99528 |
| GOTERM_BP_FAT | GO:0016310~phosphorylation | 10 | 5.376344 | 0.531343 | 1.119868 | 1 | 0.992042 | 99.99949 |
| GOTERM_BP_FAT | GO:0006793~phosphorus metabolic process | 11 | 5.913978 | 0.644806 | 1.01283 | 1 | 0.997293 | 99.99999 |
| GOTERM_BP_FAT | GO:0006796~phosphate metabolic process | 11 | 5.913978 | 0.644806 | 1.01283 | 1 | 0.997293 | 99.99999 |
|  |  |  |  |  |  |  |  |  |
| Annotation Cluster 25 | Enrichment Score: 0.16711179945900934 | | | | |  |  |  |
| Category | Term | Count | % | PValue | Fold Enrichment | Bonferroni | Benjamini | FDR |
| GOTERM_BP_FAT | GO:0042981~regulation of apoptosis | 10 | 5.376344 | 0.537469 | 1.114296 | 1 | 0.992277 | 99.99959 |
| GOTERM_BP_FAT | GO:0043067~regulation of programmed cell death | 10 | 5.376344 | 0.549638 | 1.103318 | 1 | 0.993105 | 99.99973 |
| GOTERM_BP_FAT | GO:0010941~regulation of cell death | 10 | 5.376344 | 0.55417 | 1.099256 | 1 | 0.993348 | 99.99977 |
| GOTERM_BP_FAT | GO:0043066~negative regulation of apoptosis | 5 | 2.688172 | 0.555234 | 1.265387 | 1 | 0.993266 | 99.99978 |
| GOTERM_BP_FAT | GO:0043069~negative regulation of programmed cell death | 5 | 2.688172 | 0.566388 | 1.247763 | 1 | 0.993919 | 99.99985 |
| GOTERM_BP_FAT | GO:0060548~negative regulation of cell death | 5 | 2.688172 | 0.5686 | 1.244297 | 1 | 0.993943 | 99.99987 |
| GOTERM_BP_FAT | GO:0006916~anti-apoptosis | 3 | 1.612903 | 0.669422 | 1.3047 | 1 | 0.998029 | 100 |
| GOTERM_BP_FAT | GO:0012501~programmed cell death | 7 | 3.763441 | 0.676533 | 1.026393 | 1 | 0.998201 | 100 |
| GOTERM_BP_FAT | GO:0006915~apoptosis | 6 | 3.225806 | 0.803119 | 0.892918 | 1 | 0.999767 | 100 |
| GOTERM_BP_FAT | GO:0008219~cell death | 7 | 3.763441 | 0.815206 | 0.87222 | 1 | 0.999812 | 100 |
| GOTERM_BP_FAT | GO:0016265~death | 7 | 3.763441 | 0.820312 | 0.866196 | 1 | 0.99983 | 100 |
| GOTERM_BP_FAT | GO:0043065~positive regulation of apoptosis | 4 | 2.150538 | 0.860143 | 0.83339 | 1 | 0.999943 | 100 |
| GOTERM_BP_FAT | GO:0043068~positive regulation of programmed cell death | 4 | 2.150538 | 0.863311 | 0.827616 | 1 | 0.999947 | 100 |
| GOTERM_BP_FAT | GO:0010942~positive regulation of cell death | 4 | 2.150538 | 0.865389 | 0.823811 | 1 | 0.999949 | 100 |
|  |  |  |  |  |  |  |  |  |
| Annotation Cluster 26 | Enrichment Score: 0.1509622154973382 | | | |  |  |  |  |
| Category | Term | Count | % | PValue | Fold Enrichment | Bonferroni | Benjamini | FDR |
| GOTERM_BP_FAT | GO:0000902~cell morphogenesis | 5 | 2.688172 | 0.559714 | 1.258278 | 1 | 0.993501 | 99.99981 |
| GOTERM_BP_FAT | GO:0030030~cell projection organization | 5 | 2.688172 | 0.586076 | 1.217247 | 1 | 0.995017 | 99.99993 |
| GOTERM_BP_FAT | GO:0032989~cellular component morphogenesis | 5 | 2.688172 | 0.645889 | 1.12833 | 1 | 0.99726 | 99.99999 |
| GOTERM_BP_FAT | GO:0048666~neuron development | 4 | 2.150538 | 0.72967 | 1.057102 | 1 | 0.999242 | 100 |
| GOTERM_BP_FAT | GO:0048858~cell projection morphogenesis | 3 | 1.612903 | 0.758852 | 1.097013 | 1 | 0.999514 | 100 |
| GOTERM_BP_FAT | GO:0031175~neuron projection development | 3 | 1.612903 | 0.780005 | 1.049876 | 1 | 0.99963 | 100 |
| GOTERM_BP_FAT | GO:0032990~cell part morphogenesis | 3 | 1.612903 | 0.780005 | 1.049876 | 1 | 0.99963 | 100 |
| GOTERM_BP_FAT | GO:0030182~neuron differentiation | 4 | 2.150538 | 0.868453 | 0.818168 | 1 | 0.99995 | 100 |
|  |  |  |  |  |  |  |  |  |
| Annotation Cluster 27 | Enrichment Score: 0.12766853383465948 | | | | |  |  |  |
| Category | Term | Count | % | PValue | Fold Enrichment | Bonferroni | Benjamini | FDR |
| GOTERM_BP_FAT | GO:0001525~angiogenesis | 3 | 1.612903 | 0.492074 | 1.816001 | 1 | 0.98941 | 99.99814 |
| GOTERM_BP_FAT | GO:0048514~blood vessel morphogenesis | 3 | 1.612903 | 0.68222 | 1.273783 | 1 | 0.998319 | 100 |
| GOTERM_BP_FAT | GO:0001568~blood vessel development | 3 | 1.612903 | 0.758852 | 1.097013 | 1 | 0.999514 | 100 |
| GOTERM_BP_FAT | GO:0001944~vasculature development | 3 | 1.612903 | 0.770598 | 1.07079 | 1 | 0.999571 | 100 |
| GOTERM_BP_FAT | GO:0016337~cell-cell adhesion | 3 | 1.612903 | 0.814349 | 0.973798 | 1 | 0.999814 | 100 |
| GOTERM_BP_FAT | GO:0007155~cell adhesion | 6 | 3.225806 | 0.893541 | 0.767909 | 1 | 0.999975 | 100 |
| GOTERM_BP_FAT | GO:0022610~biological adhesion | 6 | 3.225806 | 0.894243 | 0.766814 | 1 | 0.999974 | 100 |
|  |  |  |  |  |  |  |  |  |
| Annotation Cluster 28 | Enrichment Score: 0.09281763027727453 | | | | |  |  |  |
| Category | Term | Count | % | PValue | Fold Enrichment | Bonferroni | Benjamini | FDR |
| GOTERM_BP_FAT | GO:0006811~ion transport | 9 | 4.83871 | 0.62325 | 1.049876 | 1 | 0.996616 | 99.99998 |
| GOTERM_BP_FAT | GO:0006812~cation transport | 5 | 2.688172 | 0.867007 | 0.810031 | 1 | 0.99995 | 100 |
| GOTERM_BP_FAT | GO:0055085~transmembrane transport | 5 | 2.688172 | 0.881056 | 0.787253 | 1 | 0.999964 | 100 |
| GOTERM_BP_FAT | GO:0030001~metal ion transport | 4 | 2.150538 | 0.89339 | 0.770662 | 1 | 0.999976 | 100 |
|  |  |  |  |  |  |  |  |  |
| Annotation Cluster 29 | Enrichment Score: 0.06899183315198419 | | | | |  |  |  |
| Category | Term | Count | % | PValue | Fold Enrichment | Bonferroni | Benjamini | FDR |
| GOTERM_BP_FAT | GO:0010604~positive regulation of macromolecule metabolic process | 12 | 6.451613 | 0.352492 | 1.254461 | 1 | 0.964868 | 99.90768 |
| GOTERM_BP_FAT | GO:0010628~positive regulation of gene expression | 6 | 3.225806 | 0.777448 | 0.925192 | 1 | 0.99962 | 100 |
| GOTERM_BP_FAT | GO:0045944~positive regulation of transcription from RNA polymerase II promoter | 4 | 2.150538 | 0.783756 | 0.965923 | 1 | 0.999649 | 100 |
| GOTERM_BP_FAT | GO:0045941~positive regulation of transcription | 5 | 2.688172 | 0.876809 | 0.794232 | 1 | 0.999962 | 100 |
| GOTERM_BP_FAT | GO:0031328~positive regulation of cellular biosynthetic process | 6 | 3.225806 | 0.882534 | 0.784725 | 1 | 0.999964 | 100 |
| GOTERM_BP_FAT | GO:0009891~positive regulation of biosynthetic process | 6 | 3.225806 | 0.889974 | 0.773434 | 1 | 0.999973 | 100 |
| GOTERM_BP_FAT | GO:0045893~positive regulation of transcription, DNA-dependent | 4 | 2.150538 | 0.903057 | 0.751274 | 1 | 0.999982 | 100 |
| GOTERM_BP_FAT | GO:0051254~positive regulation of RNA metabolic process | 4 | 2.150538 | 0.906101 | 0.745026 | 1 | 0.999983 | 100 |
| GOTERM_BP_FAT | GO:0045935~positive regulation of nucleobase, nucleoside, nucleotide and nucleic acid metabolic process | 5 | 2.688172 | 0.92003 | 0.717864 | 1 | 0.999992 | 100 |
| GOTERM_BP_FAT | GO:0051173~positive regulation of nitrogen compound metabolic process | 5 | 2.688172 | 0.931108 | 0.69557 | 1 | 0.999996 | 100 |
| GOTERM_BP_FAT | GO:0010557~positive regulation of macromolecule biosynthetic process | 5 | 2.688172 | 0.936115 | 0.684934 | 1 | 0.999997 | 100 |
| GOTERM_BP_FAT | GO:0006357~regulation of transcription from RNA polymerase II promoter | 5 | 2.688172 | 0.963788 | 0.616158 | 1 | 1 | 100 |
| GOTERM_BP_FAT | GO:0051252~regulation of RNA metabolic process | 12 | 6.451613 | 0.993438 | 0.59298 | 1 | 1 | 100 |
| GOTERM_BP_FAT | GO:0006355~regulation of transcription, DNA-dependent | 11 | 5.913978 | 0.996263 | 0.555828 | 1 | 1 | 100 |
| GOTERM_BP_FAT | GO:0045449~regulation of transcription | 18 | 9.677419 | 0.996756 | 0.619996 | 1 | 1 | 100 |
|  |  |  |  |  |  |  |  |  |
| Annotation Cluster 30 | Enrichment Score: 0.0442049909859601 | | | |  |  |  |  |
| Category | Term | Count | % | PValue | Fold Enrichment | Bonferroni | Benjamini | FDR |
| GOTERM_BP_FAT | GO:0009611~response to wounding | 5 | 2.688172 | 0.844363 | 0.845183 | 1 | 0.999911 | 100 |
| GOTERM_BP_FAT | GO:0006954~inflammatory response | 3 | 1.612903 | 0.879155 | 0.826979 | 1 | 0.999963 | 100 |
| GOTERM_BP_FAT | GO:0006952~defense response | 3 | 1.612903 | 0.992637 | 0.437021 | 1 | 1 | 100 |
|  |  |  |  |  |  |  |  |  |
| Annotation Cluster 31 | Enrichment Score: 0.018019364759134012 | | | | |  |  |  |
| Category | Term | Count | % | PValue | Fold Enrichment | Bonferroni | Benjamini | FDR |
| GOTERM_BP_FAT | GO:0008104~protein localization | 7 | 3.763441 | 0.931795 | 0.711027 | 1 | 0.999996 | 100 |
| GOTERM_BP_FAT | GO:0015031~protein transport | 5 | 2.688172 | 0.972686 | 0.587857 | 1 | 1 | 100 |
| GOTERM_BP_FAT | GO:0045184~establishment of protein localization | 5 | 2.688172 | 0.974202 | 0.582506 | 1 | 1 | 100 |
