# Supplementary material for: Long non-coding RNA expression profiles of hepatitis C virus-related dysplasia and hepatocellular carcinoma
Source: Oncotarget. 2015 Oct 26;6(41):43770–8. doi: 10.18632/oncotarget.6087 (PMC4791265; doi:10.18632/oncotarget.6087)
Supplement: Supplementary file 4 [file oncotarget-06-43770-s004.docx]

| Annotation Cluster 1 | Enrichment Score: 11.40925641231032 | | | | | | | |  |  |  |  |
| --- | --- | --- | --- | --- | --- | --- | --- | --- | --- | --- | --- | --- |
| Category | Term | | | | | Count | % | PValue | Fold Enrichment | Bonferroni | Benjamini | FDR |
| GOTERM_BP_FAT | GO:0048545~response to steroid hormone stimulus | | | | | 28 | 7.106599 | 5.95E-14 | 6.203878 | 1.30E-10 | 4.34E-11 | 1.03E-10 |
| GOTERM_BP_FAT | GO:0051384~response to glucocorticoid stimulus | | | | | 18 | 4.568528 | 2.18E-12 | 9.817126 | 4.76E-09 | 7.93E-10 | 3.78E-09 |
| GOTERM_BP_FAT | GO:0010033~response to organic substance | | | | | 51 | 12.94416 | 3.76E-12 | 3.009133 | 8.21E-09 | 1.17E-09 | 6.53E-09 |
| GOTERM_BP_FAT | GO:0031960~response to corticosteroid stimulus | | | | | 18 | 4.568528 | 9.55E-12 | 9.008657 | 2.09E-08 | 2.61E-09 | 1.66E-08 |
| GOTERM_BP_FAT | GO:0009719~response to endogenous stimulus | | | | | 36 | 9.137056 | 2.37E-11 | 3.781412 | 5.18E-08 | 5.18E-09 | 4.11E-08 |
| GOTERM_BP_FAT | GO:0009725~response to hormone stimulus | | | | | 34 | 8.629442 | 3.18E-11 | 3.941117 | 6.95E-08 | 5.80E-09 | 5.53E-08 |
|  |  | | | | |  |  |  |  |  |  |  |
| Annotation Cluster 2 | Enrichment Score: 7.141783703128863 | | | | | | | |  |  |  |  |
| Category | Term | | | | | Count | % | PValue | Fold Enrichment | Bonferroni | Benjamini | FDR |
| GOTERM_BP_FAT | GO:0046395~carboxylic acid catabolic process | | | | | 20 | 5.076142 | 1.03E-11 | 7.665024 | 2.25E-08 | 2.50E-09 | 1.79E-08 |
| GOTERM_BP_FAT | GO:0016054~organic acid catabolic process | | | | | 20 | 5.076142 | 1.03E-11 | 7.665024 | 2.25E-08 | 2.50E-09 | 1.79E-08 |
| GOTERM_BP_FAT | GO:0009063~cellular amino acid catabolic process | | | | | 13 | 3.299492 | 5.01E-08 | 8.132815 | 1.09E-04 | 5.76E-06 | 8.70E-05 |
| GOTERM_BP_FAT | GO:0009310~amine catabolic process | | | | | 13 | 3.299492 | 2.42E-07 | 7.090147 | 5.29E-04 | 2.04E-05 | 4.21E-04 |
| GOTERM_BP_FAT | GO:0009074~aromatic amino acid family catabolic process | | | | | 5 | 1.269036 | 1.26E-04 | 17.72537 | 0.241117 | 0.00437 | 0.219018 |
| GOTERM_BP_FAT | GO:0009072~aromatic amino acid family metabolic process | | | | | 5 | 1.269036 | 8.68E-04 | 11.19497 | 0.850172 | 0.022085 | 1.497193 |
|  |  | | | | |  |  |  |  |  |  |  |
| Annotation Cluster 3 | Enrichment Score: 6.447285679412002 | | | | | | | |  |  |  |  |
| Category | Term | | | | | Count | % | PValue | Fold Enrichment | Bonferroni | Benjamini | FDR |
| GOTERM_BP_FAT | GO:0009611~response to wounding | | | | | 51 | 12.94416 | 1.60E-17 | 4.093556 | 3.50E-14 | 1.75E-14 | 2.78E-14 |
| GOTERM_BP_FAT | GO:0006954~inflammatory response | | | | | 35 | 8.883249 | 2.13E-13 | 4.581326 | 4.66E-10 | 1.16E-10 | 3.70E-10 |
| GOTERM_BP_FAT | GO:0002526~acute inflammatory response | | | | | 20 | 5.076142 | 1.01E-12 | 8.681812 | 2.21E-09 | 4.41E-10 | 1.75E-09 |
| GOTERM_BP_FAT | GO:0006952~defense response | | | | | 45 | 11.42132 | 3.13E-11 | 3.112747 | 6.84E-08 | 6.22E-09 | 5.44E-08 |
| GOTERM_BP_FAT | GO:0006955~immune response | | | | | 47 | 11.92893 | 1.16E-10 | 2.897712 | 2.53E-07 | 1.95E-08 | 2.01E-07 |
| GOTERM_BP_FAT | GO:0002541~activation of plasma proteins involved in acute inflammatory response | | | | | 13 | 3.299492 | 1.78E-10 | 12.8612 | 3.90E-07 | 2.78E-08 | 3.10E-07 |
| GOTERM_BP_FAT | GO:0051605~protein maturation by peptide bond cleavage | | | | | 16 | 4.060914 | 1.23E-09 | 7.914582 | 2.69E-06 | 1.79E-07 | 2.14E-06 |
| GOTERM_BP_FAT | GO:0006956~complement activation | | | | | 12 | 3.045685 | 2.20E-09 | 12.15454 | 4.82E-06 | 3.01E-07 | 3.83E-06 |
| GOTERM_BP_FAT | GO:0045087~innate immune response | | | | | 19 | 4.822335 | 3.56E-09 | 5.857078 | 7.79E-06 | 4.58E-07 | 6.19E-06 |
| GOTERM_BP_FAT | GO:0002253~activation of immune response | | | | | 15 | 3.807107 | 3.64E-08 | 6.788438 | 7.96E-05 | 4.42E-06 | 6.33E-05 |
| GOTERM_BP_FAT | GO:0016485~protein processing | | | | | 16 | 4.060914 | 5.12E-08 | 6.077269 | 1.12E-04 | 5.60E-06 | 8.90E-05 |
| GOTERM_BP_FAT | GO:0051604~protein maturation | | | | | 16 | 4.060914 | 1.63E-07 | 5.579132 | 3.55E-04 | 1.55E-05 | 2.83E-04 |
| GOTERM_BP_FAT | GO:0048584~positive regulation of response to stimulus | | | | | 22 | 5.583756 | 1.68E-07 | 3.965675 | 3.67E-04 | 1.53E-05 | 2.92E-04 |
| GOTERM_BP_FAT | GO:0006959~humoral immune response | | | | | 13 | 3.299492 | 2.80E-07 | 7.000398 | 6.11E-04 | 2.26E-05 | 4.86E-04 |
| GOTERM_BP_FAT | GO:0050778~positive regulation of immune response | | | | | 16 | 4.060914 | 1.55E-06 | 4.694166 | 0.003377 | 1.09E-04 | 0.002688 |
| GOTERM_BP_FAT | GO:0002684~positive regulation of immune system process | | | | | 20 | 5.076142 | 3.36E-06 | 3.574864 | 0.007325 | 2.30E-04 | 0.005842 |
| GOTERM_BP_FAT | GO:0006958~complement activation, classical pathway | | | | | 7 | 1.77665 | 4.76E-05 | 10.26849 | 0.098781 | 0.002309 | 0.082618 |
| GOTERM_BP_FAT | GO:0002455~humoral immune response mediated by circulating immunoglobulin | | | | | 7 | 1.77665 | 7.09E-05 | 9.606005 | 0.143563 | 0.002814 | 0.123079 |
| GOTERM_BP_FAT | GO:0002252~immune effector process | | | | | 13 | 3.299492 | 7.29E-05 | 4.1271 | 0.147363 | 0.002843 | 0.126608 |
| GOTERM_BP_FAT | GO:0019724~B cell mediated immunity | | | | | 8 | 2.030457 | 3.14E-04 | 6.077269 | 0.497116 | 0.009246 | 0.54477 |
| GOTERM_BP_FAT | GO:0006957~complement activation, alternative pathway | | | | | 5 | 1.269036 | 3.29E-04 | 14.18029 | 0.5131 | 0.00955 | 0.570296 |
| GOTERM_BP_FAT | GO:0002250~adaptive immune response | | | | | 9 | 2.284264 | 4.30E-04 | 4.972311 | 0.609403 | 0.011829 | 0.744278 |
| GOTERM_BP_FAT | GO:0002460~adaptive immune response based on somatic recombination of immune receptors built from immunoglobulin superfamily domains | | | | | 9 | 2.284264 | 4.30E-04 | 4.972311 | 0.609403 | 0.011829 | 0.744278 |
| GOTERM_BP_FAT | GO:0002449~lymphocyte mediated immunity | | | | | 8 | 2.030457 | 0.001229 | 4.861815 | 0.932071 | 0.029439 | 2.114456 |
| GOTERM_BP_FAT | GO:0016064~immunoglobulin mediated immune response | | | | | 7 | 1.77665 | 0.001583 | 5.514559 | 0.968684 | 0.036177 | 2.714942 |
| GOTERM_BP_FAT | GO:0002443~leukocyte mediated immunity | | | | | 8 | 2.030457 | 0.004013 | 3.957291 | 0.999848 | 0.076802 | 6.747021 |
| GOTERM_BP_FAT | GO:0019835~cytolysis | | | | | 4 | 1.015228 | 0.012398 | 8.103025 | 1 | 0.180497 | 19.48411 |
| GOTERM_BP_FAT | GO:0006508~proteolysis | | | | | 27 | 6.852792 | 0.411552 | 1.089757 | 1 | 0.95342 | 99.99001 |
|  |  | | | | |  |  |  |  |  |  |  |
| Annotation Cluster 4 | Enrichment Score: 5.928496459781746 | | | | | | | |  |  |  |  |
| Category | Term | | | | | Count | % | PValue | Fold Enrichment | Bonferroni | Benjamini | FDR |
| GOTERM_BP_FAT | GO:0042060~wound healing | | | | | 20 | 5.076142 | 1.15E-07 | 4.454542 | 2.52E-04 | 1.15E-05 | 2.01E-04 |
| GOTERM_BP_FAT | GO:0007596~blood coagulation | | | | | 14 | 3.553299 | 7.22E-07 | 5.838944 | 0.001578 | 5.45E-05 | 0.001255 |
| GOTERM_BP_FAT | GO:0050817~coagulation | | | | | 14 | 3.553299 | 7.22E-07 | 5.838944 | 0.001578 | 5.45E-05 | 0.001255 |
| GOTERM_BP_FAT | GO:0007599~hemostasis | | | | | 14 | 3.553299 | 1.40E-06 | 5.514559 | 0.003061 | 1.02E-04 | 0.002436 |
| GOTERM_BP_FAT | GO:0050878~regulation of body fluid levels | | | | | 14 | 3.553299 | 2.69E-05 | 4.223917 | 0.057212 | 0.001472 | 0.046806 |
|  |  | | | | |  |  |  |  |  |  |  |
| Annotation Cluster 5 | Enrichment Score: 3.945229460455979 | | | | | | | |  |  |  |  |
| Category | Term | | | | | Count | % | PValue | Fold Enrichment | Bonferroni | Benjamini | FDR |
| GOTERM_BP_FAT | GO:0006006~glucose metabolic process | | | | | 15 | 3.807107 | 1.44E-05 | 4.170675 | 0.031091 | 9.29E-04 | 0.025097 |
| GOTERM_BP_FAT | GO:0006094~gluconeogenesis | | | | | 7 | 1.77665 | 1.92E-05 | 11.91145 | 0.041097 | 0.001104 | 0.033343 |
| GOTERM_BP_FAT | GO:0016051~carbohydrate biosynthetic process | | | | | 12 | 3.045685 | 4.12E-05 | 4.77094 | 0.086058 | 0.002091 | 0.071486 |
| GOTERM_BP_FAT | GO:0019318~hexose metabolic process | | | | | 16 | 4.060914 | 4.66E-05 | 3.545073 | 0.096941 | 0.002315 | 0.080999 |
| GOTERM_BP_FAT | GO:0006090~pyruvate metabolic process | | | | | 8 | 2.030457 | 4.84E-05 | 8.103025 | 0.100414 | 0.002298 | 0.084058 |
| GOTERM_BP_FAT | GO:0046165~alcohol biosynthetic process | | | | | 8 | 2.030457 | 6.61E-05 | 7.734706 | 0.134458 | 0.002773 | 0.114685 |
| GOTERM_BP_FAT | GO:0005996~monosaccharide metabolic process | | | | | 17 | 4.314721 | 6.96E-05 | 3.257635 | 0.141154 | 0.002814 | 0.12085 |
| GOTERM_BP_FAT | GO:0019319~hexose biosynthetic process | | | | | 7 | 1.77665 | 7.09E-05 | 9.606005 | 0.143563 | 0.002814 | 0.123079 |
| GOTERM_BP_FAT | GO:0046364~monosaccharide biosynthetic process | | | | | 7 | 1.77665 | 1.99E-04 | 8.048275 | 0.352604 | 0.006374 | 0.344929 |
| GOTERM_BP_FAT | GO:0034637~cellular carbohydrate biosynthetic process | | | | | 9 | 2.284264 | 2.01E-04 | 5.548811 | 0.355389 | 0.006344 | 0.348342 |
| GOTERM_BP_FAT | GO:0006091~generation of precursor metabolites and energy | | | | | 12 | 3.045685 | 0.119281 | 1.63096 | 1 | 0.664838 | 88.99138 |
|  |  | | | | |  |  |  |  |  |  |  |
| Annotation Cluster 6 | Enrichment Score: 3.9042435499682733 | | | | | | | |  |  |  |  |
| Category | Term | | | | | Count | % | PValue | Fold Enrichment | Bonferroni | Benjamini | FDR |
| GOTERM_BP_FAT | GO:0046394~carboxylic acid biosynthetic process | | | | | 15 | 3.807107 | 1.68E-05 | 4.116859 | 0.035959 | 0.001017 | 0.029098 |
| GOTERM_BP_FAT | GO:0016053~organic acid biosynthetic process | | | | | 15 | 3.807107 | 1.68E-05 | 4.116859 | 0.035959 | 0.001017 | 0.029098 |
| GOTERM_BP_FAT | GO:0009309~amine biosynthetic process | | | | | 11 | 2.791878 | 1.87E-05 | 5.777157 | 0.039952 | 0.001101 | 0.032396 |
| GOTERM_BP_FAT | GO:0008652~cellular amino acid biosynthetic process | | | | | 8 | 2.030457 | 1.73E-04 | 6.673079 | 0.315594 | 0.005817 | 0.300892 |
| GOTERM_BP_FAT | GO:0009064~glutamine family amino acid metabolic process | | | | | 8 | 2.030457 | 1.96E-04 | 6.544751 | 0.349176 | 0.00639 | 0.340746 |
| GOTERM_BP_FAT | GO:0044271~nitrogen compound biosynthetic process | | | | | 15 | 3.807107 | 0.021047 | 1.963425 | 1 | 0.252207 | 30.89422 |
|  |  | | | | |  |  |  |  |  |  |  |
| Annotation Cluster 7 | Enrichment Score: 3.5130537129102652 | | | | | | | |  |  |  |  |
| Category | Term | | | | | Count | % | PValue | Fold Enrichment | Bonferroni | Benjamini | FDR |
| GOTERM_BP_FAT | GO:0009991~response to extracellular stimulus | | | | | 19 | 4.822335 | 4.33E-06 | 3.673985 | 0.009427 | 2.87E-04 | 0.007527 |
| GOTERM_BP_FAT | GO:0031667~response to nutrient levels | | | | | 16 | 4.060914 | 6.33E-05 | 3.455097 | 0.129194 | 0.002763 | 0.109873 |
| GOTERM_BP_FAT | GO:0007584~response to nutrient | | | | | 11 | 2.791878 | 0.001693 | 3.342498 | 0.975374 | 0.038239 | 2.900574 |
| GOTERM_BP_FAT | GO:0033273~response to vitamin | | | | | 6 | 1.522843 | 0.019101 | 3.867353 | 1 | 0.236815 | 28.46868 |
|  |  | | | | |  |  |  |  |  |  |  |
| Annotation Cluster 8 | Enrichment Score: 3.149392361926455 | | | | | | | |  |  |  |  |
| Category | Term | | | | | Count | % | PValue | Fold Enrichment | Bonferroni | Benjamini | FDR |
| GOTERM_BP_FAT | GO:0042445~hormone metabolic process | | | | | 12 | 3.045685 | 3.77E-05 | 4.815949 | 0.079124 | 0.002008 | 0.065484 |
| GOTERM_BP_FAT | GO:0010817~regulation of hormone levels | | | | | 14 | 3.553299 | 5.54E-05 | 3.944188 | 0.114035 | 0.002468 | 0.096172 |
| GOTERM_BP_FAT | GO:0034754~cellular hormone metabolic process | | | | | 9 | 2.284264 | 6.52E-05 | 6.489287 | 0.132909 | 0.002792 | 0.113266 |
| GOTERM_BP_FAT | GO:0019748~secondary metabolic process | | | | | 10 | 2.538071 | 9.27E-05 | 5.384922 | 0.183413 | 0.003428 | 0.16089 |
| GOTERM_BP_FAT | GO:0006775~fat-soluble vitamin metabolic process | | | | | 6 | 1.522843 | 9.48E-04 | 7.734706 | 0.874129 | 0.023811 | 1.633486 |
| GOTERM_BP_FAT | GO:0006776~vitamin A metabolic process | | | | | 5 | 1.269036 | 0.001842 | 9.248018 | 0.982227 | 0.040696 | 3.151886 |
| GOTERM_BP_FAT | GO:0016101~diterpenoid metabolic process | | | | | 5 | 1.269036 | 0.001842 | 9.248018 | 0.982227 | 0.040696 | 3.151886 |
| GOTERM_BP_FAT | GO:0001523~retinoid metabolic process | | | | | 5 | 1.269036 | 0.001842 | 9.248018 | 0.982227 | 0.040696 | 3.151886 |
| GOTERM_BP_FAT | GO:0006766~vitamin metabolic process | | | | | 8 | 2.030457 | 0.001843 | 4.537694 | 0.982279 | 0.040318 | 3.154121 |
| GOTERM_BP_FAT | GO:0042573~retinoic acid metabolic process | | | | | 4 | 1.015228 | 0.002396 | 14.18029 | 0.994723 | 0.049643 | 4.082033 |
| GOTERM_BP_FAT | GO:0006721~terpenoid metabolic process | | | | | 5 | 1.269036 | 0.002536 | 8.508176 | 0.996117 | 0.051976 | 4.315433 |
| GOTERM_BP_FAT | GO:0006720~isoprenoid metabolic process | | | | | 5 | 1.269036 | 0.019242 | 4.834191 | 1 | 0.23702 | 28.64608 |
|  |  | | | | |  |  |  |  |  |  |  |
| Annotation Cluster 9 | Enrichment Score: 3.1264063699554776 | | | | | | | |  |  |  |  |
| Category | Term | | | | | Count | % | PValue | Fold Enrichment | Bonferroni | Benjamini | FDR |
| GOTERM_BP_FAT | GO:0019439~aromatic compound catabolic process | | | | | 8 | 2.030457 | 2.17E-07 | 17.01635 | 4.73E-04 | 1.89E-05 | 3.76E-04 |
| GOTERM_BP_FAT | GO:0009074~aromatic amino acid family catabolic process | | | | | 5 | 1.269036 | 1.26E-04 | 17.72537 | 0.241117 | 0.00437 | 0.219018 |
| GOTERM_BP_FAT | GO:0009072~aromatic amino acid family metabolic process | | | | | 5 | 1.269036 | 8.68E-04 | 11.19497 | 0.850172 | 0.022085 | 1.497193 |
| GOTERM_BP_FAT | GO:0006572~tyrosine catabolic process | | | | | 3 | 0.761421 | 0.005224 | 25.52453 | 0.999989 | 0.094758 | 8.696351 |
| GOTERM_BP_FAT | GO:0006559~L-phenylalanine catabolic process | | | | | 3 | 0.761421 | 0.007714 | 21.27044 | 1 | 0.127614 | 12.58742 |
| GOTERM_BP_FAT | GO:0006558~L-phenylalanine metabolic process | | | | | 3 | 0.761421 | 0.007714 | 21.27044 | 1 | 0.127614 | 12.58742 |
| GOTERM_BP_FAT | GO:0006570~tyrosine metabolic process | | | | | 3 | 0.761421 | 0.017673 | 14.18029 | 1 | 0.223621 | 26.63798 |
|  |  | | | | |  |  |  |  |  |  |  |
| Annotation Cluster 10 | Enrichment Score: 3.0132464639729797 | | | | | | | |  |  |  |  |
| Category | Term | | | | | Count | % | PValue | Fold Enrichment | Bonferroni | Benjamini | FDR |
| GOTERM_BP_FAT | GO:0042330~taxis | | | | | 14 | 3.553299 | 1.00E-04 | 3.722327 | 0.197126 | 0.003593 | 0.174326 |
| GOTERM_BP_FAT | GO:0006935~chemotaxis | | | | | 14 | 3.553299 | 1.00E-04 | 3.722327 | 0.197126 | 0.003593 | 0.174326 |
| GOTERM_BP_FAT | GO:0007610~behavior | | | | | 26 | 6.598985 | 1.15E-04 | 2.358343 | 0.222427 | 0.004049 | 0.199725 |
| GOTERM_BP_FAT | GO:0007626~locomotory behavior | | | | | 17 | 4.314721 | 7.52E-04 | 2.639398 | 0.806985 | 0.019861 | 1.298731 |
| GOTERM_BP_FAT | GO:0007186~G-protein coupled receptor protein signaling pathway | | | | | 18 | 4.568528 | 0.983258 | 0.681866 | 1 | 1 | 100 |
|  |  | | | | |  |  |  |  |  |  |  |
| Annotation Cluster 11 | Enrichment Score: 2.8787805375201323 | | | | | | | |  |  |  |  |
| Category | Term | | | | | Count | % | PValue | Fold Enrichment | Bonferroni | Benjamini | FDR |
| GOTERM_BP_FAT | GO:0008202~steroid metabolic process | | | | | 15 | 3.807107 | 2.95E-04 | 3.158976 | 0.47541 | 0.008799 | 0.511366 |
| GOTERM_BP_FAT | GO:0006694~steroid biosynthetic process | | | | | 9 | 2.284264 | 8.37E-04 | 4.504329 | 0.839565 | 0.021549 | 1.443638 |
| GOTERM_BP_FAT | GO:0008610~lipid biosynthetic process | | | | | 16 | 4.060914 | 0.009357 | 2.107288 | 1 | 0.144172 | 15.06728 |
|  |  | | | | |  |  |  |  |  |  |  |
| Annotation Cluster 12 | Enrichment Score: 2.79329566478513 | | | | | | | |  |  |  |  |
| Category | Term | | | | | Count | % | PValue | Fold Enrichment | Bonferroni | Benjamini | FDR |
| GOTERM_BP_FAT | GO:0017144~drug metabolic process | | | | | 6 | 1.522843 | 2.42E-05 | 15.95283 | 0.051486 | 0.001354 | 0.041997 |
| GOTERM_BP_FAT | GO:0006805~xenobiotic metabolic process | | | | | 4 | 1.015228 | 0.010811 | 8.508176 | 1 | 0.161393 | 17.20732 |
| GOTERM_BP_FAT | GO:0009410~response to xenobiotic stimulus | | | | | 4 | 1.015228 | 0.015951 | 7.398414 | 1 | 0.210143 | 24.37073 |
|  |  | | | | |  |  |  |  |  |  |  |
| Annotation Cluster 13 | Enrichment Score: 2.725354636214866 | | | | | | | |  |  |  |  |
| Category | Term | | | | | Count | % | PValue | Fold Enrichment | Bonferroni | Benjamini | FDR |
| GOTERM_BP_FAT | GO:0008652~cellular amino acid biosynthetic process | | | | | 8 | 2.030457 | 1.73E-04 | 6.673079 | 0.315594 | 0.005817 | 0.300892 |
| GOTERM_BP_FAT | GO:0009064~glutamine family amino acid metabolic process | | | | | 8 | 2.030457 | 1.96E-04 | 6.544751 | 0.349176 | 0.00639 | 0.340746 |
| GOTERM_BP_FAT | GO:0043603~cellular amide metabolic process | | | | | 8 | 2.030457 | 3.14E-04 | 6.077269 | 0.497116 | 0.009246 | 0.54477 |
| GOTERM_BP_FAT | GO:0019627~urea metabolic process | | | | | 4 | 1.015228 | 0.001353 | 17.01635 | 0.948205 | 0.032009 | 2.325166 |
| GOTERM_BP_FAT | GO:0000050~urea cycle | | | | | 4 | 1.015228 | 0.001353 | 17.01635 | 0.948205 | 0.032009 | 2.325166 |
| GOTERM_BP_FAT | GO:0043604~amide biosynthetic process | | | | | 4 | 1.015228 | 0.001829 | 15.46941 | 0.981708 | 0.040823 | 3.129716 |
| GOTERM_BP_FAT | GO:0006526~arginine biosynthetic process | | | | | 3 | 0.761421 | 0.003183 | 31.90566 | 0.99906 | 0.061943 | 5.388244 |
| GOTERM_BP_FAT | GO:0006525~arginine metabolic process | | | | | 3 | 0.761421 | 0.036007 | 9.817126 | 1 | 0.360991 | 47.11429 |
| GOTERM_BP_FAT | GO:0009084~glutamine family amino acid biosynthetic process | | | | | 3 | 0.761421 | 0.072044 | 6.716981 | 1 | 0.53244 | 72.71675 |
|  |  | | | | |  |  |  |  |  |  |  |
| Annotation Cluster 14 | Enrichment Score: 2.715000927319888 | | | | | | | |  |  |  |  |
| Category | Term | | | | | Count | % | PValue | Fold Enrichment | Bonferroni | Benjamini | FDR |
| GOTERM_BP_FAT | GO:0009617~response to bacterium | | | | | 16 | 4.060914 | 4.97E-05 | 3.526705 | 0.102951 | 0.002309 | 0.0863 |
| GOTERM_BP_FAT | GO:0032496~response to lipopolysaccharide | | | | | 7 | 1.77665 | 0.009297 | 3.867353 | 1 | 0.145347 | 14.9776 |
| GOTERM_BP_FAT | GO:0002237~response to molecule of bacterial origin | | | | | 7 | 1.77665 | 0.0155 | 3.46263 | 1 | 0.208548 | 23.76603 |
|  |  | | | | |  |  |  |  |  |  |  |
| Annotation Cluster 15 | Enrichment Score: 2.5951081071411 | | | | | | | |  |  |  |  |
| Category | Term | | | | | Count | % | PValue | Fold Enrichment | Bonferroni | Benjamini | FDR |
| GOTERM_BP_FAT | GO:0008202~steroid metabolic process | | | | | 15 | 3.807107 | 2.95E-04 | 3.158976 | 0.47541 | 0.008799 | 0.511366 |
| GOTERM_BP_FAT | GO:0008203~cholesterol metabolic process | | | | | 8 | 2.030457 | 0.005812 | 3.699207 | 0.999997 | 0.101546 | 9.630363 |
| GOTERM_BP_FAT | GO:0016125~sterol metabolic process | | | | | 8 | 2.030457 | 0.009559 | 3.369575 | 1 | 0.146034 | 15.36734 |
|  |  | | | | |  |  |  |  |  |  |  |
| Annotation Cluster 16 | Enrichment Score: 2.5739730828150216 | | | | | | | |  |  |  |  |
| Category | Term | | | | | Count | % | PValue | Fold Enrichment | Bonferroni | Benjamini | FDR |
| GOTERM_BP_FAT | GO:0006694~steroid biosynthetic process | | | | | 9 | 2.284264 | 8.37E-04 | 4.504329 | 0.839565 | 0.021549 | 1.443638 |
| GOTERM_BP_FAT | GO:0008209~androgen metabolic process | | | | | 4 | 1.015228 | 0.001829 | 15.46941 | 0.981708 | 0.040823 | 3.129716 |
| GOTERM_BP_FAT | GO:0006706~steroid catabolic process | | | | | 4 | 1.015228 | 0.012398 | 8.103025 | 1 | 0.180497 | 19.48411 |
|  |  | | | | |  |  |  |  |  |  |  |
| Annotation Cluster 17 | Enrichment Score: 2.4225965304194643 | | | | | | | |  |  |  |  |
| Category | Term | | | | | Count | % | PValue | Fold Enrichment | Bonferroni | Benjamini | FDR |
| GOTERM_BP_FAT | GO:0008209~androgen metabolic process | | | | | 4 | 1.015228 | 0.001829 | 15.46941 | 0.981708 | 0.040823 | 3.129716 |
| GOTERM_BP_FAT | GO:0006702~androgen biosynthetic process | | | | | 3 | 0.761421 | 0.005224 | 25.52453 | 0.999989 | 0.094758 | 8.696351 |
| GOTERM_BP_FAT | GO:0042446~hormone biosynthetic process | | | | | 5 | 1.269036 | 0.005651 | 6.861432 | 0.999996 | 0.099655 | 9.374913 |
|  |  | | | | |  |  |  |  |  |  |  |
| Annotation Cluster 18 | Enrichment Score: 2.312056896205311 | | | | | | | |  |  |  |  |
| Category | Term | | | | | Count | % | PValue | Fold Enrichment | Bonferroni | Benjamini | FDR |
| GOTERM_BP_FAT | GO:0009066~aspartate family amino acid metabolic process | | | | | 6 | 1.522843 | 2.02E-04 | 10.63522 | 0.356554 | 0.006279 | 0.349775 |
| GOTERM_BP_FAT | GO:0000096~sulfur amino acid metabolic process | | | | | 4 | 1.015228 | 0.015951 | 7.398414 | 1 | 0.210143 | 24.37073 |
| GOTERM_BP_FAT | GO:0006555~methionine metabolic process | | | | | 3 | 0.761421 | 0.036007 | 9.817126 | 1 | 0.360991 | 47.11429 |
|  |  | | | | |  |  |  |  |  |  |  |
| Annotation Cluster 19 | Enrichment Score: 2.1783466000293608 | | | | | | | |  |  |  |  |
| Category | Term | | | | | Count | % | PValue | Fold Enrichment | Bonferroni | Benjamini | FDR |
| GOTERM_BP_FAT | GO:0030193~regulation of blood coagulation | | | | | 7 | 1.77665 | 1.70E-04 | 8.271838 | 0.310319 | 0.005788 | 0.294809 |
| GOTERM_BP_FAT | GO:0050818~regulation of coagulation | | | | | 7 | 1.77665 | 3.56E-04 | 7.263077 | 0.540403 | 0.010045 | 0.615885 |
| GOTERM_BP_FAT | GO:0051917~regulation of fibrinolysis | | | | | 4 | 1.015228 | 9.64E-04 | 18.90706 | 0.878566 | 0.023943 | 1.661533 |
| GOTERM_BP_FAT | GO:0030194~positive regulation of blood coagulation | | | | | 3 | 0.761421 | 0.021753 | 12.76226 | 1 | 0.258159 | 31.75502 |
| GOTERM_BP_FAT | GO:0050820~positive regulation of coagulation | | | | | 3 | 0.761421 | 0.036007 | 9.817126 | 1 | 0.360991 | 47.11429 |
| GOTERM_BP_FAT | GO:0030195~negative regulation of blood coagulation | | | | | 3 | 0.761421 | 0.100355 | 5.548811 | 1 | 0.616821 | 84.07283 |
| GOTERM_BP_FAT | GO:0050819~negative regulation of coagulation | | | | | 3 | 0.761421 | 0.123257 | 4.908563 | 1 | 0.67335 | 89.82354 |
|  |  | | | | |  |  |  |  |  |  |  |
| Annotation Cluster 20 | Enrichment Score: 2.091250523837991 | | | | | | | |  |  |  |  |
| Category | Term | | | | | Count | % | PValue | Fold Enrichment | Bonferroni | Benjamini | FDR |
| GOTERM_BP_FAT | GO:0051260~protein homooligomerization | | | | | 10 | 2.538071 | 3.82E-04 | 4.477987 | 0.565826 | 0.010639 | 0.660816 |
| GOTERM_BP_FAT | GO:0051289~protein homotetramerization | | | | | 4 | 1.015228 | 0.022244 | 6.544751 | 1 | 0.261807 | 32.34748 |
| GOTERM_BP_FAT | GO:0051262~protein tetramerization | | | | | 4 | 1.015228 | 0.062724 | 4.363167 | 1 | 0.493782 | 67.54441 |
|  |  | | | | |  |  |  |  |  |  |  |
| Annotation Cluster 21 | Enrichment Score: 1.964383396202116 | | | | | | | |  |  |  |  |
| Category | Term | | | | | Count | % | PValue | Fold Enrichment | Bonferroni | Benjamini | FDR |
| GOTERM_BP_FAT | GO:0048878~chemical homeostasis | | | | | 28 | 7.106599 | 7.41E-05 | 2.326454 | 0.149552 | 0.002838 | 0.128649 |
| GOTERM_BP_FAT | GO:0042592~homeostatic process | | | | | 32 | 8.121827 | 0.001572 | 1.812661 | 0.967894 | 0.0363 | 2.695688 |
| GOTERM_BP_FAT | GO:0055066~di-, tri-valent inorganic cation homeostasis | | | | | 14 | 3.553299 | 0.00427 | 2.491934 | 0.999913 | 0.079445 | 7.163836 |
| GOTERM_BP_FAT | GO:0030003~cellular cation homeostasis | | | | | 14 | 3.553299 | 0.006991 | 2.344773 | 1 | 0.119044 | 11.47415 |
| GOTERM_BP_FAT | GO:0030005~cellular di-, tri-valent inorganic cation homeostasis | | | | | 13 | 3.299492 | 0.007339 | 2.436262 | 1 | 0.123643 | 12.0109 |
| GOTERM_BP_FAT | GO:0055080~cation homeostasis | | | | | 15 | 3.807107 | 0.007572 | 2.231165 | 1 | 0.126354 | 12.36895 |
| GOTERM_BP_FAT | GO:0050801~ion homeostasis | | | | | 18 | 4.568528 | 0.016448 | 1.872215 | 1 | 0.214706 | 25.03192 |
| GOTERM_BP_FAT | GO:0006875~cellular metal ion homeostasis | | | | | 11 | 2.791878 | 0.017273 | 2.387498 | 1 | 0.220375 | 26.11633 |
| GOTERM_BP_FAT | GO:0055065~metal ion homeostasis | | | | | 11 | 2.791878 | 0.022745 | 2.282681 | 1 | 0.265493 | 32.94659 |
| GOTERM_BP_FAT | GO:0006874~cellular calcium ion homeostasis | | | | | 10 | 2.538071 | 0.0286 | 2.324638 | 1 | 0.312942 | 39.59397 |
| GOTERM_BP_FAT | GO:0055074~calcium ion homeostasis | | | | | 10 | 2.538071 | 0.033181 | 2.262813 | 1 | 0.342375 | 44.35607 |
| GOTERM_BP_FAT | GO:0055082~cellular chemical homeostasis | | | | | 16 | 4.060914 | 0.034463 | 1.791195 | 1 | 0.351524 | 45.62325 |
| GOTERM_BP_FAT | GO:0006873~cellular ion homeostasis | | | | | 15 | 3.807107 | 0.05777 | 1.706185 | 1 | 0.478164 | 64.43195 |
| GOTERM_BP_FAT | GO:0019725~cellular homeostasis | | | | | 16 | 4.060914 | 0.133639 | 1.460631 | 1 | 0.697871 | 91.72578 |
|  |  | | | | |  |  |  |  |  |  |  |
| Annotation Cluster 22 | Enrichment Score: 1.9191088764728528 | | | | | | | |  |  |  |  |
| Category | Term | | | | | Count | % | PValue | Fold Enrichment | Bonferroni | Benjamini | FDR |
| GOTERM_BP_FAT | GO:0008015~blood circulation | | | | | 13 | 3.299492 | 0.001465 | 2.973287 | 0.959393 | 0.034225 | 2.513862 |
| GOTERM_BP_FAT | GO:0003013~circulatory system process | | | | | 13 | 3.299492 | 0.001465 | 2.973287 | 0.959393 | 0.034225 | 2.513862 |
| GOTERM_BP_FAT | GO:0008217~regulation of blood pressure | | | | | 9 | 2.284264 | 0.002393 | 3.828679 | 0.994684 | 0.050048 | 4.076354 |
| GOTERM_BP_FAT | GO:0050880~regulation of blood vessel size | | | | | 5 | 1.269036 | 0.03529 | 4.013291 | 1 | 0.356755 | 46.4274 |
| GOTERM_BP_FAT | GO:0035150~regulation of tube size | | | | | 5 | 1.269036 | 0.03529 | 4.013291 | 1 | 0.356755 | 46.4274 |
| GOTERM_BP_FAT | GO:0003018~vascular process in circulatory system | | | | | 5 | 1.269036 | 0.046753 | 3.667317 | 1 | 0.425236 | 56.47226 |
| GOTERM_BP_FAT | GO:0042311~vasodilation | | | | | 3 | 0.761421 | 0.123257 | 4.908563 | 1 | 0.67335 | 89.82354 |
|  |  | | | | |  |  |  |  |  |  |  |
| Annotation Cluster 23 | Enrichment Score: 1.8925820293539877 | | | | | | | |  |  |  |  |
| Category | Term | | | | | Count | % | PValue | Fold Enrichment | Bonferroni | Benjamini | FDR |
| GOTERM_BP_FAT | GO:0010953~regulation of protein maturation by peptide bond cleavage | | | | | 3 | 0.761421 | 0.007714 | 21.27044 | 1 | 0.127614 | 12.58742 |
| GOTERM_BP_FAT | GO:0070613~regulation of protein processing | | | | | 3 | 0.761421 | 0.007714 | 21.27044 | 1 | 0.127614 | 12.58742 |
| GOTERM_BP_FAT | GO:0030162~regulation of proteolysis | | | | | 5 | 1.269036 | 0.03529 | 4.013291 | 1 | 0.356755 | 46.4274 |
|  |  | | | | |  |  |  |  |  |  |  |
| Annotation Cluster 24 | Enrichment Score: 1.838532686482216 | | | | | | | |  |  |  |  |
| Category | Term | | | | | Count | % | PValue | Fold Enrichment | Bonferroni | Benjamini | FDR |
| GOTERM_BP_FAT | GO:0046700~heterocycle catabolic process | | | | | 8 | 2.030457 | 0.002146 | 4.419832 | 0.990877 | 0.045884 | 3.6638 |
| GOTERM_BP_FAT | GO:0006548~histidine catabolic process | | | | | 3 | 0.761421 | 0.017673 | 14.18029 | 1 | 0.223621 | 26.63798 |
| GOTERM_BP_FAT | GO:0009077~histidine family amino acid catabolic process | | | | | 3 | 0.761421 | 0.017673 | 14.18029 | 1 | 0.223621 | 26.63798 |
| GOTERM_BP_FAT | GO:0006547~histidine metabolic process | | | | | 3 | 0.761421 | 0.030938 | 10.63522 | 1 | 0.329287 | 42.07025 |
| GOTERM_BP_FAT | GO:0009075~histidine family amino acid metabolic process | | | | | 3 | 0.761421 | 0.030938 | 10.63522 | 1 | 0.329287 | 42.07025 |
|  |  | | | | |  |  |  |  |  |  |  |
| Annotation Cluster 25 | Enrichment Score: 1.7964491059812844 | | | | | | | |  |  |  |  |
| Category | Term | | | | | Count | % | PValue | Fold Enrichment | Bonferroni | Benjamini | FDR |
| GOTERM_BP_FAT | GO:0043434~response to peptide hormone stimulus | | | | | 13 | 3.299492 | 2.73E-04 | 3.591113 | 0.449712 | 0.008262 | 0.473548 |
| GOTERM_BP_FAT | GO:0032870~cellular response to hormone stimulus | | | | | 9 | 2.284264 | 0.013038 | 2.878706 | 1 | 0.186481 | 20.38556 |
| GOTERM_BP_FAT | GO:0032868~response to insulin stimulus | | | | | 6 | 1.522843 | 0.085768 | 2.552453 | 1 | 0.575134 | 78.93867 |
| GOTERM_BP_FAT | GO:0032869~cellular response to insulin stimulus | | | | | 4 | 1.015228 | 0.213398 | 2.502405 | 1 | 0.825045 | 98.45443 |
|  |  | | | | |  |  |  |  |  |  |  |
| Annotation Cluster 26 | Enrichment Score: 1.7291325083293034 | | | | | | | |  |  |  |  |
| Category | Term | | | | | Count | % | PValue | Fold Enrichment | Bonferroni | Benjamini | FDR |
| GOTERM_BP_FAT | GO:0034369~plasma lipoprotein particle remodeling | | | | | 6 | 1.522843 | 7.95E-05 | 12.76226 | 0.159444 | 0.00299 | 0.137934 |
| GOTERM_BP_FAT | GO:0034367~macromolecular complex remodeling | | | | | 6 | 1.522843 | 7.95E-05 | 12.76226 | 0.159444 | 0.00299 | 0.137934 |
| GOTERM_BP_FAT | GO:0034368~protein-lipid complex remodeling | | | | | 6 | 1.522843 | 7.95E-05 | 12.76226 | 0.159444 | 0.00299 | 0.137934 |
| GOTERM_BP_FAT | GO:0055088~lipid homeostasis | | | | | 6 | 1.522843 | 0.006659 | 5.004809 | 1 | 0.114602 | 10.95889 |
| GOTERM_BP_FAT | GO:0042439~ethanolamine and derivative metabolic process | | | | | 5 | 1.269036 | 0.007884 | 6.256012 | 1 | 0.12831 | 12.84656 |
| GOTERM_BP_FAT | GO:0046470~phosphatidylcholine metabolic process | | | | | 4 | 1.015228 | 0.012398 | 8.103025 | 1 | 0.180497 | 19.48411 |
| GOTERM_BP_FAT | GO:0046486~glycerolipid metabolic process | | | | | 10 | 2.538071 | 0.014144 | 2.62598 | 1 | 0.195675 | 21.92119 |
| GOTERM_BP_FAT | GO:0034370~triglyceride-rich lipoprotein particle remodeling | | | | | 3 | 0.761421 | 0.017673 | 14.18029 | 1 | 0.223621 | 26.63798 |
| GOTERM_BP_FAT | GO:0034374~low-density lipoprotein particle remodeling | | | | | 3 | 0.761421 | 0.017673 | 14.18029 | 1 | 0.223621 | 26.63798 |
| GOTERM_BP_FAT | GO:0010743~regulation of foam cell differentiation | | | | | 4 | 1.015228 | 0.020017 | 6.806541 | 1 | 0.242701 | 29.62003 |
| GOTERM_BP_FAT | GO:0034375~high-density lipoprotein particle remodeling | | | | | 3 | 0.761421 | 0.030938 | 10.63522 | 1 | 0.329287 | 42.07025 |
| GOTERM_BP_FAT | GO:0043691~reverse cholesterol transport | | | | | 3 | 0.761421 | 0.052913 | 7.976415 | 1 | 0.458051 | 61.10895 |
| GOTERM_BP_FAT | GO:0006869~lipid transport | | | | | 8 | 2.030457 | 0.054504 | 2.347083 | 1 | 0.463077 | 62.22789 |
| GOTERM_BP_FAT | GO:0015918~sterol transport | | | | | 4 | 1.015228 | 0.055154 | 4.599014 | 1 | 0.463778 | 62.67653 |
| GOTERM_BP_FAT | GO:0030301~cholesterol transport | | | | | 4 | 1.015228 | 0.055154 | 4.599014 | 1 | 0.463778 | 62.67653 |
| GOTERM_BP_FAT | GO:0010876~lipid localization | | | | | 8 | 2.030457 | 0.076398 | 2.167688 | 1 | 0.549289 | 74.85728 |
| GOTERM_BP_FAT | GO:0019637~organophosphate metabolic process | | | | | 8 | 2.030457 | 0.189736 | 1.701635 | 1 | 0.793009 | 97.41364 |
| GOTERM_BP_FAT | GO:0055092~sterol homeostasis | | | | | 3 | 0.761421 | 0.223459 | 3.358491 | 1 | 0.833868 | 98.76413 |
| GOTERM_BP_FAT | GO:0042632~cholesterol homeostasis | | | | | 3 | 0.761421 | 0.223459 | 3.358491 | 1 | 0.833868 | 98.76413 |
| GOTERM_BP_FAT | GO:0006644~phospholipid metabolic process | | | | | 7 | 1.77665 | 0.287227 | 1.567296 | 1 | 0.887339 | 99.72106 |
| GOTERM_BP_FAT | GO:0006650~glycerophospholipid metabolic process | | | | | 5 | 1.269036 | 0.299415 | 1.80258 | 1 | 0.895093 | 99.79328 |
|  |  | | | | |  |  |  |  |  |  |  |
| Annotation Cluster 27 | Enrichment Score: 1.7175382997034212 | | | | | | | |  |  |  |  |
| Category | Term | | | | | Count | % | PValue | Fold Enrichment | Bonferroni | Benjamini | FDR |
| GOTERM_BP_FAT | GO:0018904~organic ether metabolic process | | | | | 6 | 1.522843 | 0.007833 | 4.815949 | 1 | 0.128492 | 12.76943 |
| GOTERM_BP_FAT | GO:0046486~glycerolipid metabolic process | | | | | 10 | 2.538071 | 0.014144 | 2.62598 | 1 | 0.195675 | 21.92119 |
| GOTERM_BP_FAT | GO:0006641~triglyceride metabolic process | | | | | 5 | 1.269036 | 0.017812 | 4.946614 | 1 | 0.223898 | 26.81752 |
| GOTERM_BP_FAT | GO:0006639~acylglycerol metabolic process | | | | | 5 | 1.269036 | 0.027439 | 4.340906 | 1 | 0.303734 | 38.32706 |
| GOTERM_BP_FAT | GO:0006638~neutral lipid metabolic process | | | | | 5 | 1.269036 | 0.029293 | 4.254088 | 1 | 0.317708 | 40.33755 |
| GOTERM_BP_FAT | GO:0006662~glycerol ether metabolic process | | | | | 5 | 1.269036 | 0.031219 | 4.170675 | 1 | 0.330194 | 42.36116 |
|  |  | | | | |  |  |  |  |  |  |  |
| Annotation Cluster 28 | Enrichment Score: 1.6645810478750858 | | | | | | | |  |  |  |  |
| Category | Term | | | | | Count | % | PValue | Fold Enrichment | Bonferroni | Benjamini | FDR |
| GOTERM_BP_FAT | GO:0005976~polysaccharide metabolic process | | | | | 10 | 2.538071 | 0.001185 | 3.832512 | 0.925086 | 0.028697 | 2.038285 |
| GOTERM_BP_FAT | GO:0006022~aminoglycan metabolic process | | | | | 7 | 1.77665 | 0.004087 | 4.581326 | 0.99987 | 0.076814 | 6.866512 |
| GOTERM_BP_FAT | GO:0030203~glycosaminoglycan metabolic process | | | | | 6 | 1.522843 | 0.009144 | 4.640823 | 1 | 0.144154 | 14.74966 |
| GOTERM_BP_FAT | GO:0000272~polysaccharide catabolic process | | | | | 4 | 1.015228 | 0.027087 | 6.077269 | 1 | 0.301942 | 37.93801 |
| GOTERM_BP_FAT | GO:0006026~aminoglycan catabolic process | | | | | 3 | 0.761421 | 0.085842 | 6.077269 | 1 | 0.57388 | 78.96847 |
| GOTERM_BP_FAT | GO:0009057~macromolecule catabolic process | | | | | 7 | 1.77665 | 0.99983 | 0.381288 | 1 | 1 | 100 |
|  |  | | | | |  |  |  |  |  |  |  |
| Annotation Cluster 29 | Enrichment Score: 1.5572711268881034 | | | | | | | |  |  |  |  |
| Category | Term | | | | | Count | % | PValue | Fold Enrichment | Bonferroni | Benjamini | FDR |
| GOTERM_BP_FAT | GO:0006071~glycerol metabolic process | | | | | 4 | 1.015228 | 0.015951 | 7.398414 | 1 | 0.210143 | 24.37073 |
| GOTERM_BP_FAT | GO:0019400~alditol metabolic process | | | | | 4 | 1.015228 | 0.020017 | 6.806541 | 1 | 0.242701 | 29.62003 |
| GOTERM_BP_FAT | GO:0019751~polyol metabolic process | | | | | 4 | 1.015228 | 0.066681 | 4.254088 | 1 | 0.510775 | 69.84387 |
|  |  | | | | |  |  |  |  |  |  |  |
| Annotation Cluster 30 | Enrichment Score: 1.4984808667974427 | | | | | | | |  |  |  |  |
| Category | Term | | | | | Count | % | PValue | Fold Enrichment | Bonferroni | Benjamini | FDR |
| GOTERM_BP_FAT | GO:0051260~protein homooligomerization | | | | | 10 | 2.538071 | 3.82E-04 | 4.477987 | 0.565826 | 0.010639 | 0.660816 |
| GOTERM_BP_FAT | GO:0051259~protein oligomerization | | | | | 12 | 3.045685 | 0.002674 | 2.933854 | 0.997128 | 0.054216 | 4.544581 |
| GOTERM_BP_FAT | GO:0043933~macromolecular complex subunit organization | | | | | 23 | 5.837563 | 0.109831 | 1.378085 | 1 | 0.642874 | 86.74891 |
| GOTERM_BP_FAT | GO:0070271~protein complex biogenesis | | | | | 17 | 4.314721 | 0.135521 | 1.432069 | 1 | 0.701932 | 92.03261 |
| GOTERM_BP_FAT | GO:0006461~protein complex assembly | | | | | 17 | 4.314721 | 0.135521 | 1.432069 | 1 | 0.701932 | 92.03261 |
| GOTERM_BP_FAT | GO:0065003~macromolecular complex assembly | | | | | 17 | 4.314721 | 0.496195 | 1.087511 | 1 | 0.974145 | 99.99933 |
|  |  | | | | |  |  |  |  |  |  |  |
| Annotation Cluster 31 | Enrichment Score: 1.4515608083988962 | | | | | | | |  |  |  |  |
| Category | Term | | | | | Count | % | PValue | Fold Enrichment | Bonferroni | Benjamini | FDR |
| GOTERM_BP_FAT | GO:0031099~regeneration | | | | | 7 | 1.77665 | 0.005488 | 4.315742 | 0.999994 | 0.097706 | 9.116806 |
| GOTERM_BP_FAT | GO:0042246~tissue regeneration | | | | | 4 | 1.015228 | 0.038313 | 5.31761 | 1 | 0.377768 | 49.27006 |
| GOTERM_BP_FAT | GO:0048589~developmental growth | | | | | 6 | 1.522843 | 0.053442 | 2.933854 | 1 | 0.459738 | 61.48435 |
| GOTERM_BP_FAT | GO:0040007~growth | | | | | 8 | 2.030457 | 0.139036 | 1.859711 | 1 | 0.706433 | 92.57696 |
|  |  | | | | |  |  |  |  |  |  |  |
| Annotation Cluster 32 | Enrichment Score: 1.3419617747701253 | | | | | | | |  |  |  |  |
| Category | Term | | | | | Count | % | PValue | Fold Enrichment | Bonferroni | Benjamini | FDR |
| GOTERM_BP_FAT | GO:0009712~catechol metabolic process | | | | | 4 | 1.015228 | 0.044683 | 5.004809 | 1 | 0.415642 | 54.80125 |
| GOTERM_BP_FAT | GO:0006584~catecholamine metabolic process | | | | | 4 | 1.015228 | 0.044683 | 5.004809 | 1 | 0.415642 | 54.80125 |
| GOTERM_BP_FAT | GO:0034311~diol metabolic process | | | | | 4 | 1.015228 | 0.044683 | 5.004809 | 1 | 0.415642 | 54.80125 |
| GOTERM_BP_FAT | GO:0018958~phenol metabolic process | | | | | 4 | 1.015228 | 0.048053 | 4.861815 | 1 | 0.430855 | 57.49224 |
|  |  | | | | |  |  |  |  |  |  |  |
| Annotation Cluster 33 | Enrichment Score: 1.2339174801540485 | | | | | | | |  |  |  |  |
| Category | Term | | | | | Count | % | PValue | Fold Enrichment | Bonferroni | Benjamini | FDR |
| GOTERM_BP_FAT | GO:0051241~negative regulation of multicellular organismal process | | | | | 10 | 2.538071 | 0.015217 | 2.593956 | 1 | 0.206399 | 23.3852 |
| GOTERM_BP_FAT | GO:0051051~negative regulation of transport | | | | | 7 | 1.77665 | 0.097809 | 2.205823 | 1 | 0.609933 | 83.27149 |
| GOTERM_BP_FAT | GO:0001817~regulation of cytokine production | | | | | 8 | 2.030457 | 0.133516 | 1.88026 | 1 | 0.698899 | 91.70551 |
|  |  | | | | |  |  |  |  |  |  |  |
| Annotation Cluster 34 | Enrichment Score: 1.205516076236743 | | | | | | | |  |  |  |  |
| Category | Term | | | | | Count | % | PValue | Fold Enrichment | Bonferroni | Benjamini | FDR |
| GOTERM_BP_FAT | GO:0032103~positive regulation of response to external stimulus | | | | | 6 | 1.522843 | 0.016919 | 3.988208 | 1 | 0.217611 | 25.65298 |
| GOTERM_BP_FAT | GO:0031349~positive regulation of defense response | | | | | 5 | 1.269036 | 0.091929 | 2.913759 | 1 | 0.596922 | 81.27283 |
| GOTERM_BP_FAT | GO:0050729~positive regulation of inflammatory response | | | | | 3 | 0.761421 | 0.155463 | 4.254088 | 1 | 0.738972 | 94.68818 |
|  |  | | | | |  |  |  |  |  |  |  |
| Annotation Cluster 35 | Enrichment Score: 1.150869978421226 | | | | | | | |  |  |  |  |
| Category | Term | | | | | Count | % | PValue | Fold Enrichment | Bonferroni | Benjamini | FDR |
| GOTERM_BP_FAT | GO:0006107~oxaloacetate metabolic process | | | | | 3 | 0.761421 | 0.021753 | 12.76226 | 1 | 0.258159 | 31.75502 |
| GOTERM_BP_FAT | GO:0032868~response to insulin stimulus | | | | | 6 | 1.522843 | 0.085768 | 2.552453 | 1 | 0.575134 | 78.93867 |
| GOTERM_BP_FAT | GO:0043648~dicarboxylic acid metabolic process | | | | | 3 | 0.761421 | 0.189033 | 3.753607 | 1 | 0.793902 | 97.37439 |
|  |  | | | | |  |  |  |  |  |  |  |
| Annotation Cluster 36 | Enrichment Score: 1.1362978966938158 | | | | | | | |  |  |  |  |
| Category | Term | | | | | Count | % | PValue | Fold Enrichment | Bonferroni | Benjamini | FDR |
| GOTERM_BP_FAT | GO:0044242~cellular lipid catabolic process | | | | | 6 | 1.522843 | 0.032762 | 3.358491 | 1 | 0.340386 | 43.93551 |
| GOTERM_BP_FAT | GO:0019395~fatty acid oxidation | | | | | 4 | 1.015228 | 0.062724 | 4.363167 | 1 | 0.493782 | 67.54441 |
| GOTERM_BP_FAT | GO:0034440~lipid oxidation | | | | | 4 | 1.015228 | 0.062724 | 4.363167 | 1 | 0.493782 | 67.54441 |
| GOTERM_BP_FAT | GO:0030258~lipid modification | | | | | 5 | 1.269036 | 0.078352 | 3.082673 | 1 | 0.555464 | 75.76534 |
| GOTERM_BP_FAT | GO:0009062~fatty acid catabolic process | | | | | 3 | 0.761421 | 0.206167 | 3.545073 | 1 | 0.815109 | 98.18816 |
|  |  | | | | |  |  |  |  |  |  |  |
| Annotation Cluster 37 | Enrichment Score: 1.1356679063991806 | | | | | | | |  |  |  |  |
| Category | Term | | | | | Count | % | PValue | Fold Enrichment | Bonferroni | Benjamini | FDR |
| GOTERM_BP_FAT | GO:0050746~regulation of lipoprotein metabolic process | | | | | 3 | 0.761421 | 0.01396 | 15.95283 | 1 | 0.195841 | 21.66865 |
| GOTERM_BP_FAT | GO:0032368~regulation of lipid transport | | | | | 4 | 1.015228 | 0.032445 | 5.672117 | 1 | 0.339246 | 43.61533 |
| GOTERM_BP_FAT | GO:0032371~regulation of sterol transport | | | | | 3 | 0.761421 | 0.078848 | 6.381132 | 1 | 0.556198 | 75.9908 |
| GOTERM_BP_FAT | GO:0032374~regulation of cholesterol transport | | | | | 3 | 0.761421 | 0.078848 | 6.381132 | 1 | 0.556198 | 75.9908 |
| GOTERM_BP_FAT | GO:0019637~organophosphate metabolic process | | | | | 8 | 2.030457 | 0.189736 | 1.701635 | 1 | 0.793009 | 97.41364 |
| GOTERM_BP_FAT | GO:0006644~phospholipid metabolic process | | | | | 7 | 1.77665 | 0.287227 | 1.567296 | 1 | 0.887339 | 99.72106 |
|  |  | | | | |  |  |  |  |  |  |  |
| Annotation Cluster 38 | Enrichment Score: 1.1300392181744578 | | | | | | | |  |  |  |  |
| Category | Term | | | | | Count | % | PValue | Fold Enrichment | Bonferroni | Benjamini | FDR |
| GOTERM_BP_FAT | GO:0043603~cellular amide metabolic process | | | | | 8 | 2.030457 | 3.14E-04 | 6.077269 | 0.497116 | 0.009246 | 0.54477 |
| GOTERM_BP_FAT | GO:0006732~coenzyme metabolic process | | | | | 8 | 2.030457 | 0.068601 | 2.22436 | 1 | 0.519438 | 70.90384 |
| GOTERM_BP_FAT | GO:0009820~alkaloid metabolic process | | | | | 4 | 1.015228 | 0.070748 | 4.15033 | 1 | 0.52907 | 72.04737 |
| GOTERM_BP_FAT | GO:0019674~NAD metabolic process | | | | | 3 | 0.761421 | 0.115487 | 5.104906 | 1 | 0.658033 | 88.13781 |
| GOTERM_BP_FAT | GO:0006769~nicotinamide metabolic process | | | | | 3 | 0.761421 | 0.240856 | 3.190566 | 1 | 0.851366 | 99.16626 |
| GOTERM_BP_FAT | GO:0046496~nicotinamide nucleotide metabolic process | | | | | 3 | 0.761421 | 0.240856 | 3.190566 | 1 | 0.851366 | 99.16626 |
| GOTERM_BP_FAT | GO:0019362~pyridine nucleotide metabolic process | | | | | 3 | 0.761421 | 0.258312 | 3.038634 | 1 | 0.864348 | 99.44343 |
| GOTERM_BP_FAT | GO:0006733~oxidoreduction coenzyme metabolic process | | | | | 3 | 0.761421 | 0.345114 | 2.454282 | 1 | 0.925125 | 99.93595 |
|  |  | | | | |  |  |  |  |  |  |  |
| Annotation Cluster 39 | Enrichment Score: 1.056386465374246 | | | | | | | |  |  |  |  |
| Category | Term | | | | | Count | % | PValue | Fold Enrichment | Bonferroni | Benjamini | FDR |
| GOTERM_BP_FAT | GO:0051591~response to cAMP | | | | | 6 | 1.522843 | 0.002861 | 6.077269 | 0.998093 | 0.057367 | 4.854764 |
| GOTERM_BP_FAT | GO:0051412~response to corticosterone stimulus | | | | | 3 | 0.761421 | 0.052913 | 7.976415 | 1 | 0.458051 | 61.10895 |
| GOTERM_BP_FAT | GO:0051385~response to mineralocorticoid stimulus | | | | | 3 | 0.761421 | 0.078848 | 6.381132 | 1 | 0.556198 | 75.9908 |
| GOTERM_BP_FAT | GO:0009416~response to light stimulus | | | | | 4 | 1.015228 | 0.631401 | 1.233069 | 1 | 0.993442 | 100 |
| GOTERM_BP_FAT | GO:0009314~response to radiation | | | | | 5 | 1.269036 | 0.693338 | 1.063522 | 1 | 0.997146 | 100 |
|  |  | | | | |  |  |  |  |  |  |  |
| Annotation Cluster 40 | Enrichment Score: 1.0521861894871996 | | | | | | | |  |  |  |  |
| Category | Term | | | | | Count | % | PValue | Fold Enrichment | Bonferroni | Benjamini | FDR |
| GOTERM_BP_FAT | GO:0032103~positive regulation of response to external stimulus | | | | | 6 | 1.522843 | 0.016919 | 3.988208 | 1 | 0.217611 | 25.65298 |
| GOTERM_BP_FAT | GO:0030334~regulation of cell migration | | | | | 9 | 2.284264 | 0.045642 | 2.265491 | 1 | 0.419117 | 55.58304 |
| GOTERM_BP_FAT | GO:0030335~positive regulation of cell migration | | | | | 6 | 1.522843 | 0.05786 | 2.867925 | 1 | 0.475335 | 64.49095 |
| GOTERM_BP_FAT | GO:0051272~positive regulation of cell motion | | | | | 6 | 1.522843 | 0.080245 | 2.604544 | 1 | 0.557944 | 76.61593 |
| GOTERM_BP_FAT | GO:0040017~positive regulation of locomotion | | | | | 6 | 1.522843 | 0.080245 | 2.604544 | 1 | 0.557944 | 76.61593 |
| GOTERM_BP_FAT | GO:0040012~regulation of locomotion | | | | | 9 | 2.284264 | 0.082056 | 1.994104 | 1 | 0.564752 | 77.40305 |
| GOTERM_BP_FAT | GO:0051270~regulation of cell motion | | | | | 9 | 2.284264 | 0.0845 | 1.983772 | 1 | 0.571066 | 78.42559 |
| GOTERM_BP_FAT | GO:0050921~positive regulation of chemotaxis | | | | | 3 | 0.761421 | 0.147262 | 4.400781 | 1 | 0.722039 | 93.71721 |
| GOTERM_BP_FAT | GO:0050920~regulation of chemotaxis | | | | | 3 | 0.761421 | 0.163749 | 4.116859 | 1 | 0.752445 | 95.52434 |
| GOTERM_BP_FAT | GO:0048520~positive regulation of behavior | | | | | 3 | 0.761421 | 0.189033 | 3.753607 | 1 | 0.793902 | 97.37439 |
| GOTERM_BP_FAT | GO:0050795~regulation of behavior | | | | | 3 | 0.761421 | 0.293235 | 2.774405 | 1 | 0.889796 | 99.7592 |
|  |  | | | | |  |  |  |  |  |  |  |
| Annotation Cluster 41 | Enrichment Score: 1.0280445057293235 | | | | | | | |  |  |  |  |
| Category | Term | | | | | Count | % | PValue | Fold Enrichment | Bonferroni | Benjamini | FDR |
| GOTERM_BP_FAT | GO:0032570~response to progesterone stimulus | | | | | 4 | 1.015228 | 0.010811 | 8.508176 | 1 | 0.161393 | 17.20732 |
| GOTERM_BP_FAT | GO:0009612~response to mechanical stimulus | | | | | 5 | 1.269036 | 0.041947 | 3.798293 | 1 | 0.402321 | 52.49908 |
| GOTERM_BP_FAT | GO:0009628~response to abiotic stimulus | | | | | 12 | 3.045685 | 0.245642 | 1.387203 | 1 | 0.855096 | 99.25301 |
| GOTERM_BP_FAT | GO:0009314~response to radiation | | | | | 5 | 1.269036 | 0.693338 | 1.063522 | 1 | 0.997146 | 100 |
|  |  | | | | |  |  |  |  |  |  |  |
| Annotation Cluster 42 | Enrichment Score: 1.024924023183794 | | | | | | | |  |  |  |  |
| Category | Term | | | | | Count | % | PValue | Fold Enrichment | Bonferroni | Benjamini | FDR |
| GOTERM_BP_FAT | GO:0046942~carboxylic acid transport | | | | | 8 | 2.030457 | 0.057841 | 2.31515 | 1 | 0.476903 | 64.47867 |
| GOTERM_BP_FAT | GO:0015849~organic acid transport | | | | | 8 | 2.030457 | 0.059557 | 2.299507 | 1 | 0.480443 | 65.58561 |
| GOTERM_BP_FAT | GO:0015837~amine transport | | | | | 6 | 1.522843 | 0.143815 | 2.163096 | 1 | 0.715523 | 93.26118 |
| GOTERM_BP_FAT | GO:0006865~amino acid transport | | | | | 5 | 1.269036 | 0.160446 | 2.363382 | 1 | 0.748455 | 95.2071 |
|  |  | | | | |  |  |  |  |  |  |  |
| Annotation Cluster 43 | Enrichment Score: 1.0087384209102765 | | | | | | | |  |  |  |  |
| Category | Term | | | | | Count | % | PValue | Fold Enrichment | Bonferroni | Benjamini | FDR |
| GOTERM_BP_FAT | GO:0045861~negative regulation of proteolysis | | | | | 4 | 1.015228 | 0.014111 | 7.734706 | 1 | 0.196493 | 21.87576 |
| GOTERM_BP_FAT | GO:0030162~regulation of proteolysis | | | | | 5 | 1.269036 | 0.03529 | 4.013291 | 1 | 0.356755 | 46.4274 |
| GOTERM_BP_FAT | GO:0032269~negative regulation of cellular protein metabolic process | | | | | 6 | 1.522843 | 0.414591 | 1.418029 | 1 | 0.954423 | 99.99087 |
| GOTERM_BP_FAT | GO:0051248~negative regulation of protein metabolic process | | | | | 6 | 1.522843 | 0.446917 | 1.364948 | 1 | 0.963216 | 99.9966 |
|  |  | | | | |  |  |  |  |  |  |  |
| Annotation Cluster 44 | Enrichment Score: 0.9776682729621494 | | | | | | | |  |  |  |  |
| Category | Term | | | | | Count | % | PValue | Fold Enrichment | Bonferroni | Benjamini | FDR |
| GOTERM_BP_FAT | GO:0001525~angiogenesis | | | | | 8 | 2.030457 | 0.059557 | 2.299507 | 1 | 0.480443 | 65.58561 |
| GOTERM_BP_FAT | GO:0001568~blood vessel development | | | | | 10 | 2.538071 | 0.122784 | 1.736362 | 1 | 0.673274 | 89.72778 |
| GOTERM_BP_FAT | GO:0048514~blood vessel morphogenesis | | | | | 9 | 2.284264 | 0.123365 | 1.81454 | 1 | 0.672274 | 89.8454 |
| GOTERM_BP_FAT | GO:0001944~vasculature development | | | | | 10 | 2.538071 | 0.136163 | 1.694856 | 1 | 0.701035 | 92.13483 |
|  |  | | | | |  |  |  |  |  |  |  |
| Annotation Cluster 45 | Enrichment Score: 0.9276501912783319 | | | | | | | |  |  |  |  |
| Category | Term | | | | | Count | % | PValue | Fold Enrichment | Bonferroni | Benjamini | FDR |
| GOTERM_BP_FAT | GO:0002683~negative regulation of immune system process | | | | | 6 | 1.522843 | 0.045216 | 3.075244 | 1 | 0.417768 | 55.23689 |
| GOTERM_BP_FAT | GO:0002698~negative regulation of immune effector process | | | | | 3 | 0.761421 | 0.052913 | 7.976415 | 1 | 0.458051 | 61.10895 |
| GOTERM_BP_FAT | GO:0002697~regulation of immune effector process | | | | | 3 | 0.761421 | 0.688963 | 1.263591 | 1 | 0.996979 | 100 |
|  |  | | | | |  |  |  |  |  |  |  |
| Annotation Cluster 46 | Enrichment Score: 0.9213811765977211 | | | | | | | |  |  |  |  |
| Category | Term | | | | | Count | % | PValue | Fold Enrichment | Bonferroni | Benjamini | FDR |
| GOTERM_BP_FAT | GO:0006029~proteoglycan metabolic process | | | | | 5 | 1.269036 | 0.017812 | 4.946614 | 1 | 0.223898 | 26.81752 |
| GOTERM_BP_FAT | GO:0050654~chondroitin sulfate proteoglycan metabolic process | | | | | 3 | 0.761421 | 0.065444 | 7.090147 | 1 | 0.505674 | 69.1425 |
| GOTERM_BP_FAT | GO:0030166~proteoglycan biosynthetic process | | | | | 3 | 0.761421 | 0.123257 | 4.908563 | 1 | 0.67335 | 89.82354 |
| GOTERM_BP_FAT | GO:0009100~glycoprotein metabolic process | | | | | 7 | 1.77665 | 0.338098 | 1.474189 | 1 | 0.921206 | 99.92293 |
| GOTERM_BP_FAT | GO:0009101~glycoprotein biosynthetic process | | | | | 5 | 1.269036 | 0.508927 | 1.34623 | 1 | 0.976813 | 99.99957 |
|  |  | | | | |  |  |  |  |  |  |  |
| Annotation Cluster 47 | Enrichment Score: 0.8846062010135242 | | | | | | | |  |  |  |  |
| Category | Term | | | | | Count | % | PValue | Fold Enrichment | Bonferroni | Benjamini | FDR |
| GOTERM_BP_FAT | GO:0051240~positive regulation of multicellular organismal process | | | | | 16 | 4.060914 | 6.47E-04 | 2.789566 | 0.756983 | 0.017313 | 1.117878 |
| GOTERM_BP_FAT | GO:0010740~positive regulation of protein kinase cascade | | | | | 9 | 2.284264 | 0.043065 | 2.292622 | 1 | 0.408939 | 53.45289 |
| GOTERM_BP_FAT | GO:0001501~skeletal system development | | | | | 13 | 3.299492 | 0.071637 | 1.733641 | 1 | 0.53201 | 72.50841 |
| GOTERM_BP_FAT | GO:0001649~osteoblast differentiation | | | | | 4 | 1.015228 | 0.074925 | 4.051512 | 1 | 0.543673 | 74.15146 |
| GOTERM_BP_FAT | GO:0042327~positive regulation of phosphorylation | | | | | 6 | 1.522843 | 0.077558 | 2.631395 | 1 | 0.553284 | 75.40018 |
| GOTERM_BP_FAT | GO:0045937~positive regulation of phosphate metabolic process | | | | | 6 | 1.522843 | 0.085768 | 2.552453 | 1 | 0.575134 | 78.93867 |
| GOTERM_BP_FAT | GO:0010562~positive regulation of phosphorus metabolic process | | | | | 6 | 1.522843 | 0.085768 | 2.552453 | 1 | 0.575134 | 78.93867 |
| GOTERM_BP_FAT | GO:0050731~positive regulation of peptidyl-tyrosine phosphorylation | | | | | 4 | 1.015228 | 0.097361 | 3.6205 | 1 | 0.611243 | 83.12657 |
| GOTERM_BP_FAT | GO:0032270~positive regulation of cellular protein metabolic process | | | | | 10 | 2.538071 | 0.097742 | 1.825789 | 1 | 0.611206 | 83.2498 |
| GOTERM_BP_FAT | GO:0043410~positive regulation of MAPKKK cascade | | | | | 4 | 1.015228 | 0.102141 | 3.545073 | 1 | 0.620629 | 84.61339 |
| GOTERM_BP_FAT | GO:0051247~positive regulation of protein metabolic process | | | | | 10 | 2.538071 | 0.118434 | 1.750654 | 1 | 0.663501 | 88.80607 |
| GOTERM_BP_FAT | GO:0010647~positive regulation of cell communication | | | | | 12 | 3.045685 | 0.150791 | 1.551643 | 1 | 0.728561 | 94.15394 |
| GOTERM_BP_FAT | GO:0001934~positive regulation of protein amino acid phosphorylation | | | | | 5 | 1.269036 | 0.155988 | 2.389937 | 1 | 0.738988 | 94.74527 |
| GOTERM_BP_FAT | GO:0060348~bone development | | | | | 6 | 1.522843 | 0.162356 | 2.075165 | 1 | 0.751691 | 95.39309 |
| GOTERM_BP_FAT | GO:0019220~regulation of phosphate metabolic process | | | | | 16 | 4.060914 | 0.167025 | 1.40341 | 1 | 0.756258 | 95.8194 |
| GOTERM_BP_FAT | GO:0051174~regulation of phosphorus metabolic process | | | | | 16 | 4.060914 | 0.167025 | 1.40341 | 1 | 0.756258 | 95.8194 |
| GOTERM_BP_FAT | GO:0042325~regulation of phosphorylation | | | | | 15 | 3.807107 | 0.205564 | 1.369342 | 1 | 0.815125 | 98.16407 |
| GOTERM_BP_FAT | GO:0050730~regulation of peptidyl-tyrosine phosphorylation | | | | | 4 | 1.015228 | 0.213398 | 2.502405 | 1 | 0.825045 | 98.45443 |
| GOTERM_BP_FAT | GO:0001932~regulation of protein amino acid phosphorylation | | | | | 7 | 1.77665 | 0.220554 | 1.721307 | 1 | 0.831367 | 98.68132 |
| GOTERM_BP_FAT | GO:0010627~regulation of protein kinase cascade | | | | | 9 | 2.284264 | 0.23034 | 1.537622 | 1 | 0.841217 | 98.94119 |
| GOTERM_BP_FAT | GO:0032268~regulation of cellular protein metabolic process | | | | | 15 | 3.807107 | 0.231659 | 1.34623 | 1 | 0.842186 | 98.97227 |
| GOTERM_BP_FAT | GO:0009967~positive regulation of signal transduction | | | | | 10 | 2.538071 | 0.255596 | 1.442064 | 1 | 0.863509 | 99.40695 |
| GOTERM_BP_FAT | GO:0001503~ossification | | | | | 5 | 1.269036 | 0.28363 | 1.849604 | 1 | 0.885098 | 99.69557 |
| GOTERM_BP_FAT | GO:0048660~regulation of smooth muscle cell proliferation | | | | | 3 | 0.761421 | 0.293235 | 2.774405 | 1 | 0.889796 | 99.7592 |
| GOTERM_BP_FAT | GO:0031401~positive regulation of protein modification process | | | | | 6 | 1.522843 | 0.446917 | 1.364948 | 1 | 0.963216 | 99.9966 |
| GOTERM_BP_FAT | GO:0043408~regulation of MAPKKK cascade | | | | | 4 | 1.015228 | 0.472225 | 1.561133 | 1 | 0.969045 | 99.99849 |
| GOTERM_BP_FAT | GO:0031399~regulation of protein modification process | | | | | 8 | 2.030457 | 0.538524 | 1.153651 | 1 | 0.982477 | 99.99985 |
|  |  | | | | |  |  |  |  |  |  |  |
| Annotation Cluster 48 | Enrichment Score: 0.8787495763262201 | | | | | | | |  |  |  |  |
| Category | Term | | | | | Count | % | PValue | Fold Enrichment | Bonferroni | Benjamini | FDR |
| GOTERM_BP_FAT | GO:0016052~carbohydrate catabolic process | | | | | 9 | 2.284264 | 0.004079 | 3.51255 | 0.999868 | 0.077339 | 6.854071 |
| GOTERM_BP_FAT | GO:0044275~cellular carbohydrate catabolic process | | | | | 6 | 1.522843 | 0.049227 | 3.002886 | 1 | 0.437147 | 58.394 |
| GOTERM_BP_FAT | GO:0046164~alcohol catabolic process | | | | | 5 | 1.269036 | 0.122139 | 2.62598 | 1 | 0.672648 | 89.59579 |
| GOTERM_BP_FAT | GO:0006096~glycolysis | | | | | 3 | 0.761421 | 0.30194 | 2.715375 | 1 | 0.896111 | 99.80585 |
| GOTERM_BP_FAT | GO:0006007~glucose catabolic process | | | | | 3 | 0.761421 | 0.395706 | 2.20039 | 1 | 0.947762 | 99.98415 |
| GOTERM_BP_FAT | GO:0019320~hexose catabolic process | | | | | 3 | 0.761421 | 0.483342 | 1.849604 | 1 | 0.971686 | 99.99896 |
| GOTERM_BP_FAT | GO:0046365~monosaccharide catabolic process | | | | | 3 | 0.761421 | 0.498426 | 1.797502 | 1 | 0.974522 | 99.99938 |
|  |  | | | | |  |  |  |  |  |  |  |
| Annotation Cluster 49 | Enrichment Score: 0.861907426774219 | | | | | | | |  |  |  |  |
| Category | Term | | | | | Count | % | PValue | Fold Enrichment | Bonferroni | Benjamini | FDR |
| GOTERM_BP_FAT | GO:0009595~detection of biotic stimulus | | | | | 4 | 1.015228 | 0.00801 | 9.453529 | 1 | 0.129276 | 13.0389 |
| GOTERM_BP_FAT | GO:0031349~positive regulation of defense response | | | | | 5 | 1.269036 | 0.091929 | 2.913759 | 1 | 0.596922 | 81.27283 |
| GOTERM_BP_FAT | GO:0009581~detection of external stimulus | | | | | 5 | 1.269036 | 0.095487 | 2.874384 | 1 | 0.606846 | 82.5077 |
| GOTERM_BP_FAT | GO:0045088~regulation of innate immune response | | | | | 4 | 1.015228 | 0.132654 | 3.151176 | 1 | 0.697767 | 91.56084 |
| GOTERM_BP_FAT | GO:0051606~detection of stimulus | | | | | 6 | 1.522843 | 0.143815 | 2.163096 | 1 | 0.715523 | 93.26118 |
| GOTERM_BP_FAT | GO:0045089~positive regulation of innate immune response | | | | | 3 | 0.761421 | 0.293235 | 2.774405 | 1 | 0.889796 | 99.7592 |
| GOTERM_BP_FAT | GO:0045321~leukocyte activation | | | | | 8 | 2.030457 | 0.339962 | 1.40631 | 1 | 0.922004 | 99.92662 |
| GOTERM_BP_FAT | GO:0002757~immune response-activating signal transduction | | | | | 3 | 0.761421 | 0.345114 | 2.454282 | 1 | 0.925125 | 99.93595 |
| GOTERM_BP_FAT | GO:0002764~immune response-regulating signal transduction | | | | | 3 | 0.761421 | 0.379024 | 2.278976 | 1 | 0.941004 | 99.97457 |
|  |  | | | | |  |  |  |  |  |  |  |
| Annotation Cluster 50 | Enrichment Score: 0.860017104995342 |  |  |  | | | | |  |  |  |  |
| Category | Term | | | | | Count | % | PValue | Fold Enrichment | Bonferroni | Benjamini | FDR |
| GOTERM_BP_FAT | GO:0032760~positive regulation of tumor necrosis factor production | | | | | 3 | 0.761421 | 0.04701 | 8.508176 | 1 | 0.42535 | 56.67631 |
| GOTERM_BP_FAT | GO:0001817~regulation of cytokine production | | | | | 8 | 2.030457 | 0.133516 | 1.88026 | 1 | 0.698899 | 91.70551 |
| GOTERM_BP_FAT | GO:0032680~regulation of tumor necrosis factor production | | | | | 3 | 0.761421 | 0.163749 | 4.116859 | 1 | 0.752445 | 95.52434 |
| GOTERM_BP_FAT | GO:0001819~positive regulation of cytokine production | | | | | 4 | 1.015228 | 0.353203 | 1.890706 | 1 | 0.929052 | 99.94839 |
|  |  | | | | |  |  |  |  |  |  |  |
| Annotation Cluster 51 | Enrichment Score: 0.8586922335431073 |  |  |  | | | | |  |  |  |  |
| Category | Term | | | | | Count | % | PValue | Fold Enrichment | Bonferroni | Benjamini | FDR |
| GOTERM_BP_FAT | GO:0008228~opsonization | | | | | 3 | 0.761421 | 0.007714 | 21.27044 | 1 | 0.127614 | 12.58742 |
| GOTERM_BP_FAT | GO:0006909~phagocytosis | | | | | 5 | 1.269036 | 0.025657 | 4.431342 | 1 | 0.291326 | 36.33476 |
| GOTERM_BP_FAT | GO:0006897~endocytosis | | | | | 8 | 2.030457 | 0.259316 | 1.546941 | 1 | 0.864745 | 99.45637 |
| GOTERM_BP_FAT | GO:0010324~membrane invagination | | | | | 8 | 2.030457 | 0.259316 | 1.546941 | 1 | 0.864745 | 99.45637 |
| GOTERM_BP_FAT | GO:0016044~membrane organization | | | | | 10 | 2.538071 | 0.538163 | 1.116559 | 1 | 0.982575 | 99.99985 |
| GOTERM_BP_FAT | GO:0016192~vesicle-mediated transport | | | | | 8 | 2.030457 | 0.983473 | 0.590846 | 1 | 1 | 100 |
|  |  | | | | |  |  |  |  |  |  |  |
| Annotation Cluster 52 | Enrichment Score: 0.8175294994146795 |  |  |  | | | | |  |  |  |  |
| Category | Term | | | | | Count | % | PValue | Fold Enrichment | Bonferroni | Benjamini | FDR |
| GOTERM_BP_FAT | GO:0015980~energy derivation by oxidation of organic compounds | | | | | 8 | 2.030457 | 0.052881 | 2.363382 | 1 | 0.459558 | 61.0858 |
| GOTERM_BP_FAT | GO:0006112~energy reserve metabolic process | | | | | 4 | 1.015228 | 0.079208 | 3.957291 | 1 | 0.556287 | 76.1537 |
| GOTERM_BP_FAT | GO:0005977~glycogen metabolic process | | | | | 3 | 0.761421 | 0.197577 | 3.646361 | 1 | 0.805373 | 97.81565 |
| GOTERM_BP_FAT | GO:0044042~glucan metabolic process | | | | | 3 | 0.761421 | 0.206167 | 3.545073 | 1 | 0.815109 | 98.18816 |
| GOTERM_BP_FAT | GO:0006073~cellular glucan metabolic process | | | | | 3 | 0.761421 | 0.206167 | 3.545073 | 1 | 0.815109 | 98.18816 |
| GOTERM_BP_FAT | GO:0044264~cellular polysaccharide metabolic process | | | | | 3 | 0.761421 | 0.353653 | 2.407974 | 1 | 0.928309 | 99.94901 |
|  |  | | | | |  |  |  |  |  |  |  |
| Annotation Cluster 53 | Enrichment Score: 0.8051171174586559 |  |  |  | | | | |  |  |  |  |
| Category | Term | | | | | Count | % | PValue | Fold Enrichment | Bonferroni | Benjamini | FDR |
| GOTERM_BP_FAT | GO:0010565~regulation of cellular ketone metabolic process | | | | | 5 | 1.269036 | 0.044313 | 3.731656 | 1 | 0.416366 | 54.49619 |
| GOTERM_BP_FAT | GO:0045834~positive regulation of lipid metabolic process | | | | | 4 | 1.015228 | 0.092675 | 3.699207 | 1 | 0.598457 | 81.53845 |
| GOTERM_BP_FAT | GO:0019217~regulation of fatty acid metabolic process | | | | | 4 | 1.015228 | 0.107013 | 3.472725 | 1 | 0.63724 | 86.00108 |
| GOTERM_BP_FAT | GO:0019216~regulation of lipid metabolic process | | | | | 5 | 1.269036 | 0.267978 | 1.899146 | 1 | 0.872565 | 99.55684 |
| GOTERM_BP_FAT | GO:0051130~positive regulation of cellular component organization | | | | | 4 | 1.015228 | 0.800538 | 0.94013 | 1 | 0.999437 | 100 |
|  |  | | | | |  |  |  |  |  |  |  |
| Annotation Cluster 54 | Enrichment Score: 0.7427249192363504 |  |  |  | | | | |  |  |  |  |
| Category | Term | | | | | Count | % | PValue | Fold Enrichment | Bonferroni | Benjamini | FDR |
| GOTERM_BP_FAT | GO:0030335~positive regulation of cell migration | | | | | 6 | 1.522843 | 0.05786 | 2.867925 | 1 | 0.475335 | 64.49095 |
| GOTERM_BP_FAT | GO:0051272~positive regulation of cell motion | | | | | 6 | 1.522843 | 0.080245 | 2.604544 | 1 | 0.557944 | 76.61593 |
| GOTERM_BP_FAT | GO:0040017~positive regulation of locomotion | | | | | 6 | 1.522843 | 0.080245 | 2.604544 | 1 | 0.557944 | 76.61593 |
| GOTERM_BP_FAT | GO:0045740~positive regulation of DNA replication | | | | | 3 | 0.761421 | 0.139154 | 4.557951 | 1 | 0.705418 | 92.59467 |
| GOTERM_BP_FAT | GO:0051054~positive regulation of DNA metabolic process | | | | | 3 | 0.761421 | 0.379024 | 2.278976 | 1 | 0.941004 | 99.97457 |
| GOTERM_BP_FAT | GO:0006275~regulation of DNA replication | | | | | 3 | 0.761421 | 0.428429 | 2.05843 | 1 | 0.958249 | 99.99398 |
| GOTERM_BP_FAT | GO:0051052~regulation of DNA metabolic process | | | | | 3 | 0.761421 | 0.751041 | 1.119497 | 1 | 0.998781 | 100 |
|  |  | | | | |  |  |  |  |  |  |  |
| Annotation Cluster 55 | Enrichment Score: 0.7006281139814381 |  |  |  | | | | |  |  |  |  |
| Category | Term | | | | | Count | % | PValue | Fold Enrichment | Bonferroni | Benjamini | FDR |
| GOTERM_BP_FAT | GO:0015758~glucose transport | | | | | 3 | 0.761421 | 0.147262 | 4.400781 | 1 | 0.722039 | 93.71721 |
| GOTERM_BP_FAT | GO:0008645~hexose transport | | | | | 3 | 0.761421 | 0.155463 | 4.254088 | 1 | 0.738972 | 94.68818 |
| GOTERM_BP_FAT | GO:0015749~monosaccharide transport | | | | | 3 | 0.761421 | 0.163749 | 4.116859 | 1 | 0.752445 | 95.52434 |
| GOTERM_BP_FAT | GO:0008643~carbohydrate transport | | | | | 3 | 0.761421 | 0.420333 | 2.092174 | 1 | 0.955865 | 99.99231 |
|  |  | | | | |  |  |  |  |  |  |  |
| Annotation Cluster 56 | Enrichment Score: 0.6962195297364305 |  |  |  | | | | |  |  |  |  |
| Category | Term | | | | | Count | % | PValue | Fold Enrichment | Bonferroni | Benjamini | FDR |
| GOTERM_BP_FAT | GO:0031644~regulation of neurological system process | | | | | 7 | 1.77665 | 0.150417 | 1.946315 | 1 | 0.728902 | 94.10913 |
| GOTERM_BP_FAT | GO:0048167~regulation of synaptic plasticity | | | | | 4 | 1.015228 | 0.189252 | 2.658805 | 1 | 0.7932 | 97.38666 |
| GOTERM_BP_FAT | GO:0048168~regulation of neuronal synaptic plasticity | | | | | 3 | 0.761421 | 0.206167 | 3.545073 | 1 | 0.815109 | 98.18816 |
| GOTERM_BP_FAT | GO:0050804~regulation of synaptic transmission | | | | | 6 | 1.522843 | 0.214564 | 1.876804 | 1 | 0.825913 | 98.49377 |
| GOTERM_BP_FAT | GO:0051969~regulation of transmission of nerve impulse | | | | | 6 | 1.522843 | 0.262293 | 1.736362 | 1 | 0.867514 | 99.49311 |
|  |  | | | | |  |  |  |  |  |  |  |
| Annotation Cluster 57 | Enrichment Score: 0.6489206681517642 |  |  |  | | | | |  |  |  |  |
| Category | Term | | | | | Count | % | PValue | Fold Enrichment | Bonferroni | Benjamini | FDR |
| GOTERM_BP_FAT | GO:0045637~regulation of myeloid cell differentiation | | | | | 6 | 1.522843 | 0.024003 | 3.646361 | 1 | 0.27664 | 34.43071 |
| GOTERM_BP_FAT | GO:0002761~regulation of myeloid leukocyte differentiation | | | | | 4 | 1.015228 | 0.070748 | 4.15033 | 1 | 0.52907 | 72.04737 |
| GOTERM_BP_FAT | GO:0045670~regulation of osteoclast differentiation | | | | | 3 | 0.761421 | 0.093015 | 5.801029 | 1 | 0.598298 | 81.65837 |
| GOTERM_BP_FAT | GO:0051090~regulation of transcription factor activity | | | | | 5 | 1.269036 | 0.222149 | 2.065091 | 1 | 0.832842 | 98.72742 |
| GOTERM_BP_FAT | GO:0043433~negative regulation of transcription factor activity | | | | | 3 | 0.761421 | 0.284515 | 2.836059 | 1 | 0.885281 | 99.70203 |
| GOTERM_BP_FAT | GO:0051098~regulation of binding | | | | | 6 | 1.522843 | 0.289301 | 1.66827 | 1 | 0.887999 | 99.73483 |
| GOTERM_BP_FAT | GO:0042108~positive regulation of cytokine biosynthetic process | | | | | 3 | 0.761421 | 0.310627 | 2.658805 | 1 | 0.903343 | 99.8438 |
| GOTERM_BP_FAT | GO:0051101~regulation of DNA binding | | | | | 5 | 1.269036 | 0.315302 | 1.757888 | 1 | 0.906754 | 99.86122 |
| GOTERM_BP_FAT | GO:0043392~negative regulation of DNA binding | | | | | 3 | 0.761421 | 0.336538 | 2.502405 | 1 | 0.920624 | 99.91971 |
| GOTERM_BP_FAT | GO:0043123~positive regulation of I-kappaB kinase/NF-kappaB cascade | | | | | 4 | 1.015228 | 0.397882 | 1.754263 | 1 | 0.948038 | 99.98512 |
| GOTERM_BP_FAT | GO:0051100~negative regulation of binding | | | | | 3 | 0.761421 | 0.40397 | 2.163096 | 1 | 0.950632 | 99.98753 |
| GOTERM_BP_FAT | GO:0043122~regulation of I-kappaB kinase/NF-kappaB cascade | | | | | 4 | 1.015228 | 0.4601 | 1.590313 | 1 | 0.96671 | 99.99776 |
| GOTERM_BP_FAT | GO:0042035~regulation of cytokine biosynthetic process | | | | | 3 | 0.761421 | 0.520519 | 1.72463 | 1 | 0.97918 | 99.99972 |
|  |  | | | | |  |  |  |  |  |  |  |
| Annotation Cluster 58 | Enrichment Score: 0.6399968330984095 |  |  |  | | | | |  |  |  |  |
| Category | Term | | | | | Count | % | PValue | Fold Enrichment | Bonferroni | Benjamini | FDR |
| GOTERM_BP_FAT | GO:0006916~anti-apoptosis | | | | | 10 | 2.538071 | 0.053818 | 2.065091 | 1 | 0.460438 | 61.74966 |
| GOTERM_BP_FAT | GO:0043066~negative regulation of apoptosis | | | | | 14 | 3.553299 | 0.073141 | 1.682408 | 1 | 0.536375 | 73.27162 |
| GOTERM_BP_FAT | GO:0043069~negative regulation of programmed cell death | | | | | 14 | 3.553299 | 0.079924 | 1.658976 | 1 | 0.558049 | 76.47367 |
| GOTERM_BP_FAT | GO:0060548~negative regulation of cell death | | | | | 14 | 3.553299 | 0.082615 | 1.654368 | 1 | 0.565713 | 77.64086 |
| GOTERM_BP_FAT | GO:0042981~regulation of apoptosis | | | | | 24 | 6.091371 | 0.1875 | 1.269877 | 1 | 0.793207 | 97.28681 |
| GOTERM_BP_FAT | GO:0043067~regulation of programmed cell death | | | | | 24 | 6.091371 | 0.200053 | 1.257366 | 1 | 0.808721 | 97.92981 |
| GOTERM_BP_FAT | GO:0010941~regulation of cell death | | | | | 24 | 6.091371 | 0.204394 | 1.252738 | 1 | 0.814172 | 98.11655 |
| GOTERM_BP_FAT | GO:0043065~positive regulation of apoptosis | | | | | 11 | 2.791878 | 0.555286 | 1.088255 | 1 | 0.984968 | 99.99992 |
| GOTERM_BP_FAT | GO:0043068~positive regulation of programmed cell death | | | | | 11 | 2.791878 | 0.564212 | 1.080715 | 1 | 0.986189 | 99.99995 |
| GOTERM_BP_FAT | GO:0010942~positive regulation of cell death | | | | | 11 | 2.791878 | 0.570122 | 1.075746 | 1 | 0.986995 | 99.99996 |
| GOTERM_BP_FAT | GO:0006917~induction of apoptosis | | | | | 7 | 1.77665 | 0.764899 | 0.930582 | 1 | 0.999002 | 100 |
| GOTERM_BP_FAT | GO:0012502~induction of programmed cell death | | | | | 7 | 1.77665 | 0.767473 | 0.927683 | 1 | 0.999024 | 100 |
|  |  | | | | |  |  |  |  |  |  |  |
| Annotation Cluster 59 | Enrichment Score: 0.6373911680909027 |  |  |  | | | | |  |  |  |  |
| Category | Term | | | | | Count | % | PValue | Fold Enrichment | Bonferroni | Benjamini | FDR |
| GOTERM_BP_FAT | GO:0030324~lung development | | | | | 5 | 1.269036 | 0.202498 | 2.148529 | 1 | 0.811948 | 98.03702 |
| GOTERM_BP_FAT | GO:0030323~respiratory tube development | | | | | 5 | 1.269036 | 0.217188 | 2.085337 | 1 | 0.828086 | 98.57883 |
| GOTERM_BP_FAT | GO:0060541~respiratory system development | | | | | 5 | 1.269036 | 0.247371 | 1.969485 | 1 | 0.855616 | 99.28221 |
| GOTERM_BP_FAT | GO:0035295~tube development | | | | | 8 | 2.030457 | 0.259316 | 1.546941 | 1 | 0.864745 | 99.45637 |
|  |  | | | | |  |  |  |  |  |  |  |
| Annotation Cluster 60 | Enrichment Score: 0.6067868374750447 |  |  |  | | | | |  |  |  |  |
| Category | Term | | | | | Count | % | PValue | Fold Enrichment | Bonferroni | Benjamini | FDR |
| GOTERM_BP_FAT | GO:0009743~response to carbohydrate stimulus | | | | | 4 | 1.015228 | 0.165881 | 2.836059 | 1 | 0.754882 | 95.71854 |
| GOTERM_BP_FAT | GO:0009746~response to hexose stimulus | | | | | 3 | 0.761421 | 0.30194 | 2.715375 | 1 | 0.896111 | 99.80585 |
| GOTERM_BP_FAT | GO:0034284~response to monosaccharide stimulus | | | | | 3 | 0.761421 | 0.30194 | 2.715375 | 1 | 0.896111 | 99.80585 |
|  |  | | | | |  |  |  |  |  |  |  |
| Annotation Cluster 61 | Enrichment Score: 0.5456365239839726 |  |  |  | | | | |  |  |  |  |
| Category | Term | | | | | Count | % | PValue | Fold Enrichment | Bonferroni | Benjamini | FDR |
| GOTERM_BP_FAT | GO:0051051~negative regulation of transport | | | | | 7 | 1.77665 | 0.097809 | 2.205823 | 1 | 0.609933 | 83.27149 |
| GOTERM_BP_FAT | GO:0051048~negative regulation of secretion | | | | | 4 | 1.015228 | 0.138019 | 3.093882 | 1 | 0.704935 | 92.42321 |
| GOTERM_BP_FAT | GO:0060341~regulation of cellular localization | | | | | 6 | 1.522843 | 0.694331 | 1.029215 | 1 | 0.997116 | 100 |
| GOTERM_BP_FAT | GO:0051046~regulation of secretion | | | | | 5 | 1.269036 | 0.70076 | 1.052992 | 1 | 0.997297 | 100 |
|  |  | | | | |  |  |  |  |  |  |  |
| Annotation Cluster 62 | Enrichment Score: 0.5386893547214295 |  |  |  | | | | |  |  |  |  |
| Category | Term | | | | | Count | % | PValue | Fold Enrichment | Bonferroni | Benjamini | FDR |
| GOTERM_BP_FAT | GO:0002683~negative regulation of immune system process | | | | | 6 | 1.522843 | 0.045216 | 3.075244 | 1 | 0.417768 | 55.23689 |
| GOTERM_BP_FAT | GO:0001817~regulation of cytokine production | | | | | 8 | 2.030457 | 0.133516 | 1.88026 | 1 | 0.698899 | 91.70551 |
| GOTERM_BP_FAT | GO:0001818~negative regulation of cytokine production | | | | | 3 | 0.761421 | 0.223459 | 3.358491 | 1 | 0.833868 | 98.76413 |
| GOTERM_BP_FAT | GO:0050865~regulation of cell activation | | | | | 7 | 1.77665 | 0.228111 | 1.701635 | 1 | 0.838906 | 98.88664 |
| GOTERM_BP_FAT | GO:0002694~regulation of leukocyte activation | | | | | 6 | 1.522843 | 0.349225 | 1.537622 | 1 | 0.926893 | 99.94259 |
| GOTERM_BP_FAT | GO:0002695~negative regulation of leukocyte activation | | | | | 3 | 0.761421 | 0.38739 | 2.238994 | 1 | 0.944708 | 99.97991 |
| GOTERM_BP_FAT | GO:0050866~negative regulation of cell activation | | | | | 3 | 0.761421 | 0.420333 | 2.092174 | 1 | 0.955865 | 99.99231 |
| GOTERM_BP_FAT | GO:0050867~positive regulation of cell activation | | | | | 4 | 1.015228 | 0.484227 | 1.533005 | 1 | 0.971698 | 99.99899 |
| GOTERM_BP_FAT | GO:0051249~regulation of lymphocyte activation | | | | | 4 | 1.015228 | 0.677973 | 1.149754 | 1 | 0.996501 | 100 |
| GOTERM_BP_FAT | GO:0002696~positive regulation of leukocyte activation | | | | | 3 | 0.761421 | 0.714219 | 1.203987 | 1 | 0.997813 | 100 |
|  |  | | | | |  |  |  |  |  |  |  |
| Annotation Cluster 63 | Enrichment Score: 0.5360468590373598 |  |  |  | | | | |  |  |  |  |
| Category | Term | | | | | Count | % | PValue | Fold Enrichment | Bonferroni | Benjamini | FDR |
| GOTERM_BP_FAT | GO:0001558~regulation of cell growth | | | | | 9 | 2.284264 | 0.086493 | 1.973546 | 1 | 0.575177 | 79.22709 |
| GOTERM_BP_FAT | GO:0040008~regulation of growth | | | | | 12 | 3.045685 | 0.178329 | 1.49704 | 1 | 0.775984 | 96.70269 |
| GOTERM_BP_FAT | GO:0008361~regulation of cell size | | | | | 7 | 1.77665 | 0.353308 | 1.445564 | 1 | 0.9286 | 99.94854 |
| GOTERM_BP_FAT | GO:0030308~negative regulation of cell growth | | | | | 4 | 1.015228 | 0.366029 | 1.849604 | 1 | 0.93475 | 99.96356 |
| GOTERM_BP_FAT | GO:0045792~negative regulation of cell size | | | | | 4 | 1.015228 | 0.410508 | 1.718823 | 1 | 0.953319 | 99.9897 |
| GOTERM_BP_FAT | GO:0032535~regulation of cellular component size | | | | | 8 | 2.030457 | 0.451679 | 1.255819 | 1 | 0.964647 | 99.99707 |
| GOTERM_BP_FAT | GO:0045926~negative regulation of growth | | | | | 4 | 1.015228 | 0.478242 | 1.546941 | 1 | 0.970402 | 99.99876 |
|  |  | | | | |  |  |  |  |  |  |  |
| Annotation Cluster 64 | Enrichment Score: 0.535928952817152 |  |  |  | | | | |  |  |  |  |
| Category | Term | | | | | Count | % | PValue | Fold Enrichment | Bonferroni | Benjamini | FDR |
| GOTERM_BP_FAT | GO:0007243~protein kinase cascade | | | | | 14 | 3.553299 | 0.096005 | 1.609655 | 1 | 0.607375 | 82.6808 |
| GOTERM_BP_FAT | GO:0006469~negative regulation of protein kinase activity | | | | | 5 | 1.269036 | 0.147216 | 2.444878 | 1 | 0.723231 | 93.7114 |
| GOTERM_BP_FAT | GO:0033673~negative regulation of kinase activity | | | | | 5 | 1.269036 | 0.160446 | 2.363382 | 1 | 0.748455 | 95.2071 |
| GOTERM_BP_FAT | GO:0051174~regulation of phosphorus metabolic process | | | | | 16 | 4.060914 | 0.167025 | 1.40341 | 1 | 0.756258 | 95.8194 |
| GOTERM_BP_FAT | GO:0019220~regulation of phosphate metabolic process | | | | | 16 | 4.060914 | 0.167025 | 1.40341 | 1 | 0.756258 | 95.8194 |
| GOTERM_BP_FAT | GO:0051348~negative regulation of transferase activity | | | | | 5 | 1.269036 | 0.188121 | 2.215671 | 1 | 0.793275 | 97.32261 |
| GOTERM_BP_FAT | GO:0042325~regulation of phosphorylation | | | | | 15 | 3.807107 | 0.205564 | 1.369342 | 1 | 0.815125 | 98.16407 |
| GOTERM_BP_FAT | GO:0044093~positive regulation of molecular function | | | | | 18 | 4.568528 | 0.215039 | 1.306716 | 1 | 0.825667 | 98.5095 |
| GOTERM_BP_FAT | GO:0043085~positive regulation of catalytic activity | | | | | 16 | 4.060914 | 0.240497 | 1.30895 | 1 | 0.851777 | 99.15938 |
| GOTERM_BP_FAT | GO:0044092~negative regulation of molecular function | | | | | 11 | 2.791878 | 0.257174 | 1.401047 | 1 | 0.863784 | 99.42841 |
| GOTERM_BP_FAT | GO:0032147~activation of protein kinase activity | | | | | 5 | 1.269036 | 0.278396 | 1.865828 | 1 | 0.88181 | 99.65453 |
| GOTERM_BP_FAT | GO:0045859~regulation of protein kinase activity | | | | | 11 | 2.791878 | 0.290557 | 1.356376 | 1 | 0.888547 | 99.74285 |
| GOTERM_BP_FAT | GO:0043549~regulation of kinase activity | | | | | 11 | 2.791878 | 0.327655 | 1.310783 | 1 | 0.914426 | 99.89885 |
| GOTERM_BP_FAT | GO:0051338~regulation of transferase activity | | | | | 11 | 2.791878 | 0.378963 | 1.257929 | 1 | 0.941423 | 99.97453 |
| GOTERM_BP_FAT | GO:0045860~positive regulation of protein kinase activity | | | | | 7 | 1.77665 | 0.425253 | 1.335364 | 1 | 0.957266 | 99.99337 |
| GOTERM_BP_FAT | GO:0000165~MAPKKK cascade | | | | | 6 | 1.522843 | 0.433203 | 1.387203 | 1 | 0.959522 | 99.99479 |
| GOTERM_BP_FAT | GO:0033674~positive regulation of kinase activity | | | | | 7 | 1.77665 | 0.457817 | 1.289118 | 1 | 0.966216 | 99.99759 |
| GOTERM_BP_FAT | GO:0043086~negative regulation of catalytic activity | | | | | 8 | 2.030457 | 0.460914 | 1.228617 | 1 | 0.966701 | 99.99782 |
| GOTERM_BP_FAT | GO:0051347~positive regulation of transferase activity | | | | | 7 | 1.77665 | 0.493723 | 1.240776 | 1 | 0.973695 | 99.99927 |
| GOTERM_BP_FAT | GO:0000187~activation of MAPK activity | | | | | 3 | 0.761421 | 0.57618 | 1.556374 | 1 | 0.987531 | 99.99997 |
| GOTERM_BP_FAT | GO:0043405~regulation of MAP kinase activity | | | | | 4 | 1.015228 | 0.645844 | 1.206833 | 1 | 0.994573 | 100 |
| GOTERM_BP_FAT | GO:0043406~positive regulation of MAP kinase activity | | | | | 3 | 0.761421 | 0.694156 | 1.251202 | 1 | 0.997146 | 100 |
|  |  | | | | |  |  |  |  |  |  |  |
| Annotation Cluster 65 | Enrichment Score: 0.5352475819710396 |  |  |  | | | | |  |  |  |  |
| Category | Term | | | | | Count | % | PValue | Fold Enrichment | Bonferroni | Benjamini | FDR |
| GOTERM_BP_FAT | GO:0010907~positive regulation of glucose metabolic process | | | | | 3 | 0.761421 | 0.072044 | 6.716981 | 1 | 0.53244 | 72.71675 |
| GOTERM_BP_FAT | GO:0010676~positive regulation of cellular carbohydrate metabolic process | | | | | 3 | 0.761421 | 0.078848 | 6.381132 | 1 | 0.556198 | 75.9908 |
| GOTERM_BP_FAT | GO:0045913~positive regulation of carbohydrate metabolic process | | | | | 3 | 0.761421 | 0.078848 | 6.381132 | 1 | 0.556198 | 75.9908 |
| GOTERM_BP_FAT | GO:0010906~regulation of glucose metabolic process | | | | | 3 | 0.761421 | 0.197577 | 3.646361 | 1 | 0.805373 | 97.81565 |
| GOTERM_BP_FAT | GO:0010675~regulation of cellular carbohydrate metabolic process | | | | | 3 | 0.761421 | 0.223459 | 3.358491 | 1 | 0.833868 | 98.76413 |
| GOTERM_BP_FAT | GO:0006109~regulation of carbohydrate metabolic process | | | | | 3 | 0.761421 | 0.232147 | 3.272375 | 1 | 0.841956 | 98.98355 |
| GOTERM_BP_FAT | GO:0031401~positive regulation of protein modification process | | | | | 6 | 1.522843 | 0.446917 | 1.364948 | 1 | 0.963216 | 99.9966 |
| GOTERM_BP_FAT | GO:0031399~regulation of protein modification process | | | | | 8 | 2.030457 | 0.538524 | 1.153651 | 1 | 0.982477 | 99.99985 |
| GOTERM_BP_FAT | GO:0010638~positive regulation of organelle organization | | | | | 3 | 0.761421 | 0.582797 | 1.537622 | 1 | 0.988252 | 99.99997 |
| GOTERM_BP_FAT | GO:0051130~positive regulation of cellular component organization | | | | | 4 | 1.015228 | 0.800538 | 0.94013 | 1 | 0.999437 | 100 |
| GOTERM_BP_FAT | GO:0032583~regulation of gene-specific transcription | | | | | 3 | 0.761421 | 0.825675 | 0.952408 | 1 | 0.999655 | 100 |
| GOTERM_BP_FAT | GO:0033043~regulation of organelle organization | | | | | 4 | 1.015228 | 0.887193 | 0.784164 | 1 | 0.99994 | 100 |
|  |  | | | | |  |  |  |  |  |  |  |
| Annotation Cluster 66 | Enrichment Score: 0.5185073703263289 |  |  |  | | | | |  |  |  |  |
| Category | Term | | | | | Count | % | PValue | Fold Enrichment | Bonferroni | Benjamini | FDR |
| GOTERM_BP_FAT | GO:0030539~male genitalia development | | | | | 3 | 0.761421 | 0.052913 | 7.976415 | 1 | 0.458051 | 61.10895 |
| GOTERM_BP_FAT | GO:0048806~genitalia development | | | | | 3 | 0.761421 | 0.100355 | 5.548811 | 1 | 0.616821 | 84.07283 |
| GOTERM_BP_FAT | GO:0007548~sex differentiation | | | | | 6 | 1.522843 | 0.280238 | 1.690366 | 1 | 0.883022 | 99.66953 |
| GOTERM_BP_FAT | GO:0045137~development of primary sexual characteristics | | | | | 5 | 1.269036 | 0.347259 | 1.674838 | 1 | 0.926076 | 99.9395 |
| GOTERM_BP_FAT | GO:0046546~development of primary male sexual characteristics | | | | | 3 | 0.761421 | 0.452352 | 1.963425 | 1 | 0.964588 | 99.99713 |
| GOTERM_BP_FAT | GO:0046661~male sex differentiation | | | | | 3 | 0.761421 | 0.513227 | 1.748255 | 1 | 0.977663 | 99.99963 |
| GOTERM_BP_FAT | GO:0008406~gonad development | | | | | 3 | 0.761421 | 0.742237 | 1.139488 | 1 | 0.9986 | 100 |
| GOTERM_BP_FAT | GO:0048608~reproductive structure development | | | | | 3 | 0.761421 | 0.798601 | 1.012878 | 1 | 0.99943 | 100 |
|  |  | | | | |  |  |  |  |  |  |  |
| Annotation Cluster 67 | Enrichment Score: 0.49016171272809145 |  |  |  |  | | | | |  |  |  |
| Category | Term | | | | | Count | % | PValue | Fold Enrichment | Bonferroni | Benjamini | FDR |
| GOTERM_BP_FAT | GO:0031329~regulation of cellular catabolic process | | | | | 4 | 1.015228 | 0.171642 | 2.789566 | 1 | 0.765302 | 96.20416 |
| GOTERM_BP_FAT | GO:0031331~positive regulation of cellular catabolic process | | | | | 3 | 0.761421 | 0.206167 | 3.545073 | 1 | 0.815109 | 98.18816 |
| GOTERM_BP_FAT | GO:0009896~positive regulation of catabolic process | | | | | 3 | 0.761421 | 0.319291 | 2.604544 | 1 | 0.909484 | 99.87461 |
| GOTERM_BP_FAT | GO:0009894~regulation of catabolic process | | | | | 4 | 1.015228 | 0.391542 | 1.772537 | 1 | 0.946464 | 99.98215 |
| GOTERM_BP_FAT | GO:0051130~positive regulation of cellular component organization | | | | | 4 | 1.015228 | 0.800538 | 0.94013 | 1 | 0.999437 | 100 |
|  |  | | | | |  |  |  |  |  |  |  |
| Annotation Cluster 68 | Enrichment Score: 0.4818906819623946 |  |  |  | | | | |  |  |  |  |
| Category | Term | | | | | Count | % | PValue | Fold Enrichment | Bonferroni | Benjamini | FDR |
| GOTERM_BP_FAT | GO:0006928~cell motion | | | | | 18 | 4.568528 | 0.054872 | 1.612075 | 1 | 0.463703 | 62.48302 |
| GOTERM_BP_FAT | GO:0001764~neuron migration | | | | | 3 | 0.761421 | 0.44444 | 1.994104 | 1 | 0.962602 | 99.99632 |
| GOTERM_BP_FAT | GO:0016477~cell migration | | | | | 8 | 2.030457 | 0.470295 | 1.233069 | 1 | 0.968695 | 99.99839 |
| GOTERM_BP_FAT | GO:0051674~localization of cell | | | | | 8 | 2.030457 | 0.58278 | 1.108557 | 1 | 0.988371 | 99.99997 |
| GOTERM_BP_FAT | GO:0048870~cell motility | | | | | 8 | 2.030457 | 0.58278 | 1.108557 | 1 | 0.988371 | 99.99997 |
|  |  | | | | |  |  |  |  |  |  |  |
| Annotation Cluster 69 | Enrichment Score: 0.47512101777229887 |  |  |  |  | | | | |  |  |  |
| Category | Term | | | | | Count | % | PValue | Fold Enrichment | Bonferroni | Benjamini | FDR |
| GOTERM_BP_FAT | GO:0051345~positive regulation of hydrolase activity | | | | | 7 | 1.77665 | 0.243467 | 1.66361 | 1 | 0.853982 | 99.21471 |
| GOTERM_BP_FAT | GO:0043281~regulation of caspase activity | | | | | 4 | 1.015228 | 0.282495 | 2.153969 | 1 | 0.884653 | 99.68708 |
| GOTERM_BP_FAT | GO:0052548~regulation of endopeptidase activity | | | | | 4 | 1.015228 | 0.301734 | 2.075165 | 1 | 0.896596 | 99.80485 |
| GOTERM_BP_FAT | GO:0052547~regulation of peptidase activity | | | | | 4 | 1.015228 | 0.327474 | 1.978646 | 1 | 0.914879 | 99.89837 |
| GOTERM_BP_FAT | GO:0006919~activation of caspase activity | | | | | 3 | 0.761421 | 0.362153 | 2.363382 | 1 | 0.93332 | 99.95949 |
| GOTERM_BP_FAT | GO:0051336~regulation of hydrolase activity | | | | | 10 | 2.538071 | 0.393722 | 1.262341 | 1 | 0.947167 | 99.98323 |
| GOTERM_BP_FAT | GO:0010952~positive regulation of peptidase activity | | | | | 3 | 0.761421 | 0.40397 | 2.163096 | 1 | 0.950632 | 99.98753 |
| GOTERM_BP_FAT | GO:0043280~positive regulation of caspase activity | | | | | 3 | 0.761421 | 0.40397 | 2.163096 | 1 | 0.950632 | 99.98753 |
|  |  | | | | |  |  |  |  |  |  |  |
| Annotation Cluster 70 | Enrichment Score: 0.464020747545222 |  |  |  | | | | |  |  |  |  |
| Category | Term | | | | | Count | % | PValue | Fold Enrichment | Bonferroni | Benjamini | FDR |
| GOTERM_BP_FAT | GO:0007611~learning or memory | | | | | 8 | 2.030457 | 0.015515 | 3.066009 | 1 | 0.207475 | 23.78706 |
| GOTERM_BP_FAT | GO:0050890~cognition | | | | | 17 | 4.314721 | 0.910681 | 0.795594 | 1 | 0.999976 | 100 |
| GOTERM_BP_FAT | GO:0050877~neurological system process | | | | | 19 | 4.822335 | 0.988696 | 0.667997 | 1 | 1 | 100 |
| GOTERM_BP_FAT | GO:0007600~sensory perception | | | | | 10 | 2.538071 | 0.99708 | 0.525196 | 1 | 1 | 100 |
|  |  | | | | |  |  |  |  |  |  |  |
| Annotation Cluster 71 | Enrichment Score: 0.4464123857901319 |  |  |  | | | | |  |  |  |  |
| Category | Term | | | | | Count | % | PValue | Fold Enrichment | Bonferroni | Benjamini | FDR |
| GOTERM_BP_FAT | GO:0016265~death | | | | | 21 | 5.329949 | 0.247345 | 1.233921 | 1 | 0.856452 | 99.28177 |
| GOTERM_BP_FAT | GO:0008219~cell death | | | | | 20 | 5.076142 | 0.322987 | 1.183335 | 1 | 0.911901 | 99.88593 |
| GOTERM_BP_FAT | GO:0006915~apoptosis | | | | | 16 | 4.060914 | 0.442286 | 1.130655 | 1 | 0.962103 | 99.99607 |
| GOTERM_BP_FAT | GO:0012501~programmed cell death | | | | | 16 | 4.060914 | 0.463617 | 1.114 | 1 | 0.96733 | 99.998 |
|  |  | | | | |  |  |  |  |  |  |  |
| Annotation Cluster 72 | Enrichment Score: 0.4096620959709471 |  |  |  | | | | |  |  |  |  |
| Category | Term | | | | | Count | % | PValue | Fold Enrichment | Bonferroni | Benjamini | FDR |
| GOTERM_BP_FAT | GO:0030728~ovulation | | | | | 3 | 0.761421 | 0.04137 | 9.115903 | 1 | 0.399663 | 51.99905 |
| GOTERM_BP_FAT | GO:0003006~reproductive developmental process | | | | | 10 | 2.538071 | 0.163163 | 1.623698 | 1 | 0.752326 | 95.46958 |
| GOTERM_BP_FAT | GO:0007292~female gamete generation | | | | | 4 | 1.015228 | 0.195222 | 2.6179 | 1 | 0.802179 | 97.70156 |
| GOTERM_BP_FAT | GO:0048610~reproductive cellular process | | | | | 4 | 1.015228 | 0.735658 | 1.050392 | 1 | 0.998463 | 100 |
| GOTERM_BP_FAT | GO:0048609~reproductive process in a multicellular organism | | | | | 10 | 2.538071 | 0.810635 | 0.873529 | 1 | 0.99952 | 100 |
| GOTERM_BP_FAT | GO:0032504~multicellular organism reproduction | | | | | 10 | 2.538071 | 0.810635 | 0.873529 | 1 | 0.99952 | 100 |
| GOTERM_BP_FAT | GO:0007276~gamete generation | | | | | 7 | 1.77665 | 0.905045 | 0.753889 | 1 | 0.999969 | 100 |
| GOTERM_BP_FAT | GO:0019953~sexual reproduction | | | | | 8 | 2.030457 | 0.915956 | 0.743072 | 1 | 0.999981 | 100 |
|  |  | | | | |  |  |  |  |  |  |  |
| Annotation Cluster 73 | Enrichment Score: 0.4026231592300878 |  |  |  | | | | |  |  |  |  |
| Category | Term | | | | | Count | % | PValue | Fold Enrichment | Bonferroni | Benjamini | FDR |
| GOTERM_BP_FAT | GO:0009581~detection of external stimulus | | | | | 5 | 1.269036 | 0.095487 | 2.874384 | 1 | 0.606846 | 82.5077 |
| GOTERM_BP_FAT | GO:0045088~regulation of innate immune response | | | | | 4 | 1.015228 | 0.132654 | 3.151176 | 1 | 0.697767 | 91.56084 |
| GOTERM_BP_FAT | GO:0042110~T cell activation | | | | | 6 | 1.522843 | 0.17392 | 2.025756 | 1 | 0.76785 | 96.38149 |
| GOTERM_BP_FAT | GO:0001775~cell activation | | | | | 10 | 2.538071 | 0.232753 | 1.482261 | 1 | 0.841896 | 98.99741 |
| GOTERM_BP_FAT | GO:0045321~leukocyte activation | | | | | 8 | 2.030457 | 0.339962 | 1.40631 | 1 | 0.922004 | 99.92662 |
| GOTERM_BP_FAT | GO:0048872~homeostasis of number of cells | | | | | 4 | 1.015228 | 0.416791 | 1.701635 | 1 | 0.954668 | 99.99145 |
| GOTERM_BP_FAT | GO:0030217~T cell differentiation | | | | | 3 | 0.761421 | 0.452352 | 1.963425 | 1 | 0.964588 | 99.99713 |
| GOTERM_BP_FAT | GO:0046649~lymphocyte activation | | | | | 6 | 1.522843 | 0.501158 | 1.28264 | 1 | 0.975028 | 99.99943 |
| GOTERM_BP_FAT | GO:0030098~lymphocyte differentiation | | | | | 3 | 0.761421 | 0.699278 | 1.239055 | 1 | 0.997268 | 100 |
| GOTERM_BP_FAT | GO:0030097~hemopoiesis | | | | | 5 | 1.269036 | 0.807508 | 0.90129 | 1 | 0.999499 | 100 |
| GOTERM_BP_FAT | GO:0002521~leukocyte differentiation | | | | | 3 | 0.761421 | 0.815928 | 0.974219 | 1 | 0.999565 | 100 |
| GOTERM_BP_FAT | GO:0048534~hemopoietic or lymphoid organ development | | | | | 5 | 1.269036 | 0.862496 | 0.818094 | 1 | 0.999869 | 100 |
| GOTERM_BP_FAT | GO:0002520~immune system development | | | | | 5 | 1.269036 | 0.891213 | 0.770668 | 1 | 0.999947 | 100 |
|  |  | | | | |  |  |  |  |  |  |  |
| Annotation Cluster 74 | Enrichment Score: 0.382769420635986 |  |  |  | | | | |  |  |  |  |
| Category | Term | | | | | Count | % | PValue | Fold Enrichment | Bonferroni | Benjamini | FDR |
| GOTERM_BP_FAT | GO:0030336~negative regulation of cell migration | | | | | 3 | 0.761421 | 0.38739 | 2.238994 | 1 | 0.944708 | 99.97991 |
| GOTERM_BP_FAT | GO:0040013~negative regulation of locomotion | | | | | 3 | 0.761421 | 0.420333 | 2.092174 | 1 | 0.955865 | 99.99231 |
| GOTERM_BP_FAT | GO:0051271~negative regulation of cell motion | | | | | 3 | 0.761421 | 0.436465 | 2.025756 | 1 | 0.960492 | 99.99529 |
|  |  | | | | |  |  |  |  |  |  |  |
| Annotation Cluster 75 | Enrichment Score: 0.36895886569234104 |  |  |  |  | | | | |  |  |  |
| Category | Term | | | | | Count | % | PValue | Fold Enrichment | Bonferroni | Benjamini | FDR |
| GOTERM_BP_FAT | GO:0051345~positive regulation of hydrolase activity | | | | | 7 | 1.77665 | 0.243467 | 1.66361 | 1 | 0.853982 | 99.21471 |
| GOTERM_BP_FAT | GO:0060193~positive regulation of lipase activity | | | | | 3 | 0.761421 | 0.527738 | 1.701635 | 1 | 0.980596 | 99.99978 |
| GOTERM_BP_FAT | GO:0060191~regulation of lipase activity | | | | | 3 | 0.761421 | 0.608505 | 1.466927 | 1 | 0.991403 | 99.99999 |
|  |  | | | | |  |  |  |  |  |  |  |
| Annotation Cluster 76 | Enrichment Score: 0.27214582410897237 |  |  |  |  | | | | |  |  |  |
| Category | Term | | | | | Count | % | PValue | Fold Enrichment | Bonferroni | Benjamini | FDR |
| GOTERM_BP_FAT | GO:0035295~tube development | | | | | 8 | 2.030457 | 0.259316 | 1.546941 | 1 | 0.864745 | 99.45637 |
| GOTERM_BP_FAT | GO:0048754~branching morphogenesis of a tube | | | | | 3 | 0.761421 | 0.452352 | 1.963425 | 1 | 0.964588 | 99.99713 |
| GOTERM_BP_FAT | GO:0001763~morphogenesis of a branching structure | | | | | 3 | 0.761421 | 0.520519 | 1.72463 | 1 | 0.97918 | 99.99972 |
| GOTERM_BP_FAT | GO:0035239~tube morphogenesis | | | | | 3 | 0.761421 | 0.802177 | 1.004903 | 1 | 0.999449 | 100 |
| GOTERM_BP_FAT | GO:0008283~cell proliferation | | | | | 8 | 2.030457 | 0.889731 | 0.780567 | 1 | 0.999944 | 100 |
|  |  | | | | |  |  |  |  |  |  |  |
| Annotation Cluster 77 | Enrichment Score: 0.25183192447081126 |  |  |  |  | | | | |  |  |  |
| Category | Term | | | | | Count | % | PValue | Fold Enrichment | Bonferroni | Benjamini | FDR |
| GOTERM_BP_FAT | GO:0001701~in utero embryonic development | | | | | 6 | 1.522843 | 0.395818 | 1.450257 | 1 | 0.947405 | 99.98421 |
| GOTERM_BP_FAT | GO:0043009~chordate embryonic development | | | | | 8 | 2.030457 | 0.661471 | 1.028178 | 1 | 0.99562 | 100 |
| GOTERM_BP_FAT | GO:0009792~embryonic development ending in birth or egg hatching | | | | | 8 | 2.030457 | 0.670653 | 1.018943 | 1 | 0.996135 | 100 |
|  |  | | | | |  |  |  |  |  |  |  |
| Annotation Cluster 78 | Enrichment Score: 0.21348644798596825 |  |  |  |  | | | | |  |  |  |
| Category | Term | | | | | Count | % | PValue | Fold Enrichment | Bonferroni | Benjamini | FDR |
| GOTERM_BP_FAT | GO:0006814~sodium ion transport | | | | | 5 | 1.269036 | 0.363271 | 1.636188 | 1 | 0.933526 | 99.9607 |
| GOTERM_BP_FAT | GO:0006811~ion transport | | | | | 20 | 5.076142 | 0.432963 | 1.107835 | 1 | 0.959761 | 99.99475 |
| GOTERM_BP_FAT | GO:0006812~cation transport | | | | | 12 | 3.045685 | 0.753118 | 0.923129 | 1 | 0.998812 | 100 |
| GOTERM_BP_FAT | GO:0030001~metal ion transport | | | | | 10 | 2.538071 | 0.766423 | 0.914858 | 1 | 0.999018 | 100 |
| GOTERM_BP_FAT | GO:0015672~monovalent inorganic cation transport | | | | | 5 | 1.269036 | 0.943093 | 0.668882 | 1 | 0.999995 | 100 |
|  |  | | | | |  |  |  |  |  |  |  |
| Annotation Cluster 79 | Enrichment Score: 0.17866101301322754 |  |  |  |  | | | | |  |  |  |
| Category | Term | | | | | Count | % | PValue | Fold Enrichment | Bonferroni | Benjamini | FDR |
| GOTERM_BP_FAT | GO:0019932~second-messenger-mediated signaling | | | | | 7 | 1.77665 | 0.474252 | 1.267175 | 1 | 0.969422 | 99.99859 |
| GOTERM_BP_FAT | GO:0007187~G-protein signaling, coupled to cyclic nucleotide second messenger | | | | | 3 | 0.761421 | 0.755346 | 1.109762 | 1 | 0.998846 | 100 |
| GOTERM_BP_FAT | GO:0019935~cyclic-nucleotide-mediated signaling | | | | | 3 | 0.761421 | 0.812573 | 0.981713 | 1 | 0.999535 | 100 |
|  |  | | | | |  |  |  |  |  |  |  |
| Annotation Cluster 80 | Enrichment Score: 0.11688550552321787 |  |  |  |  | | | | |  |  |  |
| Category | Term | | | | | Count | % | PValue | Fold Enrichment | Bonferroni | Benjamini | FDR |
| GOTERM_BP_FAT | GO:0060341~regulation of cellular localization | | | | | 6 | 1.522843 | 0.694331 | 1.029215 | 1 | 0.997116 | 100 |
| GOTERM_BP_FAT | GO:0051223~regulation of protein transport | | | | | 3 | 0.761421 | 0.751041 | 1.119497 | 1 | 0.998781 | 100 |
| GOTERM_BP_FAT | GO:0070201~regulation of establishment of protein localization | | | | | 3 | 0.761421 | 0.779848 | 1.054733 | 1 | 0.999187 | 100 |
| GOTERM_BP_FAT | GO:0032880~regulation of protein localization | | | | | 3 | 0.761421 | 0.83795 | 0.924802 | 1 | 0.99974 | 100 |
|  |  | | | | |  |  |  |  |  |  |  |
| Annotation Cluster 81 | Enrichment Score: 0.11110006469850997 |  |  |  |  | | | | |  |  |  |
| Category | Term | | | | | Count | % | PValue | Fold Enrichment | Bonferroni | Benjamini | FDR |
| GOTERM_BP_FAT | GO:0031328~positive regulation of cellular biosynthetic process | | | | | 18 | 4.568528 | 0.438974 | 1.117863 | 1 | 0.961149 | 99.99564 |
| GOTERM_BP_FAT | GO:0009891~positive regulation of biosynthetic process | | | | | 18 | 4.568528 | 0.465054 | 1.101778 | 1 | 0.967529 | 99.99809 |
| GOTERM_BP_FAT | GO:0010604~positive regulation of macromolecule metabolic process | | | | | 21 | 5.329949 | 0.541266 | 1.042425 | 1 | 0.982849 | 99.99987 |
| GOTERM_BP_FAT | GO:0010557~positive regulation of macromolecule biosynthetic process | | | | | 16 | 4.060914 | 0.572105 | 1.040755 | 1 | 0.98717 | 99.99996 |
| GOTERM_BP_FAT | GO:0045944~positive regulation of transcription from RNA polymerase II promoter | | | | | 8 | 2.030457 | 0.770828 | 0.917324 | 1 | 0.999061 | 100 |
| GOTERM_BP_FAT | GO:0045893~positive regulation of transcription, DNA-dependent | | | | | 10 | 2.538071 | 0.791373 | 0.891842 | 1 | 0.999338 | 100 |
| GOTERM_BP_FAT | GO:0051254~positive regulation of RNA metabolic process | | | | | 10 | 2.538071 | 0.799243 | 0.884426 | 1 | 0.999429 | 100 |
| GOTERM_BP_FAT | GO:0051173~positive regulation of nitrogen compound metabolic process | | | | | 13 | 3.299492 | 0.830726 | 0.858744 | 1 | 0.999693 | 100 |
| GOTERM_BP_FAT | GO:0045935~positive regulation of nucleobase, nucleoside, nucleotide and nucleic acid metabolic process | | | | | 12 | 3.045685 | 0.870888 | 0.818094 | 1 | 0.999898 | 100 |
| GOTERM_BP_FAT | GO:0006357~regulation of transcription from RNA polymerase II promoter | | | | | 14 | 3.553299 | 0.87654 | 0.819219 | 1 | 0.999915 | 100 |
| GOTERM_BP_FAT | GO:0045941~positive regulation of transcription | | | | | 10 | 2.538071 | 0.91685 | 0.754271 | 1 | 0.999981 | 100 |
| GOTERM_BP_FAT | GO:0010628~positive regulation of gene expression | | | | | 10 | 2.538071 | 0.931832 | 0.732201 | 1 | 0.999991 | 100 |
| GOTERM_BP_FAT | GO:0051252~regulation of RNA metabolic process | | | | | 25 | 6.345178 | 0.999653 | 0.586609 | 1 | 1 | 100 |
| GOTERM_BP_FAT | GO:0006355~regulation of transcription, DNA-dependent | | | | | 24 | 6.091371 | 0.999714 | 0.575849 | 1 | 1 | 100 |
| GOTERM_BP_FAT | GO:0045449~regulation of transcription | | | | | 27 | 6.852792 | 1 | 0.441601 | 1 | 1 | 100 |
| GOTERM_BP_FAT | GO:0006350~transcription | | | | | 13 | 3.299492 | 1 | 0.263223 | 1 | 1 | 100 |
|  |  | | | | |  |  |  |  |  |  |  |
| Annotation Cluster 82 | Enrichment Score: 0.08406186787946626 |  |  |  |  | | | | |  |  |  |
| Category | Term | | | | | Count | % | PValue | Fold Enrichment | Bonferroni | Benjamini | FDR |
| GOTERM_BP_FAT | GO:0050953~sensory perception of light stimulus | | | | | 5 | 1.269036 | 0.749104 | 0.984743 | 1 | 0.998753 | 100 |
| GOTERM_BP_FAT | GO:0007601~visual perception | | | | | 5 | 1.269036 | 0.749104 | 0.984743 | 1 | 0.998753 | 100 |
| GOTERM_BP_FAT | GO:0007600~sensory perception | | | | | 10 | 2.538071 | 0.99708 | 0.525196 | 1 | 1 | 100 |
|  |  | | | | |  |  |  |  |  |  |  |
| Annotation Cluster 83 | Enrichment Score: 0.043472789043135396 |  |  |  |  | | | | |  |  |  |
| Category | Term | | | | | Count | % | PValue | Fold Enrichment | Bonferroni | Benjamini | FDR |
| GOTERM_BP_FAT | GO:0031175~neuron projection development | | | | | 6 | 1.522843 | 0.720505 | 0.997052 | 1 | 0.998011 | 100 |
| GOTERM_BP_FAT | GO:0048666~neuron development | | | | | 7 | 1.77665 | 0.810284 | 0.878425 | 1 | 0.999524 | 100 |
| GOTERM_BP_FAT | GO:0048812~neuron projection morphogenesis | | | | | 4 | 1.015228 | 0.879569 | 0.79889 | 1 | 0.999922 | 100 |
| GOTERM_BP_FAT | GO:0030182~neuron differentiation | | | | | 8 | 2.030457 | 0.89237 | 0.777002 | 1 | 0.999948 | 100 |
| GOTERM_BP_FAT | GO:0000904~cell morphogenesis involved in differentiation | | | | | 4 | 1.015228 | 0.9283 | 0.697391 | 1 | 0.999989 | 100 |
| GOTERM_BP_FAT | GO:0048858~cell projection morphogenesis | | | | | 4 | 1.015228 | 0.929519 | 0.694545 | 1 | 0.99999 | 100 |
| GOTERM_BP_FAT | GO:0030030~cell projection organization | | | | | 6 | 1.522843 | 0.935733 | 0.693601 | 1 | 0.999993 | 100 |
| GOTERM_BP_FAT | GO:0032990~cell part morphogenesis | | | | | 4 | 1.015228 | 0.941731 | 0.664701 | 1 | 0.999995 | 100 |
| GOTERM_BP_FAT | GO:0007409~axonogenesis | | | | | 3 | 0.761421 | 0.943256 | 0.661257 | 1 | 0.999995 | 100 |
| GOTERM_BP_FAT | GO:0048667~cell morphogenesis involved in neuron differentiation | | | | | 3 | 0.761421 | 0.958714 | 0.610635 | 1 | 0.999999 | 100 |
| GOTERM_BP_FAT | GO:0000902~cell morphogenesis | | | | | 5 | 1.269036 | 0.969438 | 0.597484 | 1 | 1 | 100 |
| GOTERM_BP_FAT | GO:0032989~cellular component morphogenesis | | | | | 5 | 1.269036 | 0.984858 | 0.535779 | 1 | 1 | 100 |
|  |  | | | | |  |  |  |  |  |  |  |
| Annotation Cluster 84 | Enrichment Score: 0.030694159488515022 |  |  |  |  | | | | |  |  |  |
| Category | Term | | | | | Count | % | PValue | Fold Enrichment | Bonferroni | Benjamini | FDR |
| GOTERM_BP_FAT | GO:0006468~protein amino acid phosphorylation | | | | | 12 | 3.045685 | 0.917067 | 0.765353 | 1 | 0.999981 | 100 |
| GOTERM_BP_FAT | GO:0006796~phosphate metabolic process | | | | | 18 | 4.568528 | 0.922107 | 0.786984 | 1 | 0.999985 | 100 |
| GOTERM_BP_FAT | GO:0006793~phosphorus metabolic process | | | | | 18 | 4.568528 | 0.922107 | 0.786984 | 1 | 0.999985 | 100 |
| GOTERM_BP_FAT | GO:0016310~phosphorylation | | | | | 13 | 3.299492 | 0.966629 | 0.691289 | 1 | 0.999999 | 100 |
|  |  | | | | |  |  |  |  |  |  |  |
| Annotation Cluster 85 | Enrichment Score: 0.027263302323477748 |  |  |  |  | | | | |  |  |  |
| Category | Term | | | | | Count | % | PValue | Fold Enrichment | Bonferroni | Benjamini | FDR |
| GOTERM_BP_FAT | GO:0030036~actin cytoskeleton organization | | | | | 4 | 1.015228 | 0.902795 | 0.752936 | 1 | 0.999966 | 100 |
| GOTERM_BP_FAT | GO:0030029~actin filament-based process | | | | | 4 | 1.015228 | 0.924525 | 0.706073 | 1 | 0.999987 | 100 |
| GOTERM_BP_FAT | GO:0007010~cytoskeleton organization | | | | | 5 | 1.269036 | 0.992436 | 0.487854 | 1 | 1 | 100 |
|  |  | | | | |  |  |  |  |  |  |  |
| Annotation Cluster 86 | Enrichment Score: 0.025112955164363165 |  |  |  |  | | | | |  |  |  |
| Category | Term | | | | | Count | % | PValue | Fold Enrichment | Bonferroni | Benjamini | FDR |
| GOTERM_BP_FAT | GO:0045892~negative regulation of transcription, DNA-dependent | | | | | 7 | 1.77665 | 0.844837 | 0.836478 | 1 | 0.999782 | 100 |
| GOTERM_BP_FAT | GO:0051253~negative regulation of RNA metabolic process | | | | | 7 | 1.77665 | 0.855749 | 0.822614 | 1 | 0.999841 | 100 |
| GOTERM_BP_FAT | GO:0010605~negative regulation of macromolecule metabolic process | | | | | 13 | 3.299492 | 0.930123 | 0.753449 | 1 | 0.99999 | 100 |
| GOTERM_BP_FAT | GO:0000122~negative regulation of transcription from RNA polymerase II promoter | | | | | 4 | 1.015228 | 0.951105 | 0.639712 | 1 | 0.999997 | 100 |
| GOTERM_BP_FAT | GO:0045934~negative regulation of nucleobase, nucleoside, nucleotide and nucleic acid metabolic process | | | | | 8 | 2.030457 | 0.958732 | 0.664701 | 1 | 0.999999 | 100 |
| GOTERM_BP_FAT | GO:0016481~negative regulation of transcription | | | | | 7 | 1.77665 | 0.96078 | 0.648772 | 1 | 0.999999 | 100 |
| GOTERM_BP_FAT | GO:0051172~negative regulation of nitrogen compound metabolic process | | | | | 8 | 2.030457 | 0.962528 | 0.655736 | 1 | 0.999999 | 100 |
| GOTERM_BP_FAT | GO:0031327~negative regulation of cellular biosynthetic process | | | | | 8 | 2.030457 | 0.979388 | 0.606644 | 1 | 1 | 100 |
| GOTERM_BP_FAT | GO:0010629~negative regulation of gene expression | | | | | 7 | 1.77665 | 0.979957 | 0.590846 | 1 | 1 | 100 |
| GOTERM_BP_FAT | GO:0009890~negative regulation of biosynthetic process | | | | | 8 | 2.030457 | 0.982721 | 0.593939 | 1 | 1 | 100 |
| GOTERM_BP_FAT | GO:0010558~negative regulation of macromolecule biosynthetic process | | | | | 7 | 1.77665 | 0.989787 | 0.544399 | 1 | 1 | 100 |
|  |  | | | | |  |  |  |  |  |  |  |
| Annotation Cluster 87 | Enrichment Score: 0.0017408440777531063 |  |  |  |  | | | | |  |  |  |
| Category | Term | | | | | Count | % | PValue | Fold Enrichment | Bonferroni | Benjamini | FDR |
| GOTERM_BP_FAT | GO:0022402~cell cycle process | | | | | 7 | 1.77665 | 0.992364 | 0.527055 | 1 | 1 | 100 |
| GOTERM_BP_FAT | GO:0022403~cell cycle phase | | | | | 4 | 1.015228 | 0.997032 | 0.411023 | 1 | 1 | 100 |
| GOTERM_BP_FAT | GO:0000278~mitotic cell cycle | | | | | 3 | 0.761421 | 0.998613 | 0.344926 | 1 | 1 | 100 |
|  |  | | | | |  |  |  |  |  |  |  |
| Annotation Cluster 88 | Enrichment Score: 8.872559152716225E-4 |  |  |  |  | | | | |  |  |  |
| Category | Term | | | | | Count | % | PValue | Fold Enrichment | Bonferroni | Benjamini | FDR |
| GOTERM_BP_FAT | GO:0034613~cellular protein localization | | | | | 4 | 1.015228 | 0.996849 | 0.414023 | 1 | 1 | 100 |
| GOTERM_BP_FAT | GO:0070727~cellular macromolecule localization | | | | | 4 | 1.015228 | 0.997032 | 0.411023 | 1 | 1 | 100 |
| GOTERM_BP_FAT | GO:0008104~protein localization | | | | | 5 | 1.269036 | 0.999999 | 0.241161 | 1 | 1 | 100 |
|  |  | | | | |  |  |  |  |  |  |  |
| Annotation Cluster 89 | Enrichment Score: 1.7329871456940457E-7 |  |  |  |  | | | | |  |  |  |
| Category | Term | | | | | Count | % | PValue | Fold Enrichment | Bonferroni | Benjamini | FDR |
| GOTERM_BP_FAT | GO:0008104~protein localization | | | | | 5 | 1.269036 | 0.999999 | 0.241161 | 1 | 1 | 100 |
| GOTERM_BP_FAT | GO:0015031~protein transport | | | | | 3 | 0.761421 | 1 | 0.167484 | 1 | 1 | 100 |
| GOTERM_BP_FAT | GO:0045184~establishment of protein localization | | | | | 3 | 0.761421 | 1 | 0.165959 | 1 | 1 | 100 |
