# Supplementary material for: Long non-coding RNA expression profiles of hepatitis C virus-related dysplasia and hepatocellular carcinoma
Source: Oncotarget. 2015 Oct 26;6(41):43770–8. doi: 10.18632/oncotarget.6087 (PMC4791265; doi:10.18632/oncotarget.6087)
Supplement: Supplementary file 5 [file oncotarget-06-43770-s005.docx]

| Category | Term | Count | % | PValue | Fold Enrichment | Bonferroni | Benjamini | FDR |
| --- | --- | --- | --- | --- | --- | --- | --- | --- |
| GOTERM_BP_FAT | GO:0007067~mitosis | 67 | 11.49228 | 5.86E-44 | 8.747114 | 1.35E-40 | 1.35E-40 | 1.02E-40 |
| GOTERM_BP_FAT | GO:0000280~nuclear division | 67 | 11.49228 | 5.86E-44 | 8.747114 | 1.35E-40 | 1.35E-40 | 1.02E-40 |
| GOTERM_BP_FAT | GO:0048285~organelle fission | 68 | 11.66381 | 7.71E-44 | 8.528764 | 1.77E-40 | 8.84E-41 | 1.35E-40 |
| GOTERM_BP_FAT | GO:0000087~M phase of mitotic cell cycle | 67 | 11.49228 | 2.12E-43 | 8.590916 | 4.86E-40 | 1.62E-40 | 3.70E-40 |
| GOTERM_BP_FAT | GO:0000279~M phase | 77 | 13.20755 | 1.14E-41 | 6.722139 | 2.61E-38 | 6.52E-39 | 1.99E-38 |
| GOTERM_BP_FAT | GO:0000278~mitotic cell cycle | 79 | 13.5506 | 1.04E-39 | 6.132507 | 2.38E-36 | 4.75E-37 | 1.81E-36 |
| GOTERM_BP_FAT | GO:0022403~cell cycle phase | 83 | 14.23671 | 1.25E-39 | 5.758249 | 2.88E-36 | 4.79E-37 | 2.19E-36 |
| GOTERM_BP_FAT | GO:0007049~cell cycle | 110 | 18.86792 | 3.17E-38 | 4.071399 | 7.27E-35 | 1.04E-35 | 5.53E-35 |
| GOTERM_BP_FAT | GO:0022402~cell cycle process | 92 | 15.78045 | 1.88E-36 | 4.676835 | 4.32E-33 | 5.40E-34 | 3.29E-33 |
| GOTERM_BP_FAT | GO:0051301~cell division | 67 | 11.49228 | 3.01E-35 | 6.523272 | 6.91E-32 | 7.68E-33 | 5.26E-32 |
| GOTERM_BP_FAT | GO:0007059~chromosome segregation | 29 | 4.974271 | 4.79E-21 | 10.28314 | 1.10E-17 | 1.10E-18 | 8.37E-18 |
| GOTERM_BP_FAT | GO:0007017~microtubule-based process | 41 | 7.03259 | 6.07E-16 | 4.654532 | 1.27E-12 | 1.16E-13 | 9.66E-13 |
| GOTERM_BP_FAT | GO:0000226~microtubule cytoskeleton organization | 31 | 5.317324 | 2.59E-15 | 6.056993 | 5.86E-12 | 4.88E-13 | 4.46E-12 |
| GOTERM_BP_FAT | GO:0007051~spindle organization | 19 | 3.259005 | 2.82E-15 | 12.12701 | 6.37E-12 | 4.90E-13 | 4.85E-12 |
| GOTERM_BP_FAT | GO:0006259~DNA metabolic process | 57 | 9.777015 | 9.75E-15 | 3.235467 | 2.24E-11 | 1.60E-12 | 1.71E-11 |
| GOTERM_BP_FAT | GO:0006260~DNA replication | 34 | 5.831904 | 1.44E-14 | 5.139703 | 3.31E-11 | 2.21E-12 | 2.52E-11 |
| GOTERM_BP_FAT | GO:0000070~mitotic sister chromatid segregation | 15 | 2.572899 | 5.81E-12 | 11.96745 | 1.33E-08 | 8.33E-10 | 1.01E-08 |
| GOTERM_BP_FAT | GO:0000819~sister chromatid segregation | 15 | 2.572899 | 9.05E-12 | 11.644 | 2.08E-08 | 1.22E-09 | 1.58E-08 |
| GOTERM_BP_FAT | GO:0051726~regulation of cell cycle | 40 | 6.861063 | 2.10E-11 | 3.470921 | 4.82E-08 | 2.68E-09 | 3.67E-08 |
| GOTERM_BP_FAT | GO:0051276~chromosome organization | 47 | 8.06175 | 5.47E-10 | 2.783356 | 1.26E-06 | 6.61E-08 | 9.56E-07 |
| GOTERM_BP_FAT | GO:0000075~cell cycle checkpoint | 19 | 3.259005 | 1.78E-09 | 5.996874 | 4.10E-06 | 2.05E-07 | 3.12E-06 |
| GOTERM_BP_FAT | GO:0007346~regulation of mitotic cell cycle | 24 | 4.116638 | 2.58E-09 | 4.535032 | 5.93E-06 | 2.82E-07 | 4.51E-06 |
| GOTERM_BP_FAT | GO:0007010~cytoskeleton organization | 42 | 7.204117 | 6.44E-09 | 2.766785 | 1.48E-05 | 6.72E-07 | 1.12E-05 |
| GOTERM_BP_FAT | GO:0006261~DNA-dependent DNA replication | 15 | 2.572899 | 7.69E-09 | 7.428069 | 1.76E-05 | 7.67E-07 | 1.34E-05 |
| GOTERM_BP_FAT | GO:0008283~cell proliferation | 39 | 6.689537 | 1.74E-07 | 2.569158 | 4.00E-04 | 1.67E-05 | 3.04E-04 |
| GOTERM_BP_FAT | GO:0006270~DNA replication initiation | 8 | 1.372213 | 5.09E-07 | 14.36093 | 0.001168 | 4.68E-05 | 8.90E-04 |
| GOTERM_BP_FAT | GO:0006323~DNA packaging | 18 | 3.087479 | 5.69E-07 | 4.418749 | 0.001305 | 5.02E-05 | 9.94E-04 |
| GOTERM_BP_FAT | GO:0006974~response to DNA damage stimulus | 34 | 5.831904 | 8.23E-07 | 2.618079 | 0.001887 | 7.00E-05 | 0.001438 |
| GOTERM_BP_FAT | GO:0051297~centrosome organization | 10 | 1.715266 | 9.40E-07 | 8.975584 | 0.002154 | 7.70E-05 | 0.001641 |
| GOTERM_BP_FAT | GO:0030261~chromosome condensation | 9 | 1.543739 | 1.28E-06 | 10.33987 | 0.002944 | 1.02E-04 | 0.002244 |
| GOTERM_BP_FAT | GO:0007093~mitotic cell cycle checkpoint | 11 | 1.886792 | 1.59E-06 | 7.347455 | 0.003648 | 1.22E-04 | 0.002782 |
| GOTERM_BP_FAT | GO:0010564~regulation of cell cycle process | 17 | 2.915952 | 1.96E-06 | 4.283086 | 0.004488 | 1.45E-04 | 0.003424 |
| GOTERM_BP_FAT | GO:0031023~microtubule organizing center organization | 10 | 1.715266 | 2.16E-06 | 8.206248 | 0.004935 | 1.55E-04 | 0.003765 |
| GOTERM_BP_FAT | GO:0007076~mitotic chromosome condensation | 7 | 1.200686 | 2.38E-06 | 15.46562 | 0.005436 | 1.65E-04 | 0.004149 |
| GOTERM_BP_FAT | GO:0051640~organelle localization | 15 | 2.572899 | 3.24E-06 | 4.682913 | 0.007405 | 2.19E-04 | 0.005657 |
| GOTERM_BP_FAT | GO:0051656~establishment of organelle localization | 13 | 2.229846 | 3.91E-06 | 5.411367 | 0.008944 | 2.57E-04 | 0.006838 |
| GOTERM_BP_FAT | GO:0007052~mitotic spindle organization | 7 | 1.200686 | 6.53E-06 | 13.40354 | 0.014869 | 4.16E-04 | 0.011402 |
| GOTERM_BP_FAT | GO:0006281~DNA repair | 26 | 4.459691 | 1.96E-05 | 2.629467 | 0.043976 | 0.001215 | 0.034226 |
| GOTERM_BP_FAT | GO:0034508~centromere complex assembly | 5 | 0.857633 | 2.04E-05 | 23.93489 | 0.045769 | 0.001232 | 0.035655 |
| GOTERM_BP_FAT | GO:0007098~centrosome cycle | 7 | 1.200686 | 3.14E-05 | 10.58174 | 0.069583 | 0.001848 | 0.054884 |
| GOTERM_BP_FAT | GO:0033554~cellular response to stress | 40 | 6.861063 | 3.57E-05 | 2.029814 | 0.078643 | 0.002046 | 0.062328 |
| GOTERM_BP_FAT | GO:0048015~phosphoinositide-mediated signaling | 13 | 2.229846 | 5.02E-05 | 4.243003 | 0.108769 | 0.002805 | 0.087614 |
| GOTERM_BP_FAT | GO:0051303~establishment of chromosome localization | 6 | 1.02916 | 1.11E-04 | 11.48875 | 0.225716 | 0.006072 | 0.194536 |
| GOTERM_BP_FAT | GO:0050000~chromosome localization | 6 | 1.02916 | 1.11E-04 | 11.48875 | 0.225716 | 0.006072 | 0.194536 |
| GOTERM_BP_FAT | GO:0007018~microtubule-based movement | 14 | 2.401372 | 1.47E-04 | 3.558462 | 0.286913 | 0.007833 | 0.257067 |
| GOTERM_BP_FAT | GO:0043933~macromolecular complex subunit organization | 44 | 7.54717 | 2.71E-04 | 1.779947 | 0.462689 | 0.014018 | 0.471719 |
| GOTERM_BP_FAT | GO:0006271~DNA strand elongation during DNA replication | 4 | 0.686106 | 3.95E-04 | 22.97749 | 0.596607 | 0.019972 | 0.688661 |
| GOTERM_BP_FAT | GO:0034404~nucleobase, nucleoside and nucleotide biosynthetic process | 18 | 3.087479 | 4.10E-04 | 2.678723 | 0.609833 | 0.020253 | 0.71386 |
| GOTERM_BP_FAT | GO:0034654~nucleobase, nucleoside, nucleotide and nucleic acid biosynthetic process | 18 | 3.087479 | 4.10E-04 | 2.678723 | 0.609833 | 0.020253 | 0.71386 |
| GOTERM_BP_FAT | GO:0000910~cytokinesis | 8 | 1.372213 | 4.71E-04 | 5.604267 | 0.661046 | 0.022756 | 0.820145 |
| GOTERM_BP_FAT | GO:0046651~lymphocyte proliferation | 8 | 1.372213 | 5.49E-04 | 5.470832 | 0.716358 | 0.025909 | 0.95455 |
| GOTERM_BP_FAT | GO:0031577~spindle checkpoint | 5 | 0.857633 | 5.70E-04 | 11.96745 | 0.729858 | 0.026357 | 0.991309 |
| GOTERM_BP_FAT | GO:0033043~regulation of organelle organization | 19 | 3.259005 | 5.77E-04 | 2.514818 | 0.734196 | 0.026152 | 1.003509 |
| GOTERM_BP_FAT | GO:0007088~regulation of mitosis | 9 | 1.543739 | 6.56E-04 | 4.616015 | 0.778083 | 0.029087 | 1.139398 |
| GOTERM_BP_FAT | GO:0051783~regulation of nuclear division | 9 | 1.543739 | 6.56E-04 | 4.616015 | 0.778083 | 0.029087 | 1.139398 |
| GOTERM_BP_FAT | GO:0051321~meiotic cell cycle | 12 | 2.058319 | 6.91E-04 | 3.446624 | 0.795216 | 0.030036 | 1.199842 |
| GOTERM_BP_FAT | GO:0034621~cellular macromolecular complex subunit organization | 26 | 4.459691 | 7.05E-04 | 2.091789 | 0.801903 | 0.030085 | 1.224808 |
| GOTERM_BP_FAT | GO:0070661~leukocyte proliferation | 8 | 1.372213 | 7.34E-04 | 5.222158 | 0.814752 | 0.030741 | 1.275213 |
| GOTERM_BP_FAT | GO:0032943~mononuclear cell proliferation | 8 | 1.372213 | 7.34E-04 | 5.222158 | 0.814752 | 0.030741 | 1.275213 |
| GOTERM_BP_FAT | GO:0022616~DNA strand elongation | 4 | 0.686106 | 7.71E-04 | 19.14791 | 0.829524 | 0.031655 | 1.337644 |
| GOTERM_BP_FAT | GO:0009165~nucleotide biosynthetic process | 17 | 2.915952 | 7.78E-04 | 2.625117 | 0.832332 | 0.031386 | 1.350117 |
| GOTERM_BP_FAT | GO:0030071~regulation of mitotic metaphase/anaphase transition | 6 | 1.02916 | 7.99E-04 | 7.833237 | 0.840363 | 0.031678 | 1.386967 |
| GOTERM_BP_FAT | GO:0051329~interphase of mitotic cell cycle | 12 | 2.058319 | 8.86E-04 | 3.346237 | 0.869321 | 0.034478 | 1.537101 |
| GOTERM_BP_FAT | GO:0006268~DNA unwinding during replication | 5 | 0.857633 | 0.001091 | 10.25781 | 0.918311 | 0.041566 | 1.888599 |
| GOTERM_BP_FAT | GO:0051325~interphase | 12 | 2.058319 | 0.001126 | 3.251532 | 0.924594 | 0.042166 | 1.948347 |
| GOTERM_BP_FAT | GO:0065004~protein-DNA complex assembly | 11 | 1.886792 | 0.001206 | 3.471874 | 0.937318 | 0.044389 | 2.086194 |
| GOTERM_BP_FAT | GO:0006302~double-strand break repair | 9 | 1.543739 | 0.001302 | 4.169303 | 0.949696 | 0.047076 | 2.250013 |
| GOTERM_BP_FAT | GO:0065003~macromolecular complex assembly | 39 | 6.689537 | 0.001763 | 1.68444 | 0.982553 | 0.062242 | 3.034753 |
| GOTERM_BP_FAT | GO:0010605~negative regulation of macromolecule metabolic process | 42 | 7.204117 | 0.001827 | 1.643486 | 0.984957 | 0.063472 | 3.144139 |
| GOTERM_BP_FAT | GO:0051340~regulation of ligase activity | 10 | 1.715266 | 0.001939 | 3.54591 | 0.988371 | 0.066231 | 3.333688 |
| GOTERM_BP_FAT | GO:0031570~DNA integrity checkpoint | 8 | 1.372213 | 0.00202 | 4.418749 | 0.990344 | 0.067891 | 3.470418 |
| GOTERM_BP_FAT | GO:0009263~deoxyribonucleotide biosynthetic process | 4 | 0.686106 | 0.002048 | 14.36093 | 0.990949 | 0.067814 | 3.51798 |
| GOTERM_BP_FAT | GO:0040001~establishment of mitotic spindle localization | 4 | 0.686106 | 0.002048 | 14.36093 | 0.990949 | 0.067814 | 3.51798 |
| GOTERM_BP_FAT | GO:0010639~negative regulation of organelle organization | 10 | 1.715266 | 0.002113 | 3.502667 | 0.992201 | 0.068891 | 3.627226 |
| GOTERM_BP_FAT | GO:0007126~meiosis | 11 | 1.886792 | 0.00212 | 3.223883 | 0.992326 | 0.068145 | 3.639037 |
| GOTERM_BP_FAT | GO:0051327~M phase of meiotic cell cycle | 11 | 1.886792 | 0.00212 | 3.223883 | 0.992326 | 0.068145 | 3.639037 |
| GOTERM_BP_FAT | GO:0044271~nitrogen compound biosynthetic process | 23 | 3.945111 | 0.002246 | 2.032625 | 0.99426 | 0.071067 | 3.851848 |
| GOTERM_BP_FAT | GO:0032392~DNA geometric change | 5 | 0.857633 | 0.002986 | 7.978297 | 0.998955 | 0.09215 | 5.090668 |
| GOTERM_BP_FAT | GO:0032508~DNA duplex unwinding | 5 | 0.857633 | 0.002986 | 7.978297 | 0.998955 | 0.09215 | 5.090668 |
| GOTERM_BP_FAT | GO:0051439~regulation of ubiquitin-protein ligase activity during mitotic cell cycle | 9 | 1.543739 | 0.003123 | 3.6408 | 0.999238 | 0.094902 | 5.318185 |
| GOTERM_BP_FAT | GO:0045842~positive regulation of mitotic metaphase/anaphase transition | 3 | 0.51458 | 0.00353 | 28.72187 | 0.999701 | 0.105228 | 5.991282 |
| GOTERM_BP_FAT | GO:0031055~chromatin remodeling at centromere | 3 | 0.51458 | 0.00353 | 28.72187 | 0.999701 | 0.105228 | 5.991282 |
| GOTERM_BP_FAT | GO:0034622~cellular macromolecular complex assembly | 22 | 3.773585 | 0.003742 | 1.987047 | 0.999816 | 0.109755 | 6.338837 |
| GOTERM_BP_FAT | GO:0032269~negative regulation of cellular protein metabolic process | 15 | 2.572899 | 0.004105 | 2.393489 | 0.999921 | 0.118282 | 6.93442 |
| GOTERM_BP_FAT | GO:0051293~establishment of spindle localization | 4 | 0.686106 | 0.004166 | 11.48875 | 0.999931 | 0.11845 | 7.033817 |
| GOTERM_BP_FAT | GO:0051653~spindle localization | 4 | 0.686106 | 0.004166 | 11.48875 | 0.999931 | 0.11845 | 7.033817 |
| GOTERM_BP_FAT | GO:0051438~regulation of ubiquitin-protein ligase activity | 9 | 1.543739 | 0.005579 | 3.314062 | 0.999997 | 0.153586 | 9.310926 |
| GOTERM_BP_FAT | GO:0045841~negative regulation of mitotic metaphase/anaphase transition | 4 | 0.686106 | 0.005582 | 10.44432 | 0.999997 | 0.151854 | 9.315976 |
| GOTERM_BP_FAT | GO:0007094~mitotic cell cycle spindle assembly checkpoint | 4 | 0.686106 | 0.005582 | 10.44432 | 0.999997 | 0.151854 | 9.315976 |
| GOTERM_BP_FAT | GO:0051310~metaphase plate congression | 4 | 0.686106 | 0.005582 | 10.44432 | 0.999997 | 0.151854 | 9.315976 |
| GOTERM_BP_FAT | GO:0051248~negative regulation of protein metabolic process | 15 | 2.572899 | 0.005741 | 2.303893 | 0.999998 | 0.154029 | 9.569294 |
| GOTERM_BP_FAT | GO:0006221~pyrimidine nucleotide biosynthetic process | 5 | 0.857633 | 0.006397 | 6.527697 | 1 | 0.16815 | 10.60542 |
| GOTERM_BP_FAT | GO:0032886~regulation of microtubule-based process | 7 | 1.200686 | 0.006718 | 4.103124 | 1 | 0.173859 | 11.10884 |
| GOTERM_BP_FAT | GO:0051383~kinetochore organization | 3 | 0.51458 | 0.006898 | 21.5414 | 1 | 0.176127 | 11.3902 |
| GOTERM_BP_FAT | GO:0007144~female meiosis I | 3 | 0.51458 | 0.006898 | 21.5414 | 1 | 0.176127 | 11.3902 |
| GOTERM_BP_FAT | GO:0000212~meiotic spindle organization | 3 | 0.51458 | 0.006898 | 21.5414 | 1 | 0.176127 | 11.3902 |
| GOTERM_BP_FAT | GO:0007096~regulation of exit from mitosis | 4 | 0.686106 | 0.007253 | 9.573956 | 1 | 0.182312 | 11.94097 |
| GOTERM_BP_FAT | GO:0045839~negative regulation of mitosis | 4 | 0.686106 | 0.007253 | 9.573956 | 1 | 0.182312 | 11.94097 |
| GOTERM_BP_FAT | GO:0007143~female meiosis | 4 | 0.686106 | 0.007253 | 9.573956 | 1 | 0.182312 | 11.94097 |
| GOTERM_BP_FAT | GO:0051784~negative regulation of nuclear division | 4 | 0.686106 | 0.007253 | 9.573956 | 1 | 0.182312 | 11.94097 |
| GOTERM_BP_FAT | GO:0006220~pyrimidine nucleotide metabolic process | 6 | 1.02916 | 0.007678 | 4.786978 | 1 | 0.189885 | 12.59738 |
| GOTERM_BP_FAT | GO:0019932~second-messenger-mediated signaling | 17 | 2.915952 | 0.008066 | 2.077752 | 1 | 0.196399 | 13.19199 |
| GOTERM_BP_FAT | GO:0031400~negative regulation of protein modification process | 11 | 1.886792 | 0.008474 | 2.654963 | 1 | 0.203155 | 13.81396 |
| GOTERM_BP_FAT | GO:0001556~oocyte maturation | 4 | 0.686106 | 0.009188 | 8.837498 | 1 | 0.216121 | 14.89254 |
| GOTERM_BP_FAT | GO:0010948~negative regulation of cell cycle process | 5 | 0.857633 | 0.010192 | 5.744374 | 1 | 0.234457 | 16.3864 |
| GOTERM_BP_FAT | GO:0031497~chromatin assembly | 9 | 1.543739 | 0.010624 | 2.971228 | 1 | 0.240754 | 17.02186 |
| GOTERM_BP_FAT | GO:0060249~anatomical structure homeostasis | 10 | 1.715266 | 0.011446 | 2.70961 | 1 | 0.254385 | 18.21698 |
| GOTERM_BP_FAT | GO:0051351~positive regulation of ligase activity | 8 | 1.372213 | 0.013224 | 3.147602 | 1 | 0.285178 | 20.74892 |
| GOTERM_BP_FAT | GO:0006284~base-excision repair | 5 | 0.857633 | 0.013391 | 5.318865 | 1 | 0.285591 | 20.98318 |
| GOTERM_BP_FAT | GO:0045787~positive regulation of cell cycle | 7 | 1.200686 | 0.013832 | 3.527247 | 1 | 0.290878 | 21.59854 |
| GOTERM_BP_FAT | GO:0070507~regulation of microtubule cytoskeleton organization | 6 | 1.02916 | 0.014627 | 4.103124 | 1 | 0.302147 | 22.69462 |
| GOTERM_BP_FAT | GO:0051983~regulation of chromosome segregation | 3 | 0.51458 | 0.016465 | 14.36093 | 1 | 0.3304 | 25.17587 |
| GOTERM_BP_FAT | GO:0006297~nucleotide-excision repair, DNA gap filling | 4 | 0.686106 | 0.019712 | 6.758087 | 1 | 0.378708 | 29.37609 |
| GOTERM_BP_FAT | GO:0051225~spindle assembly | 4 | 0.686106 | 0.019712 | 6.758087 | 1 | 0.378708 | 29.37609 |
| GOTERM_BP_FAT | GO:0046649~lymphocyte activation | 14 | 2.401372 | 0.021744 | 2.020634 | 1 | 0.405558 | 31.88981 |
| GOTERM_BP_FAT | GO:0001894~tissue homeostasis | 7 | 1.200686 | 0.021794 | 3.191319 | 1 | 0.403117 | 31.95118 |
| GOTERM_BP_FAT | GO:0045321~leukocyte activation | 16 | 2.744425 | 0.022118 | 1.898967 | 1 | 0.40458 | 32.34325 |
| GOTERM_BP_FAT | GO:0032297~negative regulation of DNA replication initiation | 3 | 0.51458 | 0.022526 | 12.30937 | 1 | 0.407187 | 32.83441 |
| GOTERM_BP_FAT | GO:0000076~DNA replication checkpoint | 3 | 0.51458 | 0.022526 | 12.30937 | 1 | 0.407187 | 32.83441 |
| GOTERM_BP_FAT | GO:0006677~glycosylceramide metabolic process | 3 | 0.51458 | 0.022526 | 12.30937 | 1 | 0.407187 | 32.83441 |
| GOTERM_BP_FAT | GO:0043066~negative regulation of apoptosis | 21 | 3.602058 | 0.022759 | 1.70384 | 1 | 0.40733 | 33.11364 |
| GOTERM_BP_FAT | GO:0031396~regulation of protein ubiquitination | 9 | 1.543739 | 0.022995 | 2.584968 | 1 | 0.407519 | 33.3959 |
| GOTERM_BP_FAT | GO:0016447~somatic recombination of immunoglobulin gene segments | 4 | 0.686106 | 0.023058 | 6.382637 | 1 | 0.405345 | 33.46998 |
| GOTERM_BP_FAT | GO:0009127~purine nucleoside monophosphate biosynthetic process | 4 | 0.686106 | 0.023058 | 6.382637 | 1 | 0.405345 | 33.46998 |
| GOTERM_BP_FAT | GO:0009168~purine ribonucleoside monophosphate biosynthetic process | 4 | 0.686106 | 0.023058 | 6.382637 | 1 | 0.405345 | 33.46998 |
| GOTERM_BP_FAT | GO:0009116~nucleoside metabolic process | 7 | 1.200686 | 0.023369 | 3.141454 | 1 | 0.406558 | 33.83977 |
| GOTERM_BP_FAT | GO:0000077~DNA damage checkpoint | 6 | 1.02916 | 0.024883 | 3.590234 | 1 | 0.423482 | 35.60839 |
| GOTERM_BP_FAT | GO:0031145~anaphase-promoting complex-dependent proteasomal ubiquitin-dependent protein catabolic process | 7 | 1.200686 | 0.025019 | 3.093124 | 1 | 0.422222 | 35.76476 |
| GOTERM_BP_FAT | GO:0010638~positive regulation of organelle organization | 8 | 1.372213 | 0.025149 | 2.768373 | 1 | 0.420914 | 35.91447 |
| GOTERM_BP_FAT | GO:0043069~negative regulation of programmed cell death | 21 | 3.602058 | 0.025934 | 1.680109 | 1 | 0.427858 | 36.81024 |
| GOTERM_BP_FAT | GO:0060548~negative regulation of cell death | 21 | 3.602058 | 0.026593 | 1.675442 | 1 | 0.433052 | 37.55254 |
| GOTERM_BP_FAT | GO:0006541~glutamine metabolic process | 4 | 0.686106 | 0.026691 | 6.046709 | 1 | 0.431318 | 37.66248 |
| GOTERM_BP_FAT | GO:0006163~purine nucleotide metabolic process | 13 | 2.229846 | 0.029155 | 2.007442 | 1 | 0.457608 | 40.36311 |
| GOTERM_BP_FAT | GO:0016601~Rac protein signal transduction | 3 | 0.51458 | 0.029352 | 10.7707 | 1 | 0.456893 | 40.57345 |
| GOTERM_BP_FAT | GO:0006461~protein complex assembly | 27 | 4.631218 | 0.029569 | 1.535625 | 1 | 0.456432 | 40.80574 |
| GOTERM_BP_FAT | GO:0070271~protein complex biogenesis | 27 | 4.631218 | 0.029569 | 1.535625 | 1 | 0.456432 | 40.80574 |
| GOTERM_BP_FAT | GO:0051437~positive regulation of ubiquitin-protein ligase activity during mitotic cell cycle | 7 | 1.200686 | 0.030427 | 2.956663 | 1 | 0.463155 | 41.71275 |
| GOTERM_BP_FAT | GO:0009167~purine ribonucleoside monophosphate metabolic process | 4 | 0.686106 | 0.030611 | 5.744374 | 1 | 0.46229 | 41.90626 |
| GOTERM_BP_FAT | GO:0009126~purine nucleoside monophosphate metabolic process | 4 | 0.686106 | 0.030611 | 5.744374 | 1 | 0.46229 | 41.90626 |
| GOTERM_BP_FAT | GO:0006333~chromatin assembly or disassembly | 10 | 1.715266 | 0.032925 | 2.261564 | 1 | 0.484376 | 44.28207 |
| GOTERM_BP_FAT | GO:0009064~glutamine family amino acid metabolic process | 6 | 1.02916 | 0.033781 | 3.314062 | 1 | 0.490369 | 45.13652 |
| GOTERM_BP_FAT | GO:0051443~positive regulation of ubiquitin-protein ligase activity | 7 | 1.200686 | 0.034426 | 2.872187 | 1 | 0.494072 | 45.77364 |
| GOTERM_BP_FAT | GO:0048599~oocyte development | 4 | 0.686106 | 0.034816 | 5.470832 | 1 | 0.495116 | 46.15503 |
| GOTERM_BP_FAT | GO:0030174~regulation of DNA replication initiation | 3 | 0.51458 | 0.036883 | 9.573956 | 1 | 0.512623 | 48.13353 |
| GOTERM_BP_FAT | GO:0002566~somatic diversification of immune receptors via somatic mutation | 3 | 0.51458 | 0.036883 | 9.573956 | 1 | 0.512623 | 48.13353 |
| GOTERM_BP_FAT | GO:0016446~somatic hypermutation of immunoglobulin genes | 3 | 0.51458 | 0.036883 | 9.573956 | 1 | 0.512623 | 48.13353 |
| GOTERM_BP_FAT | GO:0007080~mitotic metaphase plate congression | 3 | 0.51458 | 0.036883 | 9.573956 | 1 | 0.512623 | 48.13353 |
| GOTERM_BP_FAT | GO:0033205~cytokinesis during cell cycle | 3 | 0.51458 | 0.036883 | 9.573956 | 1 | 0.512623 | 48.13353 |
| GOTERM_BP_FAT | GO:0051298~centrosome duplication | 3 | 0.51458 | 0.036883 | 9.573956 | 1 | 0.512623 | 48.13353 |
| GOTERM_BP_FAT | GO:0000079~regulation of cyclin-dependent protein kinase activity | 6 | 1.02916 | 0.038882 | 3.191319 | 1 | 0.528672 | 49.98307 |
| GOTERM_BP_FAT | GO:0051172~negative regulation of nitrogen compound metabolic process | 27 | 4.631218 | 0.039189 | 1.494201 | 1 | 0.528593 | 50.26089 |
| GOTERM_BP_FAT | GO:0016445~somatic diversification of immunoglobulins | 4 | 0.686106 | 0.039304 | 5.222158 | 1 | 0.526759 | 50.36472 |
| GOTERM_BP_FAT | GO:0009994~oocyte differentiation | 4 | 0.686106 | 0.039304 | 5.222158 | 1 | 0.526759 | 50.36472 |
| GOTERM_BP_FAT | GO:0006289~nucleotide-excision repair | 6 | 1.02916 | 0.0416 | 3.133295 | 1 | 0.544522 | 52.39696 |
| GOTERM_BP_FAT | GO:0006325~chromatin organization | 21 | 3.602058 | 0.041893 | 1.595659 | 1 | 0.544211 | 52.65028 |
| GOTERM_BP_FAT | GO:0001775~cell activation | 17 | 2.915952 | 0.042971 | 1.701295 | 1 | 0.550669 | 53.57246 |
| GOTERM_BP_FAT | GO:0009156~ribonucleoside monophosphate biosynthetic process | 4 | 0.686106 | 0.044069 | 4.995108 | 1 | 0.557116 | 54.4945 |
| GOTERM_BP_FAT | GO:0044092~negative regulation of molecular function | 19 | 3.259005 | 0.04407 | 1.633879 | 1 | 0.5543 | 54.4956 |
| GOTERM_BP_FAT | GO:0000082~G1/S transition of mitotic cell cycle | 6 | 1.02916 | 0.04443 | 3.077343 | 1 | 0.554487 | 54.79335 |
| GOTERM_BP_FAT | GO:0006457~protein folding | 12 | 2.058319 | 0.044918 | 1.947245 | 1 | 0.555734 | 55.19515 |
| GOTERM_BP_FAT | GO:0006303~double-strand break repair via nonhomologous end joining | 3 | 0.51458 | 0.045062 | 8.616561 | 1 | 0.554153 | 55.31294 |
| GOTERM_BP_FAT | GO:0051053~negative regulation of DNA metabolic process | 5 | 0.857633 | 0.045411 | 3.682291 | 1 | 0.554259 | 55.59753 |
| GOTERM_BP_FAT | GO:0042113~B cell activation | 7 | 1.200686 | 0.048398 | 2.645435 | 1 | 0.575153 | 57.96332 |
| GOTERM_BP_FAT | GO:0002562~somatic diversification of immune receptors via germline recombination within a single locus | 4 | 0.686106 | 0.049108 | 4.786978 | 1 | 0.577859 | 58.50766 |
| GOTERM_BP_FAT | GO:0016444~somatic cell DNA recombination | 4 | 0.686106 | 0.049108 | 4.786978 | 1 | 0.577859 | 58.50766 |
| GOTERM_BP_FAT | GO:0009150~purine ribonucleotide metabolic process | 10 | 1.715266 | 0.051352 | 2.081295 | 1 | 0.59188 | 60.18546 |
| GOTERM_BP_FAT | GO:0050658~RNA transport | 8 | 1.372213 | 0.051843 | 2.368814 | 1 | 0.592754 | 60.54377 |
| GOTERM_BP_FAT | GO:0051236~establishment of RNA localization | 8 | 1.372213 | 0.051843 | 2.368814 | 1 | 0.592754 | 60.54377 |
| GOTERM_BP_FAT | GO:0050657~nucleic acid transport | 8 | 1.372213 | 0.051843 | 2.368814 | 1 | 0.592754 | 60.54377 |
| GOTERM_BP_FAT | GO:0042127~regulation of cell proliferation | 37 | 6.346484 | 0.053433 | 1.350329 | 1 | 0.60144 | 61.68387 |
| GOTERM_BP_FAT | GO:0051785~positive regulation of nuclear division | 4 | 0.686106 | 0.054414 | 4.595499 | 1 | 0.605639 | 62.37217 |
| GOTERM_BP_FAT | GO:0009161~ribonucleoside monophosphate metabolic process | 4 | 0.686106 | 0.054414 | 4.595499 | 1 | 0.605639 | 62.37217 |
| GOTERM_BP_FAT | GO:0045840~positive regulation of mitosis | 4 | 0.686106 | 0.054414 | 4.595499 | 1 | 0.605639 | 62.37217 |
| GOTERM_BP_FAT | GO:0045934~negative regulation of nucleobase, nucleoside, nucleotide and nucleic acid metabolic process | 26 | 4.459691 | 0.054743 | 1.458532 | 1 | 0.60526 | 62.59979 |
| GOTERM_BP_FAT | GO:0010212~response to ionizing radiation | 6 | 1.02916 | 0.056875 | 2.872187 | 1 | 0.617074 | 64.04669 |
| GOTERM_BP_FAT | GO:0006403~RNA localization | 8 | 1.372213 | 0.05925 | 2.297749 | 1 | 0.629961 | 65.59608 |
| GOTERM_BP_FAT | GO:0042770~DNA damage response, signal transduction | 7 | 1.200686 | 0.059404 | 2.513163 | 1 | 0.628344 | 65.69408 |
| GOTERM_BP_FAT | GO:0051129~negative regulation of cellular component organization | 10 | 1.715266 | 0.059455 | 2.022667 | 1 | 0.626085 | 65.72631 |
| GOTERM_BP_FAT | GO:0043086~negative regulation of catalytic activity | 16 | 2.744425 | 0.059902 | 1.659025 | 1 | 0.626366 | 66.01001 |
| GOTERM_BP_FAT | GO:0006687~glycosphingolipid metabolic process | 4 | 0.686106 | 0.059983 | 4.418749 | 1 | 0.624333 | 66.06112 |
| GOTERM_BP_FAT | GO:0031100~organ regeneration | 4 | 0.686106 | 0.059983 | 4.418749 | 1 | 0.624333 | 66.06112 |
| GOTERM_BP_FAT | GO:0045786~negative regulation of cell cycle | 7 | 1.200686 | 0.06237 | 2.482137 | 1 | 0.636624 | 67.53566 |
| GOTERM_BP_FAT | GO:0045931~positive regulation of mitotic cell cycle | 3 | 0.51458 | 0.063151 | 7.180467 | 1 | 0.638843 | 68.00491 |
| GOTERM_BP_FAT | GO:0043161~proteasomal ubiquitin-dependent protein catabolic process | 8 | 1.372213 | 0.064533 | 2.252696 | 1 | 0.644577 | 68.81934 |
| GOTERM_BP_FAT | GO:0010498~proteasomal protein catabolic process | 8 | 1.372213 | 0.064533 | 2.252696 | 1 | 0.644577 | 68.81934 |
| GOTERM_BP_FAT | GO:0009262~deoxyribonucleotide metabolic process | 4 | 0.686106 | 0.065807 | 4.255092 | 1 | 0.649536 | 69.55291 |
| GOTERM_BP_FAT | GO:0002200~somatic diversification of immune receptors | 4 | 0.686106 | 0.065807 | 4.255092 | 1 | 0.649536 | 69.55291 |
| GOTERM_BP_FAT | GO:0048736~appendage development | 8 | 1.372213 | 0.067278 | 2.230825 | 1 | 0.655485 | 70.37968 |
| GOTERM_BP_FAT | GO:0060173~limb development | 8 | 1.372213 | 0.067278 | 2.230825 | 1 | 0.655485 | 70.37968 |
| GOTERM_BP_FAT | GO:0000022~mitotic spindle elongation | 2 | 0.343053 | 0.068281 | 28.72187 | 1 | 0.658672 | 70.93113 |
| GOTERM_BP_FAT | GO:0051231~spindle elongation | 2 | 0.343053 | 0.068281 | 28.72187 | 1 | 0.658672 | 70.93113 |
| GOTERM_BP_FAT | GO:0031508~centromeric heterochromatin formation | 2 | 0.343053 | 0.068281 | 28.72187 | 1 | 0.658672 | 70.93113 |
| GOTERM_BP_FAT | GO:0051571~positive regulation of histone H3-K4 methylation | 2 | 0.343053 | 0.068281 | 28.72187 | 1 | 0.658672 | 70.93113 |
| GOTERM_BP_FAT | GO:0030952~establishment or maintenance of cytoskeleton polarity | 2 | 0.343053 | 0.068281 | 28.72187 | 1 | 0.658672 | 70.93113 |
| GOTERM_BP_FAT | GO:0070828~heterochromatin organization | 2 | 0.343053 | 0.068281 | 28.72187 | 1 | 0.658672 | 70.93113 |
| GOTERM_BP_FAT | GO:0055098~response to low density lipoprotein stimulus | 2 | 0.343053 | 0.068281 | 28.72187 | 1 | 0.658672 | 70.93113 |
| GOTERM_BP_FAT | GO:0030951~establishment or maintenance of microtubule cytoskeleton polarity | 2 | 0.343053 | 0.068281 | 28.72187 | 1 | 0.658672 | 70.93113 |
| GOTERM_BP_FAT | GO:0007079~mitotic chromosome movement towards spindle pole | 2 | 0.343053 | 0.068281 | 28.72187 | 1 | 0.658672 | 70.93113 |
| GOTERM_BP_FAT | GO:0031507~heterochromatin formation | 2 | 0.343053 | 0.068281 | 28.72187 | 1 | 0.658672 | 70.93113 |
| GOTERM_BP_FAT | GO:0046519~sphingoid metabolic process | 5 | 0.857633 | 0.07012 | 3.191319 | 1 | 0.666355 | 71.91757 |
| GOTERM_BP_FAT | GO:0009259~ribonucleotide metabolic process | 10 | 1.715266 | 0.070679 | 1.953869 | 1 | 0.66697 | 72.21104 |
| GOTERM_BP_FAT | GO:0006334~nucleosome assembly | 7 | 1.200686 | 0.071785 | 2.393489 | 1 | 0.67048 | 72.78277 |
| GOTERM_BP_FAT | GO:0031398~positive regulation of protein ubiquitination | 7 | 1.200686 | 0.071785 | 2.393489 | 1 | 0.67048 | 72.78277 |
| GOTERM_BP_FAT | GO:0008156~negative regulation of DNA replication | 4 | 0.686106 | 0.071879 | 4.103124 | 1 | 0.668613 | 72.83128 |
| GOTERM_BP_FAT | GO:0031109~microtubule polymerization or depolymerization | 3 | 0.51458 | 0.072963 | 6.628123 | 1 | 0.671943 | 73.37997 |
| GOTERM_BP_FAT | GO:0032107~regulation of response to nutrient levels | 3 | 0.51458 | 0.072963 | 6.628123 | 1 | 0.671943 | 73.37997 |
| GOTERM_BP_FAT | GO:0032104~regulation of response to extracellular stimulus | 3 | 0.51458 | 0.072963 | 6.628123 | 1 | 0.671943 | 73.37997 |
| GOTERM_BP_FAT | GO:0006310~DNA recombination | 8 | 1.372213 | 0.072976 | 2.188333 | 1 | 0.669675 | 73.38659 |
| GOTERM_BP_FAT | GO:0006164~purine nucleotide biosynthetic process | 10 | 1.715266 | 0.073072 | 1.940667 | 1 | 0.667853 | 73.43489 |
| GOTERM_BP_FAT | GO:0009119~ribonucleoside metabolic process | 5 | 0.857633 | 0.074792 | 3.121942 | 1 | 0.674389 | 74.28294 |
| GOTERM_BP_FAT | GO:0051436~negative regulation of ubiquitin-protein ligase activity during mitotic cell cycle | 6 | 1.02916 | 0.074968 | 2.651249 | 1 | 0.672991 | 74.36828 |
| GOTERM_BP_FAT | GO:0048871~multicellular organismal homeostasis | 7 | 1.200686 | 0.075095 | 2.36533 | 1 | 0.671353 | 74.42933 |
| GOTERM_BP_FAT | GO:0046128~purine ribonucleoside metabolic process | 4 | 0.686106 | 0.078193 | 3.961637 | 1 | 0.68445 | 75.88509 |
| GOTERM_BP_FAT | GO:0042278~purine nucleoside metabolic process | 4 | 0.686106 | 0.078193 | 3.961637 | 1 | 0.68445 | 75.88509 |
| GOTERM_BP_FAT | GO:0019220~regulation of phosphate metabolic process | 24 | 4.116638 | 0.081906 | 1.421288 | 1 | 0.699766 | 77.52699 |
| GOTERM_BP_FAT | GO:0051174~regulation of phosphorus metabolic process | 24 | 4.116638 | 0.081906 | 1.421288 | 1 | 0.699766 | 77.52699 |
| GOTERM_BP_FAT | GO:0051352~negative regulation of ligase activity | 6 | 1.02916 | 0.082984 | 2.572108 | 1 | 0.702485 | 77.98333 |
| GOTERM_BP_FAT | GO:0051444~negative regulation of ubiquitin-protein ligase activity | 6 | 1.02916 | 0.082984 | 2.572108 | 1 | 0.702485 | 77.98333 |
| GOTERM_BP_FAT | GO:0000726~non-recombinational repair | 3 | 0.51458 | 0.083224 | 6.154686 | 1 | 0.701381 | 78.08384 |
| GOTERM_BP_FAT | GO:0030183~B cell differentiation | 5 | 0.857633 | 0.084593 | 2.991861 | 1 | 0.705352 | 78.64873 |
| GOTERM_BP_FAT | GO:0033044~regulation of chromosome organization | 4 | 0.686106 | 0.084739 | 3.829582 | 1 | 0.703837 | 78.7081 |
| GOTERM_BP_FAT | GO:0002377~immunoglobulin production | 4 | 0.686106 | 0.084739 | 3.829582 | 1 | 0.703837 | 78.7081 |
| GOTERM_BP_FAT | GO:0002440~production of molecular mediator of immune response | 4 | 0.686106 | 0.09151 | 3.706048 | 1 | 0.730461 | 81.29848 |
| GOTERM_BP_FAT | GO:0006664~glycolipid metabolic process | 4 | 0.686106 | 0.09151 | 3.706048 | 1 | 0.730461 | 81.29848 |
| GOTERM_BP_FAT | GO:0031110~regulation of microtubule polymerization or depolymerization | 4 | 0.686106 | 0.09151 | 3.706048 | 1 | 0.730461 | 81.29848 |
| GOTERM_BP_FAT | GO:0008272~sulfate transport | 3 | 0.51458 | 0.093891 | 5.744374 | 1 | 0.737872 | 82.13652 |
| GOTERM_BP_FAT | GO:0009124~nucleoside monophosphate biosynthetic process | 5 | 0.857633 | 0.094987 | 2.872187 | 1 | 0.740079 | 82.51005 |
| GOTERM_BP_FAT | GO:0016568~chromatin modification | 15 | 2.572899 | 0.097019 | 1.572365 | 1 | 0.745811 | 83.18373 |
| GOTERM_BP_FAT | GO:0015931~nucleobase, nucleoside, nucleotide and nucleic acid transport | 8 | 1.372213 | 0.098526 | 2.033407 | 1 | 0.749426 | 83.66732 |
| GOTERM_BP_FAT | GO:0051130~positive regulation of cellular component organization | 11 | 1.886792 | 0.099983 | 1.745528 | 1 | 0.752775 | 84.12234 |
