# Supplementary material for: Long non-coding RNA expression profiles of hepatitis C virus-related dysplasia and hepatocellular carcinoma
Source: Oncotarget. 2015 Oct 26;6(41):43770–8. doi: 10.18632/oncotarget.6087 (PMC4791265; doi:10.18632/oncotarget.6087)
Supplement: Supplementary file 6 [file oncotarget-06-43770-s006.docx]

| Category | Term | Count | % | PValue | Fold Enrichment | Bonferroni | Benjamini | FDR |
| --- | --- | --- | --- | --- | --- | --- | --- | --- |
| GOTERM_BP_FAT | GO:0055114~oxidation reduction | 12 | 18.75 | 8.81E-06 | 5.292644757 | 0.004104985 | 0.004105 | 0.012562 |
| GOTERM_BP_FAT | GO:0051186~cofactor metabolic process | 6 | 9.375 | 5.55E-04 | 8.671794872 | 0.228303347 | 0.1215373 | 0.788359 |
| GOTERM_BP_FAT | GO:0046395~carboxylic acid catabolic process | 5 | 7.8125 | 5.84E-04 | 12.6951952 | 0.238602378 | 0.0868604 | 0.82906 |
| GOTERM_BP_FAT | GO:0016054~organic acid catabolic process | 5 | 7.8125 | 5.84E-04 | 12.6951952 | 0.238602378 | 0.0868604 | 0.82906 |
| GOTERM_BP_FAT | GO:0006732~coenzyme metabolic process | 5 | 7.8125 | 0.001923 | 9.210239651 | 0.592969464 | 0.2012576 | 2.707792 |
| GOTERM_BP_FAT | GO:0009611~response to wounding | 8 | 12.5 | 0.002195 | 4.25408805 | 0.641710004 | 0.1855828 | 3.08603 |
| GOTERM_BP_FAT | GO:0002526~acute inflammatory response | 4 | 6.25 | 0.004746 | 11.50340136 | 0.891557852 | 0.3094428 | 6.559516 |
| GOTERM_BP_FAT | GO:0006631~fatty acid metabolic process | 5 | 7.8125 | 0.004858 | 7.117003367 | 0.897118172 | 0.2773874 | 6.709601 |
| GOTERM_BP_FAT | GO:0006954~inflammatory response | 6 | 9.375 | 0.005209 | 5.203076923 | 0.912758236 | 0.2627911 | 7.178226 |
| GOTERM_BP_FAT | GO:0006955~immune response | 8 | 12.5 | 0.009242 | 3.26763285 | 0.986912019 | 0.3823199 | 12.40294 |
| GOTERM_BP_FAT | GO:0006565~L-serine catabolic process | 2 | 3.125 | 0.010387 | 187.8888889 | 0.992374797 | 0.3859197 | 13.83632 |
| GOTERM_BP_FAT | GO:0006952~defense response | 7 | 10.9375 | 0.018964 | 3.207859079 | 0.999869129 | 0.5564058 | 23.89575 |
| GOTERM_BP_FAT | GO:0009063~cellular amino acid catabolic process | 3 | 4.6875 | 0.02327 | 12.43382353 | 0.999983224 | 0.6000032 | 28.52366 |
| GOTERM_BP_FAT | GO:0006563~L-serine metabolic process | 2 | 3.125 | 0.027466 | 70.45833333 | 0.999997753 | 0.6322865 | 32.77987 |
| GOTERM_BP_FAT | GO:0009310~amine catabolic process | 3 | 4.6875 | 0.030013 | 10.83974359 | 0.99999934 | 0.6381364 | 35.24814 |
| GOTERM_BP_FAT | GO:0009070~serine family amino acid biosynthetic process | 2 | 3.125 | 0.037574 | 51.24242424 | 0.999999983 | 0.6964888 | 42.08577 |
| GOTERM_BP_FAT | GO:0009071~serine family amino acid catabolic process | 2 | 3.125 | 0.037574 | 51.24242424 | 0.999999983 | 0.6964888 | 42.08577 |
| GOTERM_BP_FAT | GO:0006071~glycerol metabolic process | 2 | 3.125 | 0.076988 | 24.50724638 | 1 | 0.9035076 | 68.10043 |
| GOTERM_BP_FAT | GO:0006099~tricarboxylic acid cycle | 2 | 3.125 | 0.076988 | 24.50724638 | 1 | 0.9035076 | 68.10043 |
| GOTERM_BP_FAT | GO:0046356~acetyl-CoA catabolic process | 2 | 3.125 | 0.076988 | 24.50724638 | 1 | 0.9035076 | 68.10043 |
| GOTERM_BP_FAT | GO:0045087~innate immune response | 3 | 4.6875 | 0.082964 | 6.126811594 | 1 | 0.9073743 | 70.92298 |
| GOTERM_BP_FAT | GO:0006094~gluconeogenesis | 2 | 3.125 | 0.083401 | 22.54666667 | 1 | 0.8955854 | 71.1201 |
| GOTERM_BP_FAT | GO:0019400~alditol metabolic process | 2 | 3.125 | 0.083401 | 22.54666667 | 1 | 0.8955854 | 71.1201 |
| GOTERM_BP_FAT | GO:0009109~coenzyme catabolic process | 2 | 3.125 | 0.086592 | 21.67948718 | 1 | 0.8920592 | 72.52114 |
| GOTERM_BP_FAT | GO:0009069~serine family amino acid metabolic process | 2 | 3.125 | 0.086592 | 21.67948718 | 1 | 0.8920592 | 72.52114 |
| GOTERM_BP_FAT | GO:0015980~energy derivation by oxidation of organic compounds | 3 | 4.6875 | 0.089206 | 5.871527778 | 1 | 0.8871592 | 73.62158 |
| GOTERM_BP_FAT | GO:0006635~fatty acid beta-oxidation | 2 | 3.125 | 0.09294 | 20.13095238 | 1 | 0.8857369 | 75.12288 |
| GOTERM_BP_FAT | GO:0006091~generation of precursor metabolites and energy | 4 | 6.25 | 0.094595 | 3.60170394 | 1 | 0.87869 | 75.76232 |
| GOTERM_BP_FAT | GO:0006958~complement activation, classical pathway | 2 | 3.125 | 0.096098 | 19.43678161 | 1 | 0.8714495 | 76.33 |
